# Multi-Dimensional Flow Cytometry Analyses Reveal a Dichotomous Role for Nitric Oxide in Melanoma Patients Receiving Immunotherapy

**DOI:** 10.3389/fimmu.2020.00164

**Published:** 2020-02-25

**Authors:** Saurabh K. Garg, Matthew J. Ott, A. G. M. Mostofa, Zhihua Chen, Y. Ann Chen, Jodi Kroeger, Biwei Cao, Adam W. Mailloux, Alisha Agrawal, Braydon J. Schaible, Amod Sarnaik, Jeffrey S. Weber, Anders E. Berglund, James J. Mulé, Joseph Markowitz

**Affiliations:** ^1^Department of Cutaneous Oncology, H. Lee Moffitt Cancer Center and Research Institute, Tampa, FL, United States; ^2^Cancer Informatics Core, H. Lee Moffitt Cancer Center and Research Institute, Tampa, FL, United States; ^3^Department of Biostatistics and Bioinformatics, H. Lee Moffitt Cancer Center and Research Institute, Tampa, FL, United States; ^4^Flow Cytometry Core, H. Lee Moffitt Cancer Center and Research Institute, Tampa, FL, United States; ^5^Department of Immunology, H. Lee Moffitt Cancer Center and Research Institute, Tampa, FL, United States; ^6^Department of Oncologic Sciences, USF Health Morsani College of Medicine, University of South Florida, Tampa, FL, United States; ^7^Department of Medicine, Laura and Isaac Perlmutter Cancer Center, NYU Langone Health, New York, NY, United States

**Keywords:** melanoma, nitric oxide, immune response, ipilimumab, flow cytometry

## Abstract

Phenotyping of immune cell subsets in clinical trials is limited to well-defined phenotypes, due to technological limitations of reporting flow cytometry multi-dimensional phenotyping data. We developed a multi-dimensional phenotyping analysis tool and applied it to detect nitric oxide (NO) levels in peripheral blood immune cells before and after adjuvant ipilimumab co-administration with a peptide vaccine in melanoma patients. We analyzed inhibitory and stimulatory markers for immune cell phenotypes that were felt to be important in the NO analysis. The pipeline allows visualization of immune cell phenotypes without knowledge of clustering techniques and to categorize cells by association with relapse-free survival (RFS). Using this analysis, we uncovered the potential for a dichotomous role of NO as a pro- and anti-melanoma factor. NO was found in subsets of immune-suppressor cells associated with shorter-term (≤ 1 year) RFS, whereas NO was also present in immune-stimulatory effector cells obtained from patients with significant longer-term (> 1 year) RFS. These studies provide insights into the cell-specific immunomodulatory role of NO. The methods presented herein can be applied to monitor the pro- and anti-tumor effects of a variety of immune-based therapeutics in cancer patients.

**Clinical Trial Registration Number:** NCT00084656 (https://clinicaltrials.gov/ct2/show/NCT00084656).

## Introduction

Monoclonal antibodies against cytotoxic T-lymphocyte-associated antigen 4 (CTLA-4), ipilimumab, and programmed cell death protein 1 (PD-1), pembrolizumab and nivolumab, are approved for patients with advanced melanoma (1). However, response rates for ipilumumab and nivolumab in melanoma patients are 11-22% and 31-44%, respectively (2, 3). Both types of checkpoint blockade are now Food and Drug Administration-approved for patients who have undergone surgery for metastatic melanoma, although it is unclear which patients require adjuvant therapy and which would benefit from waiting to start checkpoint blockade until at least one melanoma metastasis is visible on traditional imaging. Therefore, tools are needed to assess how effective the therapies are likely to be in different clinical scenarios.

Peripheral blood contains many types of immune cells that can be monitored and demonstrate change with therapy. High-dimensional flow cytometry phenotyping can be performed and analyzed via clustering algorithms including SPADE (Spanning-tree Progression Analysis of Density-normalized Events), t-SNE (t-Distributed Stochastic Neighbor Embedding), and viSNE (visualization tool for t-SNE) (4–6). However, the outputs of these algorithms require manual curation based on marker expression for individual cells. To overcome this limitation, we phenotyped patient samples prior to and after adjuvant ipilimumab with a peptide vaccine treatment and developed a tool called the Multi-Dimensional Phenotyping Analysis Tool in R (MPATR) to analyze associations between cell phenotypes and relapse-free survival (RFS). A multi-dimensional flow cytometry panel was developed to assess the algorithm and test the pro- and anti-tumor associations of nitric oxide (NO) levels in immune-suppressive or stimulatory peripheral blood immune cells. NO levels were measured in a broad range of immune cell subsets as NO and its metabolites have been shown to be elevated in immune suppressor cells derived from patients receiving anti-CTLA-4 therapy (7, 8). While NO has traditionally been associated with immune-suppressive activity in clinical studies, we have recently described the evidence for NO-meditated pro- and anti-tumor function via the activity of myeloid-derived suppressor cells (MDSCs), dendritic cells (DCs), cytotoxic T cells, and natural killer (NK) cells (9). The phenotyping tool described herein allowed for the analyses of high dimensional phenotyping data of immune cells associated with different levels of NO. This analysis is readily applicable to clinical trials by allowing for efficient unsupervised organization of distinct cell phenotypes.

## Materials and Methods

### Patient Samples

Seventy-nine cryopreserved PBMC samples from patients with resected stage IIIc/IV melanoma were provided by Moffitt Cancer Center. Patients were treated with ipilimumab (3-10 mg/kg every 6-8 weeks for 12 months) and 3 separate subcutaneous vaccine injections, as previously described in the clinical trial publication (10). The current analyses used matched samples from 35 of these patients that were taken before and about 13 weeks after ipilimumab treatment initiation, 9 unmatched samples that were collected from melanoma patients before immunotherapy, and 7 PBMC samples that were isolated from normal/healthy individuals. The clinical responses (RFS and overall survival) of these patients were recorded in the primary clinical trial study (10). Collection and handling of all human biological samples were conducted by following the “good clinical practice” (GCP) guidelines.

### Flow Cytometric Analysis of Peripheral Blood Samples

PBMCs were obtained from the blood samples by ficoll density-gradient centrifugation. Patient samples were available from leukapheresis specimens collected at the time of the clinical trial. Frozen PBMCs were used in this retrospective study. Two flow cytometry panels were constructed: myeloid and lymphoid. PBMCs were stained with the antibodies, after proper titration to obtain an optimal signal-to-noise ratio (myeloid panel: DAF-FM [NO marker; Fisher, Hampton, MA], HLA-DR-PE-Cy7, CD33-APC, CD11b-BV421, CD14-BUV395, CD15-BV510, and CD11c-PE [BD Biosciences, San Jose, CA]; lymphoid panel: DAF-FM, along with CD3-BUV395, CD8-BV510, CD11c-PE, CD56-BV421 [BD Biosciences] and CD4-AF700, CD19-PE-Dazzle, CD25-PE-Cy7, CD127-APC [Biolegend, San Diego, CA]). All the antibodies, fluorochromes and controls for the secondary panel including IFNγ, PD-L1, CTLA4, Arginase 1, FoxP3, TCR-ζ, and CD69 to measure immunosuppressive/immunostimulatory properties of the cells are presented in the supplement (Figures S1–S4, Table S1). Dead cells were excluded with Zombie NIR (BioLegend) staining. Data acquisition (100,000 live events) was performed by using an LSRII flow cytometer or FACSymphony (BD Biosciences) and immunophenotypic analysis by FCS Express 6 software (De Novo Software, Pasadena, CA). The gates for each phenotype determined by the unsupervised clustering were utilized to demonstrate the cell populations in the FCS Express visualization application. Positive and negative gates for each antibody were set with fluorescence-minus-one and antibody isotype controls. Rainbow fluorescent particles (BD Biosciences) were also used to calibrate the cytometer correctly between all runs, and flow cytometric compensation beads (Fisher) were used to establish robust compensation matrices.

### Measurement of pSTAT1

Frozen PBMCs were thawed in a water bath at 37°C, washed to remove the freezing media, and allowed to rest overnight in complete media at 5% CO_2_ at 37°C. Stimulation with IFNα was accomplished by replacing the resting media with fresh media containing various concentrations of IFNα and incubating for 15 min. The live/dead marker Zombie NIR (Biolegend, San Diego, CA) was used prior to permeabilization to prevent inappropriate uptake of the dye. After live/dead staining and wash, the samples were permeabilized using the FIX PERM cell permabilization kit methanol modification (Fisher, Hampton, MA). In short, the cells were fixed and preserved while stored at −20°C for a minimum of 2 h then permeabilized for pSTAT1 staining phospho-STAT1 AF4888 (BD Biosciences, San Jose, CA) was applied while the cells were being permeabilized for 1 h at room temperature. Samples were read on an LSR II flow cytometer, and 100,000 live cell events were recorded. Controls included: flow cytometric compensation beads (Fisher) to establish robust compensation matrices, fluorescence-minus-one controls to set negative and positive gates, and isotype controls for patient variations.

### Analyses

Nine pre-treatment only and 35 matched pre-/post-adjuvant ipilimumab and vaccine treatment PBMC samples from patients with resected stage IIIc/IV melanoma were available for statistical analyses. Another 7 PBMC samples from individuals without disease were also collected and used as a “normal” quality control population in each run. In total, there were 44 pre-treatment samples, 35 post-treatment samples, and 7 normal samples. The output from the first step of the clustering analysis (SPADE) created 200 nodes for the 2 different flow cytometry panels (lymphoid, myeloid). A second clustering analysis generated 200 nodes (cell populations) in which the FSC and SSC areas were used as additional clustering parameters to sort cells based on size and granularity. FCS Express 6 was utilized to visualize the phenotypes. After all the analyses were complete, the flow cytometry files were re-analyzed to see if the clustering algorithm can place all the cells in the same nodes (Figure S5). This second step serves as a quality control measure and allows for the possibility of the user to superimpose future experiments onto an existing tree which was not needed in the analyses presented in this paper. The resulting cell populations were normalized by the total number of cells per sample and analyzed in log2 scale before application to parametric tests. Combat, a de-batching method, was performed to remove potential experimental batch effects and was followed by visual confirmation using a principal component analysis. To identify which populations (nodes) were associated with RFS, 2 sets of analyses were performed: Cox proportional-hazard model (Cox regression) and Wilcoxon rank sum test. Cox regression was performed to evaluate cell populations associated with RFS. RFS was defined as the time from study enrollment to time of relapse and was censored at the last clinic appointment. Wilcoxon rank sum test was the second analysis. Principal Component Analysis (PCA) was used to analyze the results (11, 12). Partial Least Squares Projection to Latent Structures (PLS) model was calculated using the cell count in each node as variables and the RFS as a response variable (13). Cross-validation was used to estimate the number of PLS components (14). The weight vector (W1) was used for depicting the importance for each node (15). The progression status for each patient was based on a clinically relevant empirical definition (≤ 1 year RFS vs. >1 year RFS). We investigated whether either pre-treatment level, post-treatment level, or the level of change of each cell population differed between patients with disease relapse and those without. To identify whether therapy alone alters percentages of immune cells in the peripheral blood, the Wilcoxon rank sum test was used. Statistical analyses were performed in the program Rstudio (16). The output was utilized as a score to determine which nodes were associated with RFS suitable for downstream analysis. The same analyses were performed for the datasets obtained using FSC and SSC in the clustering algorithm.

## Results

Patients with resected stage III/IV melanoma were treated with ipilimumab plus a peptide vaccine (10). Pre- and post-treatment peripheral blood mononuclear cells (PBMCs) drawn at week 13 of treatment were available for analysis (9 patients had pre-treatment samples only, 35 patients had both pre- and post-treatment samples; Table S2) (10). As a control for this patient population, we measured interferon response protein STAT1 phosphorylation levels. As previously shown in the literature, pSTAT1 levels were higher in melanoma patients with longer-term RFS (Figure S6, *p* = 0.001, Wilcoxon rank sum test) (17). High-dimensional flow cytometry analyses of patient PBMC samples were performed using lymphoid and myeloid panels that used DAF-FM as the NO stain (Lymphoid panel: DAF-FM, CD3, CD4, CD8, CD25, CD127, CD56, CD19, and CD11c; Myeloid panel: DAF-FM, HLA-DR, CD33, CD11b, CD14, CD15, and CD11c; Table S1). Additional panels were constructed without DAF-FM but included IFNγ, PD-L1, CTLA-4, Arginase 1, FoxP3 for both myeloid/lymphoid panels and TCR-ζ/CD69 in the lymphoid panel (Table S1). Cells were stained, fixed in 1% paraformaldehyde, and analyzed on an LSR II flow cytometer (100,000 live events) using standard gates, isotype control antibodies, and compensation beads to establish criteria for positive staining and compensation controls. The 488 nm Blue laser was reserved for DAF-FM, due to its extreme signal intensity, thus necessitating that the remaining antibodies use all other available lasers (405 nm Violet, 640 nm Red, 561 nm Yellow/Green, 355 nm Ultraviolet).

### MPATR Algorithm

**Figure 1:**
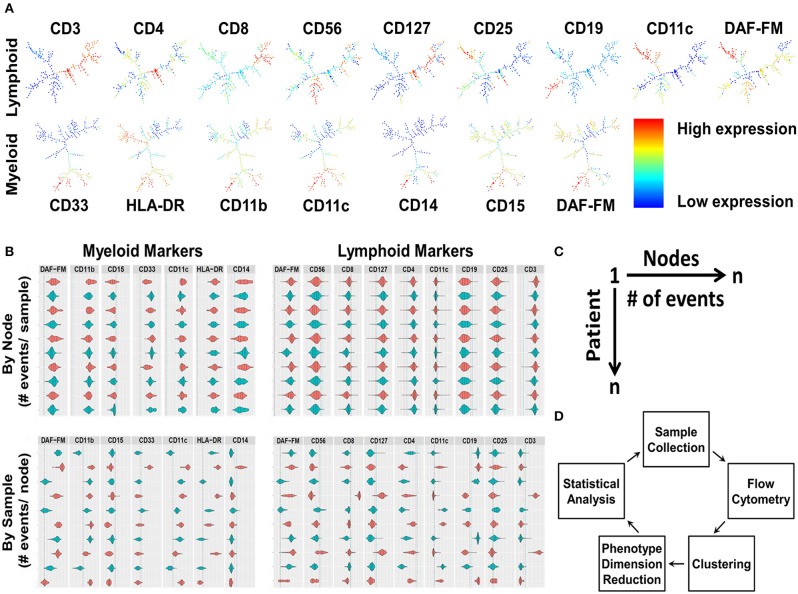
The development and integration of the Multi-Dimensional Phenotype Analysis Tool in R (MPATR) algorithm, using patient samples**. (A)** Phenotypic clustering by SPADE algorithm is the first step of MPATR to phenotype the cells for nitric oxide. The heirachical phenotypic tree illustrates node specific expression of an individual markers (red = high expression; blue = low expression), and number of events in a node of a representative PBMC sample. **(B)** The second step was to visualize the clustering in a user-friendly way by using violin plots that were constructed with positive/negative cut-offs from a sample for each of the markers. The red and blue color denotes alternate patient/node in “by node” (phenotype) and “by sample” panel, respectively. Alternate colors are utilized to facilitate visualization. Positive/negative cutoff was demonstrated by blue and red dotted lines (99 and 95% confidence intervals), respectively. The area underneath the curve of the violin plots represents number of events for the marker. Each row is labeled by the node/sample number and the number of events in that node/sample. **(C)** Schematic of the third step of MPATR illustrating the dimension reduction. In this step, the number of events within each node is placed on a worksheet to facilitate downstream statistical analysis. Each node is associated with a number of events for each sample. With an associated survival time, statistical analysis may be performed. **(D)** Flowchart demonstrating all the major steps of MAPTR algorithm to associate phenotypes with outcome (RFS).

**Figure 2:**
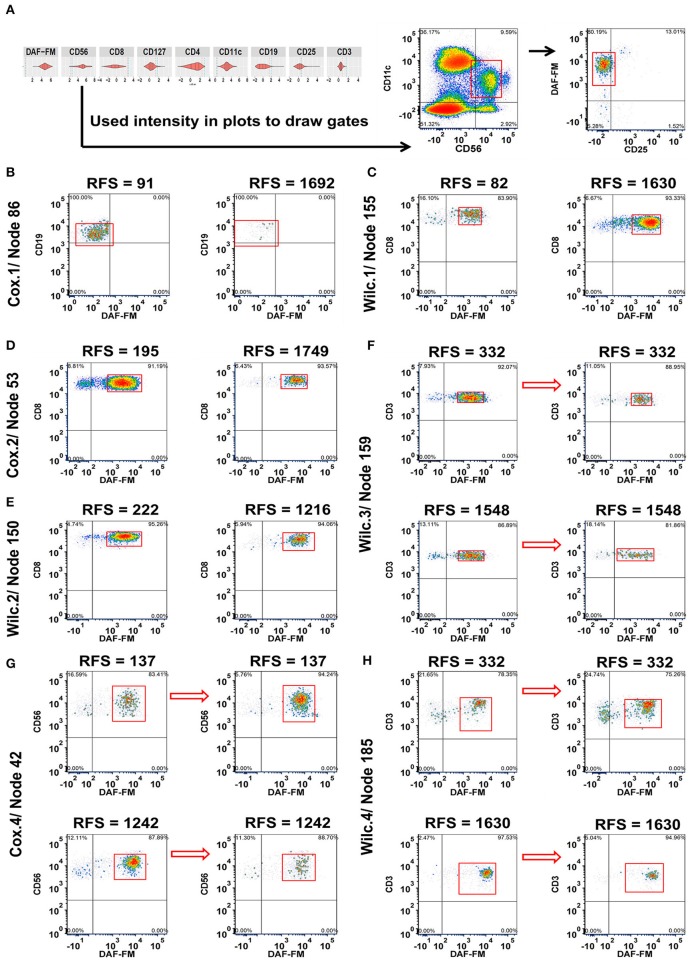
Integration of MPATR output for lymphoid markers and delineation of cellular subsets associated with relapse-free survival (RFS) or treatment effects. **(A)** Unsupervised gating using the cut-offs for each marker generated from the MPATR violin plots (as shown for node 42) to delineate cellular subsets not easily found in bivariate plots. **(B)** Cox.1 analysis: Density plots of CD19^+^CD25^−/lo^ B cells (node 86 = CD19^+^CD25^−/lo^DAF-FM^−/lo or+/lo^, hazard ratio = 1.777, *p* = 0.034) from representative short and long RFS samples prior to ipilimumab treatment. **(C)** Wilc.1 analysis: Representative density plots of CD3^+^CD8^+^ effector T cells (node 155 = CD3^+^CD8^+^CD25^−/lo^DAF-FM^+^, RFS ≤ 1 year, mean = −8.666, SD = 0.694; RFS > 1 year, mean = −8.066, SD = 0.956, *p* = 0.035) at the pre-treatment stage from pateints with short and long RFS. **(D)** Cox.2 analysis: Naïve or memory CD8^+^ T cells (node 53 = CD3^+^CD8^+^CD127^+/lo^DAF-FM^+^; hazard ratio = 2.787, *p* = 0.047) displayed from both RFS group post ipilimumab treatment. **(E)** Wilc.2 analysis: Density plots of CD8^+^ naïve or memory T-cell subset (node 150 = CD3^+^CD8^+^CD127^+or+/lo^CD25^−/lo^DAF-FM^+^, RFS ≤ 1 year, mean = −6.979, SD = 0.583; RFS > 1 year, mean = −7.421, SD = 0.577, *p* = 0.037) from patient samples post treatment. **(F)** Wilc.3 analysis: A subset of regulatory T cells (node 159 = CD3^+^CD4^+^CD127^−/lo^CD25^+^DAF-FM^+^, mean = −0.325, *p* = 0.029) demonstrating a downward trend after treatment without any association with RFS. **(G)** Cox.4 analysis: Representative density plots of CD11c^+^ natural killer cells (node 42 = CD56^+/lo^CD11c^+^CD25^−/lo^DAF-FM^+^, hazard ratio = 1.659, *p* = 0.018) showing inverse trends from samples of short and long term RFS in reponse to ipilimumab treatment. **(H)** Wilc. 4 analysis subset of rare CD4 CD8 double negative T cells (node 185 = CD3^+^ CD127^+or+/lo^CD25^−/lo^DAF-FM^+^, RFS ≤ 1 year, median = 0.510; RFS > 1 year, median = −0.084, *p* = 0.041) increased after treatment in a subset of short-term RFS patients and decreased in the majority of long-term RFS patients.

**Figure 3:**
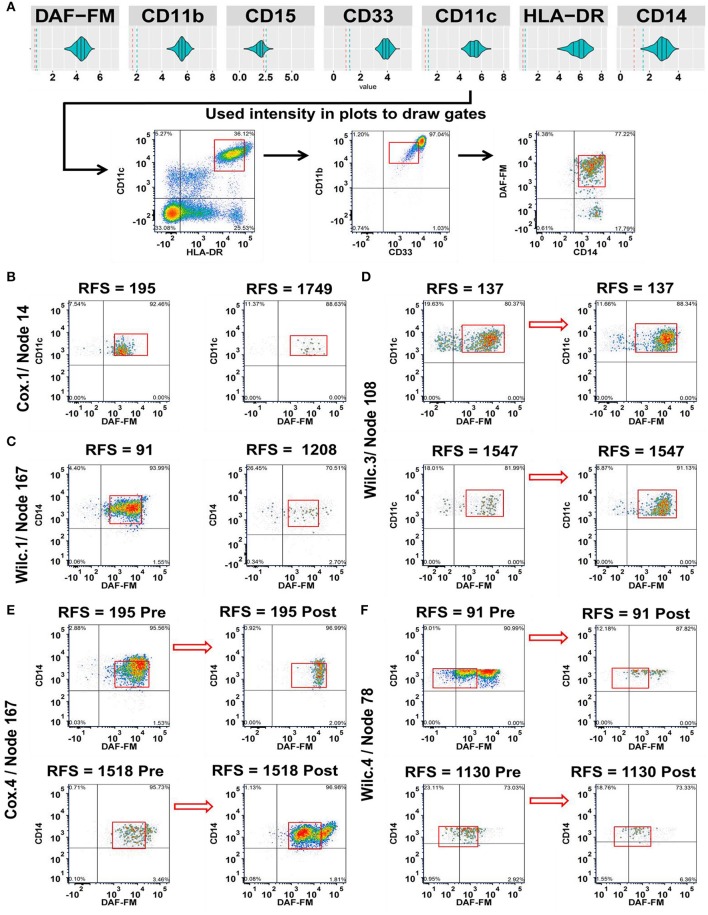
Integration of the MPATR output for the myeloid markers and delineation of cellular subsets associated with relapse-free survival (RFS) or treatment effects. **(A)** Unsupervised gating performed through the use of the myeloid marker median fluorescence intensity measurements generated from MPATR violin plots (as shown for node 167; mature monocytes) to delineate populations that were not easily seen within bivariate plots. **(B)** Cox.1 analysis: Representative density plots of a population of developing monocytes (node 14 = HLA-DR^+^CD33^−/lo or+/lo^CD11b^+^CD11c^+^ CD14^−/lo^DAF-FM^+^, hazard ratio = 3.592, *p* = 0.013) at pre-treatment stage of short and long term RFS samples. **(C)** Wilc.1 analysis: Mature monocytes that are CD33^+^CD14^+^ (node 167 = HLA-DR^+^CD33^+^CD11b^+^CD11c^+^CD14^+^DAF-FM^+^; RFS ≤ 1 year, mean = −8.394, SD = 1.168; RFS > 1 year, mean = −9.340, SD = 1.125; *p* = 0.028) analyzed at pre-treatment stage in both short and long RFS samples. **(D)** Wilc.3 analysis: Density plots of dendritic cells (node 108 = HLA-DR^+/lo^CD33^−/lo or+/lo^CD11b^+^CD11c^+^ DAF-FM^+^, mean = 0.370, *p* = 0.022) before and after ipilimumab treatment from representative short and long RFS samples. **(E)** Cox.4 analysis: A subset of mature monocytes (node 167 = HLA-DR^+^CD33^+^CD11b^+^CD11c^+^CD14^+^DAF-FM^+^; hazard ratio = 0.488, *p* = 0.021) were analyzed at both stages of treatment from short and long term RFS samples. **(F)** Wilc.4 analysis: Whereas, another subset of mature monocytes (node 78 = HLA-DR^+^CD33^+^CD11b^+^CD11c^+^CD14^+/lo^DAF-FM^+/lo^; RFS ≤ 1 year, median = −1.289), (RFS > 1 year, median = −0.243; *p* = 0.037) demonstrated a unique trend of NO expression at post ipilimumab therapy with samples of long RFS.

Nine lymphoid and 7 myeloid markers with and without the addition of scatter properties of the cells (forward scatter [FSC] and side scatter [SSC] areas) from the flow cytometry panels were used in the MPATR algorithm to delineate the phenotypes of specific cell populations. In the first step, the different phenotypes of cells were clustered using the SPADE algorithm, as shown by the SPADE trees generated from a representative PBMC sample (Figure 1A). The second step was to visualize the clustering in a user-friendly way, as ascertaining the phenotypes via traditional clustering analysis is time consuming. Violin plots were constructed with positive/negative cut-off lines for each node marker (cell phenotype) in patient samples. The MPATR application can display the violin plots (arcsinh transformation) either of each node (cell phenotype) for all the patient samples or of each patient sample (i.e., PBMC) for all the nodes (Figure 1B,). Alternate use of red and blue colors were utilized to distinguish the patient samples in case of “by Node” analysis. This is effective while scrolling through a large data set. The dotted red and blue lines denote 95 and 99% cut-offs for negative controls. In the negative controls, either 5 or 1% of the events are above this value, respectively. In addition, the application can scale the violin plots to the number of events in the node/sample (Figure S7). Each row is labeled by the node/sample number and the number of events in that node/sample in parenthesis (Figure 1B). The third step is to perform phenotype dimension reduction (Figure 1C). This process associates the number of events (cells) with each node (phenotype) to be used in downstream statistical analyses. MPATR provides the number of events per node in a summary table. This output was compiled into a “.csv” file compatible with excel that the user can visualize a table of columns (nodes) vs. rows (patient samples). In another column the outcome variable (RFS) was placed and facilitated statistical analysis. After this phenotype dimension reduction step, in which the multi-parameter flow cytometry stain is reduced to the number of events in a node for a particular sample, statistical analyses were used to determine which nodes were associated with RFS (Figure 1D). The visualization tool allows the user to quickly ascertain the phenotype using traditional flow cytometry software such as FCS Express. Nodes found in the statistical analyses were visualized using FCS Express 6, in which the fluorescence values were obtained from the violin plots and used for gating (Figures 2A, 3A).

### Delineation of the Role of NO Using the MPATR Algorithm

Four analyses were performed to determine which cell types associated with NO may contribute to the effect of adjuvant ipilimumab treatment: (1) pre-treatment nodes associated with RFS (continuous analysis:Cox Proportional Hazards, COX.1 or stratified by RFS > 1 year: Wilcoxon, Wilc.1), (2) post-treatment nodes associated with RFS (continuous analysis: COX.2 or stratified by RFS > 1 year: Wilc.2), (3) pre-treatment nodes that changed after treatment but were not required to be associated with RFS (Wilc.3), and (4) the number of events in a node that changed from pre-treatment to post-treatment that were associated with RFS (continuous analysis: COX.4 or stratified by RFS > 1 year: Wilc.4). The output was utilized as a score to determine which nodes were associated with RFS suitable for downstream analysis. In the discussion that follows, the traditional analyses are described, in which only the phenotypic markers are used for the clustering, in addition to the analyses that included the scatter properties of the cells (FSC and SSC) in the clustering tree.

After the preliminary analysis where all 200 nodes for each analysis (800 total for lymphoid and myeloid with/without FSC/SSC) were analyzed for phenotypes related to RFS in an unsupervised manner, all phenotypes with a *p* < 0.05 for each of the analyses were plotted in FCS Express for visualization purposes using batch techniques. As shown in Figures 2B,C, examples of relationships included: increases in the population of cells in melanoma patients prior to treatment with either decreased RFS (B cells without NO; node 86 = CD19^+^CD25^−/lo^DAF-FM^−/lo or+/lo^; Figure 2B) or increased RFS (Effector T cells; node 155 = CD3^+^CD8^+^CD25^−/lo^DAF-FM^+^), respectively (Figure 2C). A subset of CD8 naïve or memory T cells with moderate levels of NO (node 53 = CD3^+^CD8^+^CD127^+/lo^DAF-FM^+^) were found to be more prevalent in the short-term RFS samples post treatment (Figure 2D). A comparable T cell population (node 150 = CD3^+^CD8^+^CD127^+or+/lo^CD25^−/lo^DAF-FM^+^) reproduced similar trends (Figure 2E). Interestingly, there were changes in the number of immune cell subsets that were not associated with RFS. For example, A subset of regulatory T cells (node 159 = CD3^+^CD4^+^CD127^−/lo^CD25^+^DAF-FM^+^) with a low-to-intermediate NO level presented a relative downward trend after treatment without any association with RFS (Figure 2F). Lastly, we also observed examples where changes in the numbers of immune cells with therapy were associated with RFS. An example of this type of population are NK cells (node 42 = CD56^+/lo^CD11c^+^CD25^−/lo^DAF-FM^+^) with an intermediate level of NO. Patients with shorter RFS had increased numbers of NK cells in comparison to long-term samples after treatment (Figure 2G). A similar trend was also noticed for a rare subset of CD4^−^ CD8^−^ T cells (node185 = CD3^+^ CD127^+or+/lo^CD25^−/lo^DAF-FM^+^; Figure 2H). The tables for all the phenotypes with the associated statistics for the clustering of lymphoid cells is found in Tables S3, S4 (FSC/SSC clustering).

Next, we delinated the cellular subsets of myeloid cells that were associated with RFS post ipilimumab treatment (Figure 3). Relationships included were, increased number of developing monocytes (node 14 = HLA-DR^+^CD33^−/lo or+/lo^CD11b^+^CD11c^+^ CD14^−/lo^DAF-FM^+^) characterized by the low expression of CD33 and CD14 with moderate levels of NO pre-treatment in patients with short RFS (Figure 3B). Similarly, mature monocytes (node 167 = HLA-DR^+^CD33^+^CD11b^+^CD11c^+^CD14^+^DAF-FM^+^) with a moderate NO expression were found in increased numbers pre-treatment among patients with RFS ≤ 1 year (Figure 3C). Dendritic cells (node 108 = HLA-DR^+/lo^CD33^−/lo or+/lo^CD11b^+^CD11c^+^DAF-FM^+^) with intermediate levels of NO increased overall with treatment and were not associated with RFS (Figure 3D). Monocytes (node 167 expressing CD14 have decrease in NO in short RFS patients and increase in NO in long RFS patients (Figure 3E). In cells of monocytic lineage expressing less CD14 (node 78 = HLA-DR^+^CD33^+^CD11b^+^CD11c^+^CD14^+/lo^DAF-FM^+/lo^) the difference in NO expression (post-pre) is greater in patients with shorter RFS (Figure 3F). The phenotypes found in the unsupervised analysis are presented for the myeloid nodes [Tables S5, S6 (FSC/SSC)]. A schema for the levels of NO found in the various clinically relevant cell subsets is presented in Figure S8A. All the cell types found in this analysis are illustrated in Figure S8B. There are also cell types that the change in numbers may be associated with treatment but not associated with changes in RFS (e.g., Tregs; Figure S8C).

**Figure 4:**
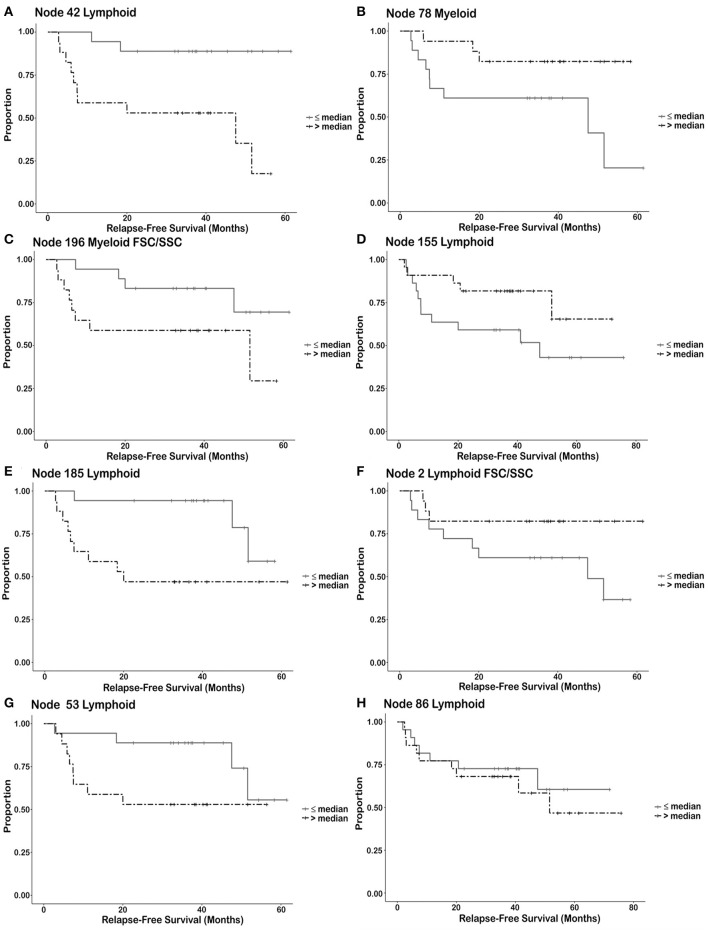
Kaplan Meier survival curves illustrating types of relationships between immune cell subsets and relapse free survival. Each node is divided into two groups or strata. A node from a particular patient is placed in the strata having greater than the median number of events for the population (all trial patients) or is placed in the strata having less than or equal to the number of events in the entire population for the node. Once these individual cell events are placed into these two groups, Kaplan Meier plots describe each group as a function of relapse free survival. (strata = ≤ median number of events/node, >median number of events/node). Discussion of these different groups can be found in results. **(A,B)** Utilization of the number of immune cells as a cut-off for predicting response. **(A)** Node 42 Lymphoid–NK cells (post-pre). **(B)** Node 78 Myeloid–Monocytes (post-pre). **(C,D)** Number of immune cells known to be associated with biology but may not be useful as a biological cut-off without additional information. **(C)** Node 196 Myeloid FSC/SSC–MDSC (post-pre). **(D)** Node 155 Lymphoid–Effector T cells (pre). **(E,F)** The survival curves separate after a prolonged period of time. **(E)** Node 185 Lymphoid–αβ T cells (post-pre). **(F)** Node 2 Lymphoid FSC/SSC–Tregs (post-pre). **(G)** The survival curves merge after a prolonged period of time indicating that relapse may be prolonged in these patients [Node 53 Lymphoid–CD8 memory/naïve T cells (post)]. **(H)** The survival curves show no difference, but there is a difference in the continuous variable (non-dichotomized) that may necessitate a different cut-off value for biomarker determination [Node 86 Lymphoid–B Lymphocytes (pre)].

Categories of nodes that were not studied further included the inability to visually discern the difference between responders and non-responders from the flow plots and nodes that contained less than a maximum of ~200 cells. Addition of FSC/SSC to the analysis did reveal additional nodes associated with RFS. Cell subsets such as myeloid-derived suppressor-like cells (MDSC-like) and Tregs associated with RFS were delineated in the FSC/SSC clustering analysis that clusters on size and granularity of the cells (Node 196 and 2, respectively). The resulting immune cell phenotypes associated with RFS may be split into four categories based upon the RFS characteristics for each node. Each node is divided into two groups (strata). A phenotype (node) from a particular patient is placed in the strata having greater than the median number of events for the population (all trial patients) or is placed in the strata having less than or equal to the number of events in the entire population for the node. Once these individual cell events are placed into these two groups, Kaplan Meier plots describe each group as a function of relapse free survival. This analysis is done to explore which phenotypes may be useful as predictive biomarkers in future studies. First, there may be a cut-off that can be utilized for potential biomarkers (Node 42–NK cells positive for DAF-FM, Figure 4A and Node 78–Monocytic cells positive/low for DAF-FM, Figure 4B). NK cells positive for NO are associated with increased RFS. Monocytic cells positive or low for nitric oxide are associated with increased RFS but the magnitude of the effect appears to be less than for the NK cell population. Second, the variables (# of events in a node) is continuous such that the immune cell phenotype present may be indicative of biology but may not be useful for exact cut-off biomarkers (Node 196 FSC/SSC–MDSC-like cells positive for DAF-FM, Figure 4C; Node 155–Effector T cell positive for DAF-FM, Figure 4D). Increased numbers of MDSC staining positive for NO are associated with decreased RFS whereas increased numbers of Effector T cells positive for NO are associated with increased RFS. A third category is when the Kaplan Meier curves separate after a certain period of time, or a group of patients appear to derive long term benefit (>2 years). Phenotypes in this category include Node 185–CD4^−^CD8^−^ αβ T cells positive for DAF-FM (Figure 4E), node 2 FSC/SSC–Treg negative/low for DAF-FM (Figure 4F) and node 53–CD8 naïve/memory T cells positive for DAF-FM (Figure 4G). Immature αβ T cells are associated with decreased RFS whereas this one Treg population may be associated with increased RFS. A fourth category includes cell phenotypes (nodes) that were associated with RFS with a continuous variable (# events/node) but the survival curves did not demonstrate a difference in RFS (Node 86–B lymphocytes with negative or very low levels of DAF-FM found prior to therapy, Figure 4H). In general, effector cells with higher levels of NO appear to be associated with increased RFS, whereas suppressor cells associated with higher NO and likely increased NO expression levels with treatment appear to be associated with decreased RFS.

A subset of seven samples pre and post treatment (4/RFS > 1,000 days, and 3/RFS <365) were analyzed to investigate the immunostimulatory/inhibitory markers on these cells other than NO found within specific nodes. Consistent with the literature, NK cell, αβ T cells both express CD3ζ. Whereas, CD4^+^ and CD8^+^ memory T cells express CD69 (Figure S9). NK cells also express CD3ζ and lower levels of CD69 (Figure S10). In a Treg population that had decreased levels of NO after treatment TCRζ also decreased in expression (Figure S11). Myeloid subsets such as MDSC and DCs exhibited decreased Arginase 1 with treatment in patients with long RFS (Figure S12). PD-L1 was found on the surface of dendritic cells in a patient that had poor RFS (Figure S13). In mature monocytes, PD-L1 levels minimally increase pre/post treatment, but NO levels increased after treatment in patients with long RFS (Figures S14A–C). Higher NO levels pre-treatment were associated with poor RFS and no subsequent changes with therapy in either Arginase 1 or PD-L1 (Figures S14D–F).

The MPATR approach was extremely useful to assess the role of NO by each individual phenotype (node) and to have the ability to superimpose new samples onto an existing clustering tree (Figure S5). To assess the immune populations as a whole and to investigative how NO is associated with RFS, we utilized PCA and PLS models (18). Robust PCA models were identified for the samples collected prior to therapy and for a post-pre set of lymphoid nodes (Figures S15, S16).

Detailed description of nodes associated with PCA analysis were tabulated in Tables S7A–D. The PLS model was utilized to associate the linear combination of these principal components associated with good overall survival and robust models were identified for pre and post data sets (Figures S17, S18). In these models, NO prior to therapy was associated with cells that can manifest a NO burst responsible for tumor control (i.e., effector T cells), but NO in inhibitory cell types or persistent NO stimulation is associated with worse prognosis (Figures S15–S18) as is also shown in Figure 4. Description of nodes associated with PLS models tabulated in Tables S8A–D. The classical thinking that NO merely serves an inhibitory role in melanoma needs to take into account the role of NO in eliminating foreign cells (e.g. bacteria) in the immune response as is found in the classical infectious disease literature (19, 20).

## Discussion

Clustering analyses were performed to identify the pro- and anti-tumor activities of NO in patients undergoing ipilimumab therapy. Multiple clustering programs are available that allow visualization of clustering and the ability to cluster across publically available databases (21–24). The MPATR pipeline approach grants the user the ability to cluster, visualize specific cellular subsets across patient populations in an easy-to-use interface (violin plots and data matrix output), and to superimpose new samples onto existing clustering analyses. All of these items together are not available via available current clustering or visualization algorithms, such as CCAST, Citrus, tSNE, and viSNE, Cytobank, Cytosplore, and Immport-Galaxy (21–28).

The generation of violin plots as PDFs for each node permits the unsupervised gates to be plotted in flow cytometry analysis software such as FCS Express 6. As an example, in this study, rare phenotypes such as a subset of CD8^+^ NK cells (Table S4, FSC/SSC node 117) and CD4^−^CD8^−^ αβ T cells (Table S3, nodes 185 and 36) were easily found in an unsupervised manner (29, 30). In addition, MPATR's “dimension reduction” summary table output provides a quantitative construct of relative node density and allows the user to troubleshoot the data by finding batch effects and examining file integrity of the flow cytometry files for immunologists and physician scientists. Mathematical properties of the clustering results in either truncating zeros or nodes with extremely high values. Although identification of specific issues must be performed by those experienced with the relevant techniques, MPATR allows immunologists and physician scientists to determine whether there is an issue with their dataset (truncation of data and inappropriate compensation matrix applied to the data), prompting them to obtain advice if needed. The MPATR method was used in the current study to analyze the distribution of NO in immune cell subsets obtained from patients undergoing adjuvant ipilimumab therapy.

Even though NO has been classically associated with aggressive melanoma growth, NO can show both pro- and anti-tumor effects in a concentration- and context-dependent manner (9, 31–34). For instance, suppression of T cells by MDSC was found to be dependent on the MDSC's NO content (9, 35). We have recently reported that MDSC-produced NO can interfere in the cancer cell antigen presentation from DCs to T cells via the Jak-STAT pathway (36). On the other hand, NO production may also serve macrophage-mediated control of melanoma tumors (9, 37, 38) and plays a vital role in the regulation of T-cell functions, their differentiation, and cell death (39). In our studies, we have observed different levels of NO in a wide variety of immune cells that are associated with increased or decreased RFS, depending on cell type. For instance, CD8^+^ NK cells and monocytes positive for DAF-FM staining were associated with anti-tumor activities at either pre-treatment or post-treatment stages. The numbers of effector T cells as a group changed with therapy but were not associated with RFS. However, as demonstrated in Figure 2C (lymphoid node 155) there was one subset of effector T cells associated with RFS. Levels of TCR-ζ were increased in effector T cells with increased RFS (Figure S9) consistent with the literature that downregulation of the TCR-ζ is associated with immune escape (40). CD69^+^CD8^+^ memory T cells generated before melanoma inoculation play a critical role in tumor surveilance (41). Whereas in the late phase of an immune response, depletion of CD69 levels in CD4 T cells resulted in reduced production of high-affinity antibodies and long-lived plasma cells in bone marrow (42). CD69^+^ memory CD8^+^ and CD4^+^ T cells were found in PBMC populations analyzed from patients with long RFS (>1,000 days) prior to and after therapy, respectively (Figure S9). On the other hand, intermediate levels of NO in B cells, double-negative αβ T cells, CD8^+^ naïve or memory T cells, MDSCs, immature monocytes, and DCs were pro-tumor in nature and associated with short-term RFS.

Another class of immune cells that have had contradictory reports as to whether they correlate with ipilimumab efficacy are Tregs (43–47). More recently, depletion of Tregs was found to be important in CD8^+^ T-cell-inflamed tumors (48). In addition, other recent studies have demonstrated overall decreases in Tregs after ipilimumab treatment, but there is significant overlap between the 2 groups (49). We identified 2 different Treg subsets by employing our automated phenotyping algorithm MPATR: one with a moderate NO (lymphoid node 159) level and the other one with a low/negative NO level (node 2 FSC/SSC lymphoid). The moderate NO population changed after treatment, yet no correlation with response was shown. In contrast, the small subset of Tregs with low/negative NO levels were associated with longer term RFS after 2 years. This population of CD3^+^CD4^+^CD25^+^CD127^neg/low^ was confirmed to be FOXP3^+^ Tregs by flow cytometry in a subset of 14 samples (Figure S11, Table S9). Thus, by employing the MPATR algorithm with DAF-FM as an additional marker, we were able to identify a dichotomy between the two distinct Treg subsets in regards to how their NO levels correlated with RFS. Similarly, attempts have been made to evaluate the biomarker potential of Teff, yielding no conclusive results to date (2, 49, 50). Using MPATR, we found one node of Teff cells with intermediate levels of NO are associated with increased RFS (lymphoid node 155), whereas Teff with low/negative NO level (lymphoid node 107) that had changed following treatment did not show any association with RFS. Interestingly, recent studies have demonstrated the importance of memory and NK cells in high-dimensional analysis, but the numbers overlapped between responders and non-responders (26). In this study, NK cells were associated with increased RFS, but NK subsets were also found that have no such relationship (Table S3). Chronic stimulation of NK cells is known to cause increased expression of stimulatory markers such as CD69 with decreased NK cell activity (Figure S10) (51). We also found evidence NK cells with elevated DAF-FM, CD69, and TCR-ζ in a PBMC sample with poor RFS (<1 year; Figure S10).

As expected, we found that B cells associated with response have low levels of NO. It is possible that they may be serving a regulatory role, even though they do not express high levels of CD25 (52).

The MPATR algorithm also demonstrated a similar dichotomy in the role of NO for myeloid cells. Traditionally, monocytic accumulation in the tumor and blood has been associated with decreased survival (53–55). More recently, peripheral blood monocyte levels have been found to overlap between responders and non-responders in stage IV melanoma patients (56). In our studies we also saw this overlap, but levels of NO may distinguish different cell populations (Figure S8A). For instance, monocytes with negative or low levels of NO (myeloid FSC/SSC node 185) were only associated with treatment changes, whereas monocytes with intermediate levels of NO (myeloid node 78) were associated with increased RFS. It was recently described via a high dimensional mass cytometry screen that CD14^+^HLA-DR^+^ monocytes are associated with improved overall survival to anti-PD1 immunotherapy (57). Levels of nitric oxide has the potential to identify those patients with high monocyte counts and poor response to therapy.

Lastly, MDSCs are immature myeloid cells that function to inhibit other immune cells, such as lymphoid cell populations (58). MDSC have a wide range of phenotypes but are known to be HLADR^neg/low^, CD33^neg/low/+^ depending on level of maturity but there is still ongoing research as to how to fully characterize these cells (59–61). Interestingly, cells with the MDSC phenotype were found in a distinct node when we also clustered on the size and granularity of the cells. Treatment with ipilimumab has been shown to decrease a subset of MDSC (monocytic) and increase CD8 memory T cells (49). In our studies, MDSC followed this trend of increasing in patients who had shorter-term RFS. Interestingly, the MDSC populations were easily found when FSC and SSC were taken into account by the clustering algorithm. Post treatment reduction of myeloid Arginase 1 levels association with long RFS (Figure S12) is consistent with the long held belief that increased Arginase 1 and elevated NO levels contributes to cancer immune escape (61–63).

In summary, this paper has two major conclusions. First, MPATR is a method that can be used by physician scientists and translational immunologists to profile phenotypes among immune cell populations. The MPATR pipeline is the first major step toward moving these methods into the hands of the general immunology community. The primary purpose of this method in the current form is to facilitate correlative studies for immune based therapies in clinical trials. For the first time we present a graphical user interface for analyzing flow cytometry data that was designed by a physician scientist specifically for use in analyzing correlative data from clinical trials. The user of these methods must still be mindful of issues such as overfitting and reduced signal-to-noise ratio for nodes that have minimal cell events. Second, and more importantly for the current audience, we applied the MPATR pipeline to a set of samples derived from patients undergoing anti-CTLA-4 adjuvant therapy. In doing so we observed that effector cells of the immune system with elevated levels of NO may be beneficial for long relapse-free-survival whereas NO production by suppressor cells of NO may be deliterious for relapse-free survival. As with any analysis of this type, this is the first step and we plan to validate these findings in the future in additional clinical specimens. The same type of analysis may be performed on other patient datasets to decipher the immune cell milieu of both the peripheral blood and, potentially, also the tumor microenvironment. We believe that the approach to the analysis, which has revealed trends demonstrating the dichotomy of NO associated with pro-/anti-tumor effects to immune-based therapy, is relevant to the translational medicine community at large and may be readily applied to clinical trials by allowing for efficient unsupervised organization of immune cell phenotypes.

## Data Availability Statement

The datasets generated for this study are available upon reasonable request to the corresponding author.

## Ethics Statement

The studies involving human participants were reviewed and approved by Scientific Review Committee Moffitt Cancer Center, USF-IRB deemed the research non-human subjects research as the samples were de-identified prior to this endeavor. The patients signed consent for trial for exploratory immune experiments. Written informed consent for participation was not required for this study in accordance with the national legislation and the institutional requirements.

## Author Contributions

SG, MO, AGMM, AWM, AA, and JM participated in the designing and/or interpretation of the reported experiments or results and participated in the acquisition and/or analysis of data. YC, AB, JJM, and JM provided administrative, technical, or supervisory support. YC, BC, and BS were responsible for the statistical analysis. JM and JK designed the flow cytometry panels. JM designed the algorithm and with help from ZC and AB implemented it in R. All authors participated in revising the manuscript.

### Conflict of Interest

Moffitt Cancer Center filed a provisional patent based on the MPATR algorithm. Institutional grants (JM) that are not a conflict of interest for this paper were received from Morphogenesis, Navigate BP, and Jackson Laboratories. JM is on the data safety monitoring committee for NewLink Genetics and is not a conflict for this paper. Clinic trials (JM) were sponsored with support unrelated to the current study given to the institution by REATA Pharmaceuticals, Idera pharmaceuticals, Morphogenesis Inc., Macrogenics Inc., and Merck. The remaining authors declare that the research was conducted in the absence of any commercial or financial relationships that could be construed as a potential conflict of interest.
