# Supplementary material for: Multi-Dimensional Flow Cytometry Analyses Reveal a Dichotomous Role for Nitric Oxide in Melanoma Patients Receiving Immunotherapy
Source: Front Immunol. 2020 Feb 25;11:164. doi: 10.3389/fimmu.2020.00164 (PMC7052497; doi:10.3389/fimmu.2020.00164)
Supplement: Supplementary file 1 [file Data_Sheet_1.pdf]

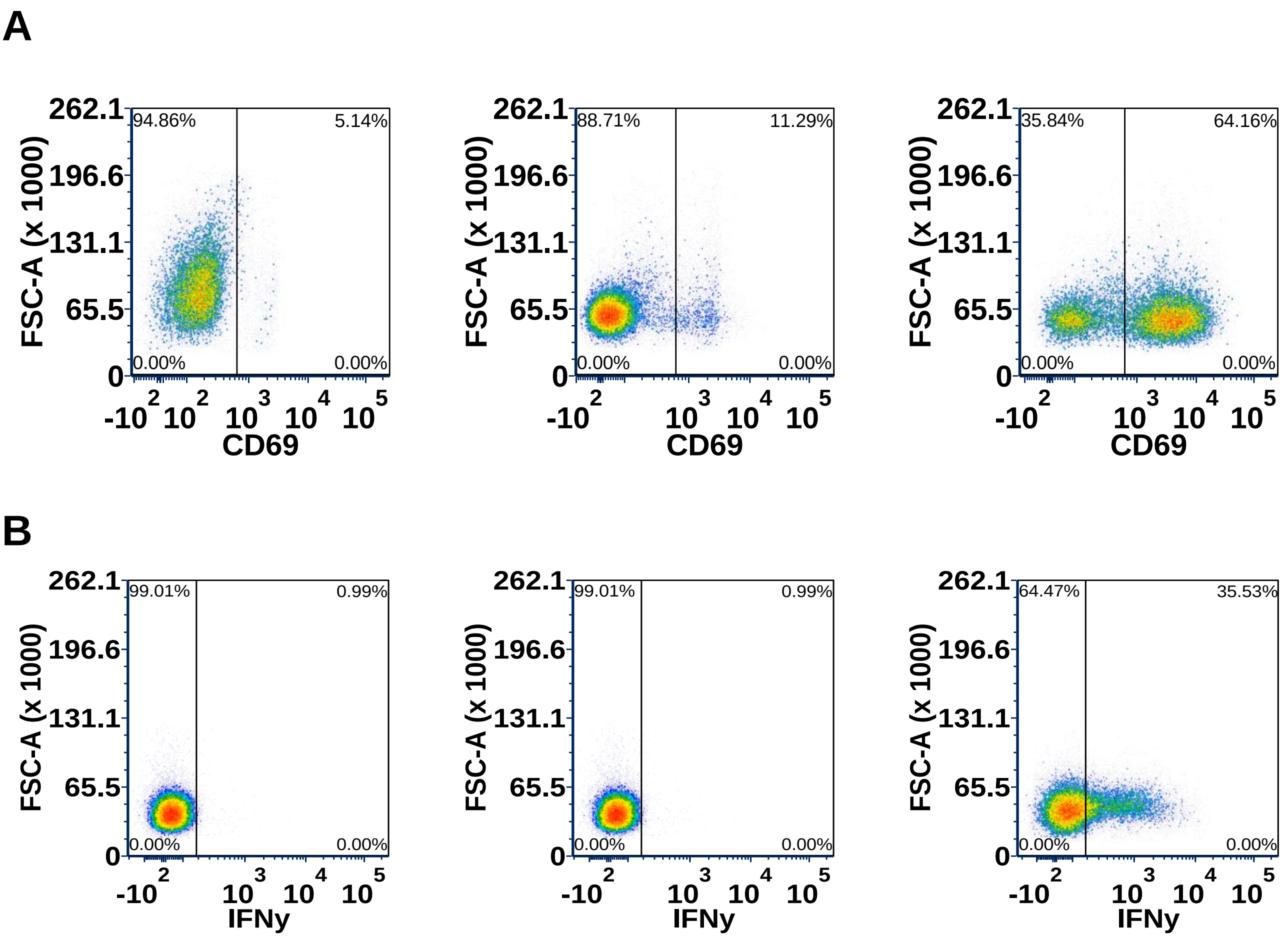

Fig. S1. Positive control for CD69 and IFN-γ. (A) CD69 unstained (left) live human PBMCs. Stained (middle) with anti-CD69-APC-R700, unstimulated PBMCs and (right) stained with anti-CD69-APC-R700 after 72 hrs. stimulation with PHA (5 µg/ml). (B) IFN-γ (left) unstained live human PBMCs. Stained (middle) with anti-IFN-γ-BV786, unstimulated PBMCs and (right) stained with anti-IFN-γ-BV786 after 24 hrs. stimulation with PMA (50 ng/ml) and Ionomycin (1 µg/ml).

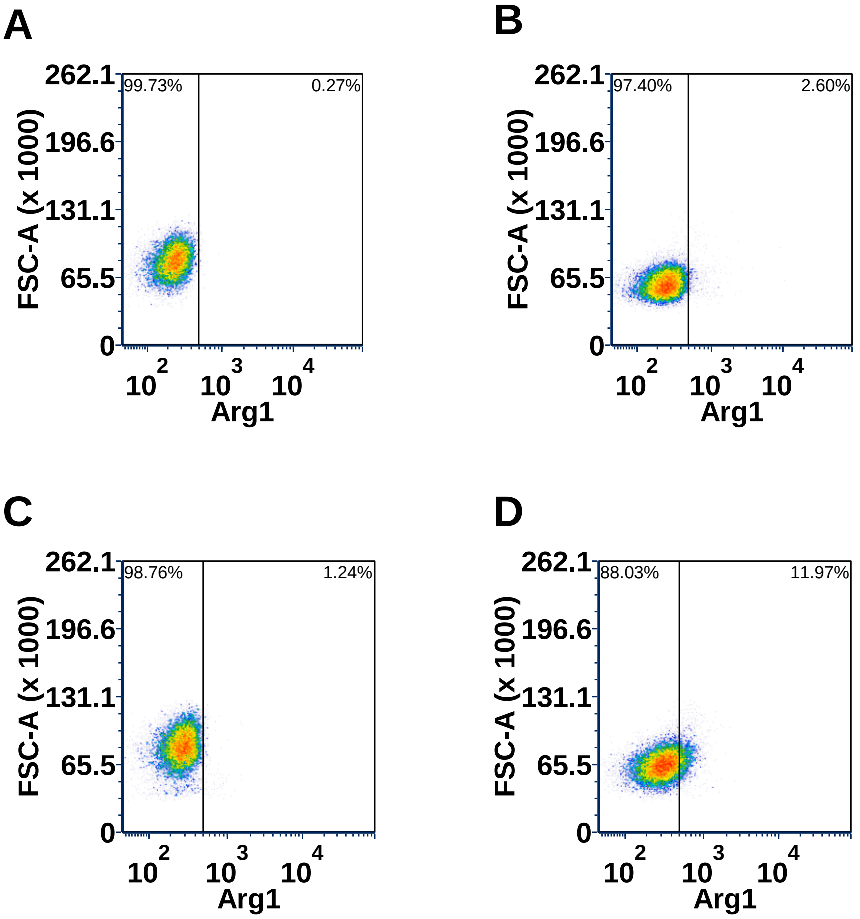

Fig. S2. Positive control for Arginase 1. (A) Unstained, unstimulated live human PBMCs. (B) Stained with anti-Arg1-PE, unstimulated PBMCs (C) Unstained, stimulated PBMCs. Stimulation with PHA (5 µg/ml) for 48 hrs. (D) Stained with anti-Arg1-PE, stimulated PBMCs.

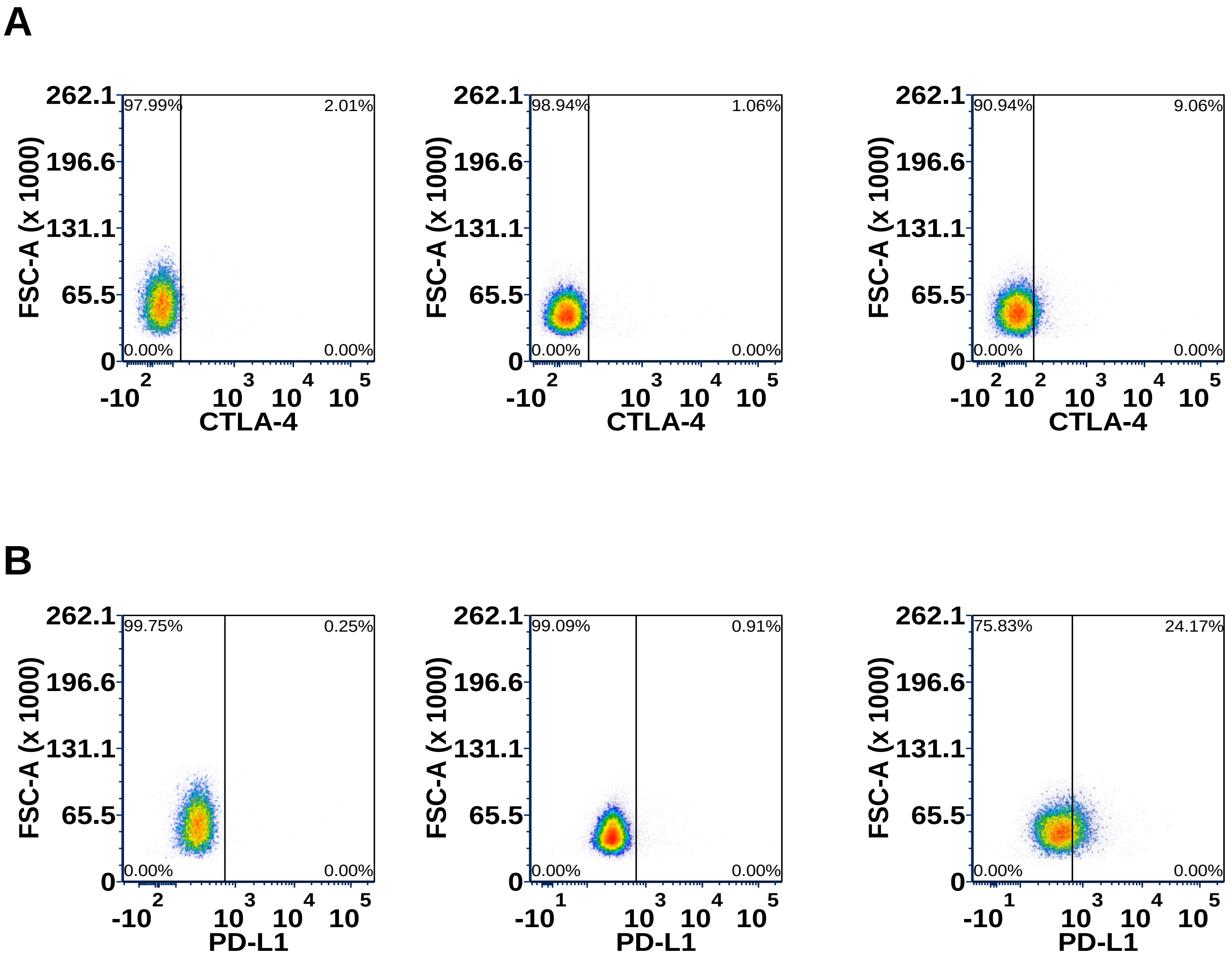

Fig. S3. Positive control for CTLA-4 and PD-L1. (A) CTLA4 (left) unstained live human PBMCs. Stained (middle) with anti-CTLA4-BV421, unstimulated PBMCs and (right) stained with anti-CTLA4-BV421 after 72 hrs. stimulation with PHA (5 µg/ml). (B) PD-L1 (left) unstained live human PBMCs. Stained (middle) with anti-PD-L1-BB515, unstimulated PBMCs and (right) stained with anti-PD-L1-BB515 after 72 hrs. stimulation with PHA (5 µg/ml).

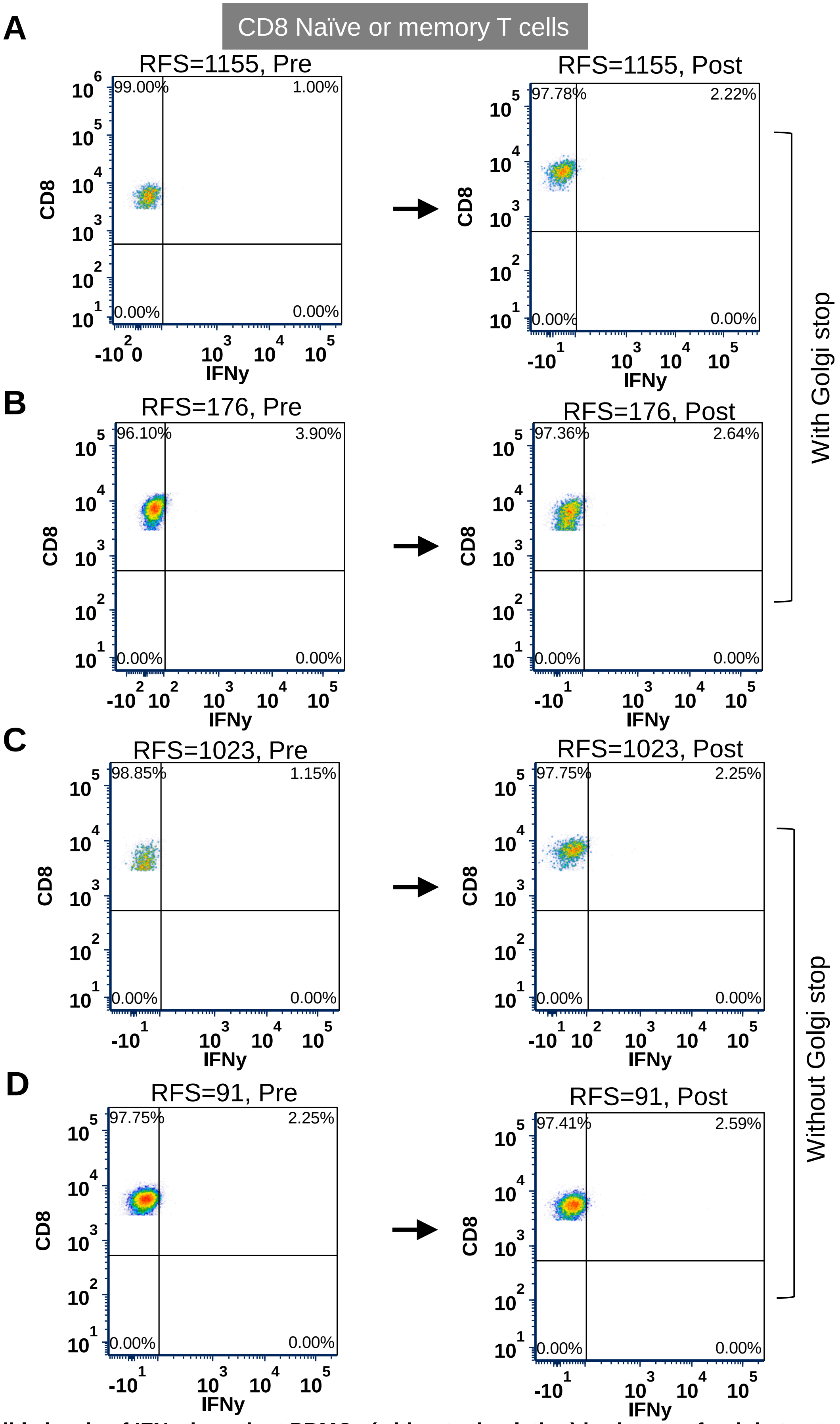

Fig. S4. Negligible levels of IFN-γ in patient PBMCs (without stimulation) irrelevant of golgi-stop treatment. (A-B) illustrating pre and post levels of IFN-γ from samples derived from patients with short and long RFS, respectively after PBMCs were processed with golgi-stop method. (C-D) illustrating pre and post IFN-γ levels from samples derived from patients with short and long RFS, respectively after PBMCs were processed without the golgi-stop method.

A

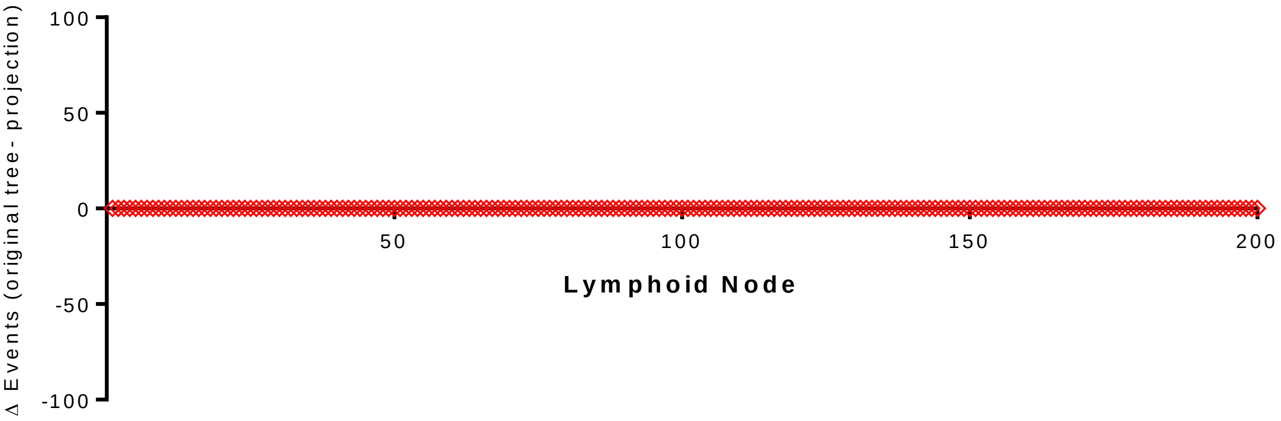

B

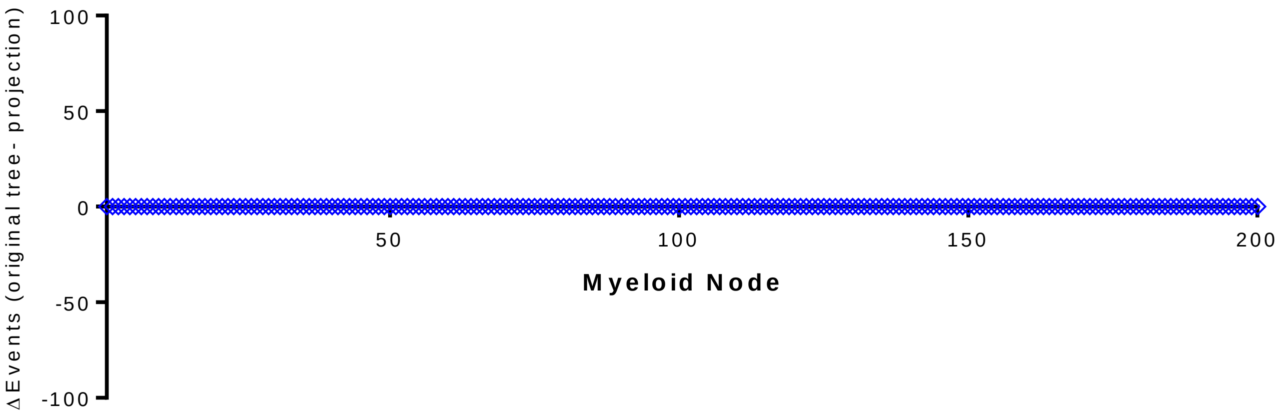

Fig. S5. Projection of all 200 nodes on corresponding tree. Differences in the number of events between the nodes of the original tree and the projections were calculated for the (A) Lymphoid, (B) Myeloid populations. Both the graphs indicate a perfect match between the number of events in the original nodes and the nodes calculated by the projection.

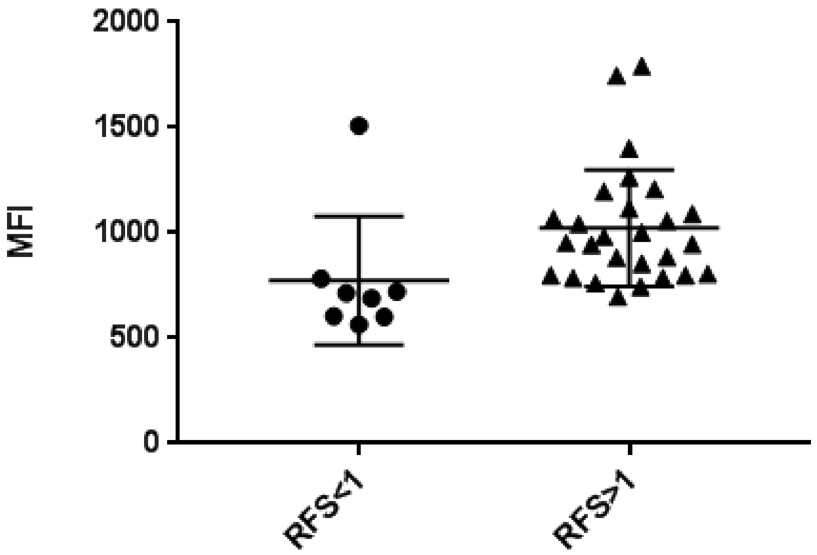

Fig. S6. Phospho-STAT1 expression in patients with a RFS less than one year and with a RFS greater than one year. RFS > 1 (Mean = 1022.45, SD = 277.05); RFS ≤ 1yr (Mean = 773.12, SD = 306.18); p = 0.001.

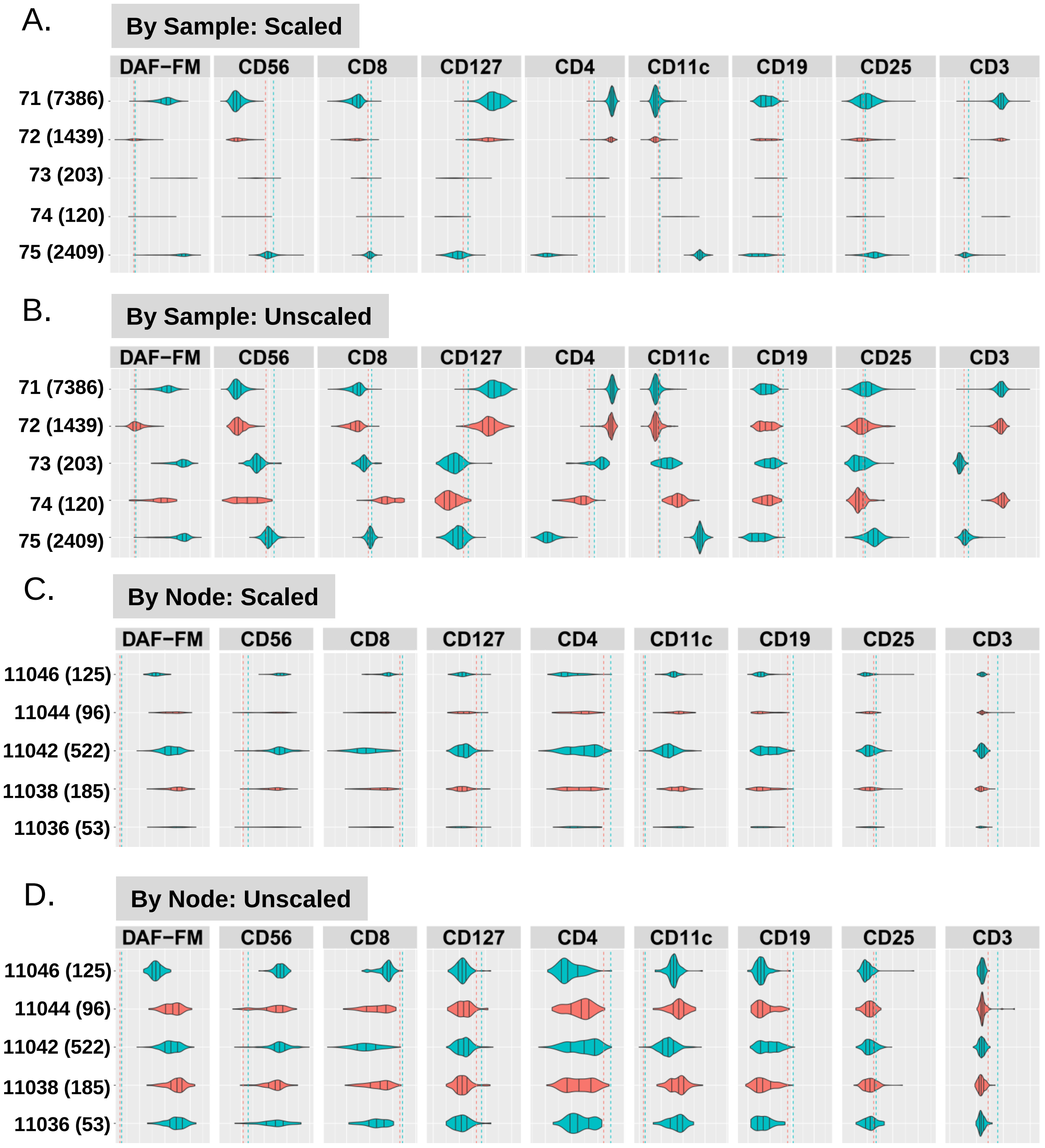

Fig. S7. Visualization of violin plots. (A-B) Displaying violin plots for de-identified PBMC sample: 10949 (Pre; RFS=1548) for lymphoid nodes 71 to 75. (A) Scaled for the number of events. (B) Unscaled. (C-D) Displaying violin plots for lymphoid node number 42 (NK cells) for de-identified PBMC samples: 11036 to 11046. (C) Scaled for the number of events. (D) Unscaled. Color scheme and positive/negative cutoff was as described in figure1. Alternate colors are utilized to facilitate visualization. Positive/negative cutoff was demonstrated by blue and red dotted lines (99 and 95% confidence intervals), respectively.

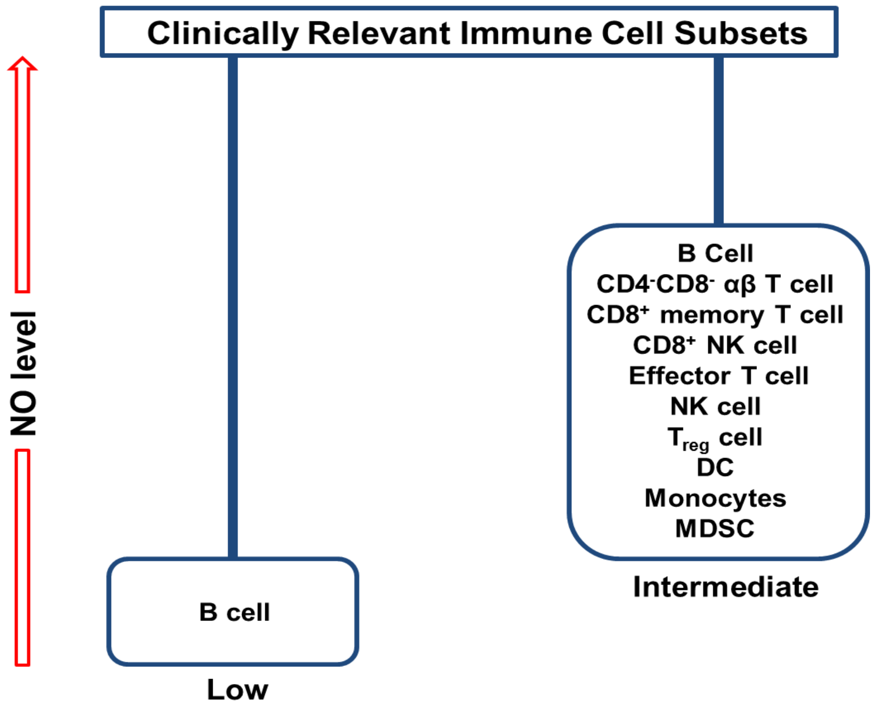

Fig. S8a. Association of immune cell subsets with RFS and NO levels. Cell subsets are presented with interesting clinical trends associated with low or intermediate NO levels. False discovery rates and Storey q-values were estimated for each subset of analyses to adjust for multiple comparisons but these analyses are overly stringent for this data set as there are not 200 independent cell types. **Abbreviations:** DC, dendritic cell; MDSC, myeloid-derived suppressor cell; NK, natural killer cell; T_{reg}, T-regulatory cell.

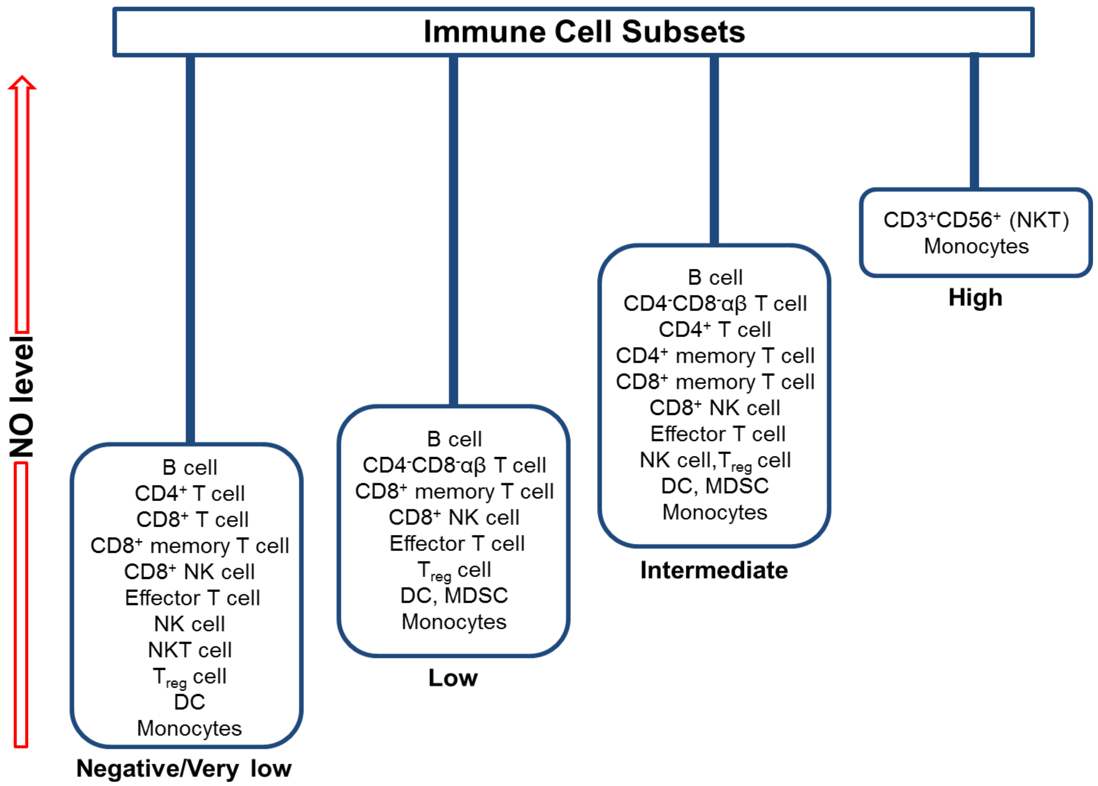

Fig. S8b. Nitric oxide levels in immune cell subsets associated with adjuvant ipilimumab and peptide vaccine treatment, identified by the MPATR algorithm.

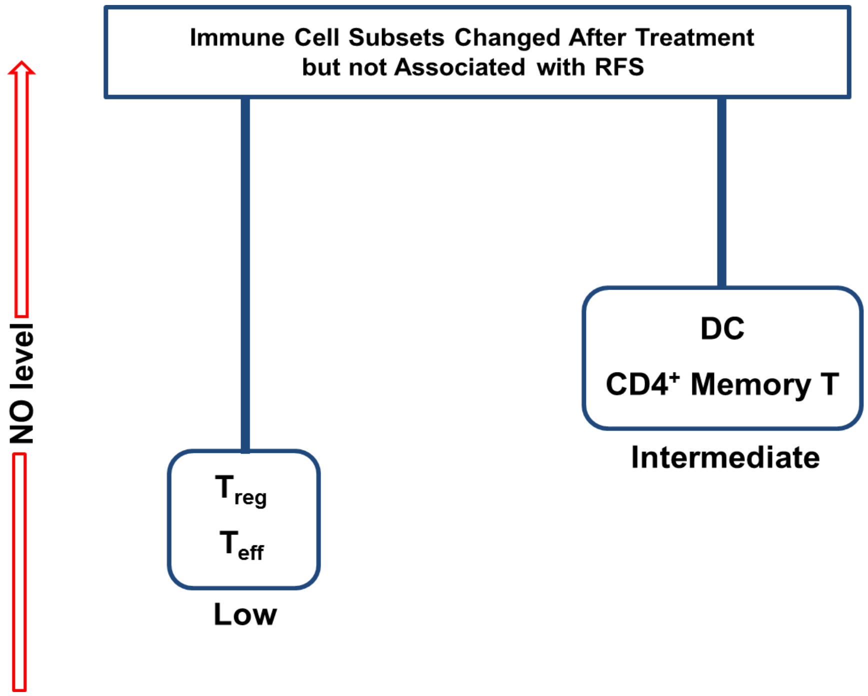

Fig. S8c. Nitric oxide levels in immune cell subsets that changed with adjuvant ipilimumab and peptide vaccine treatment but were not associated with RFS, identified by the MPATR algorithm.

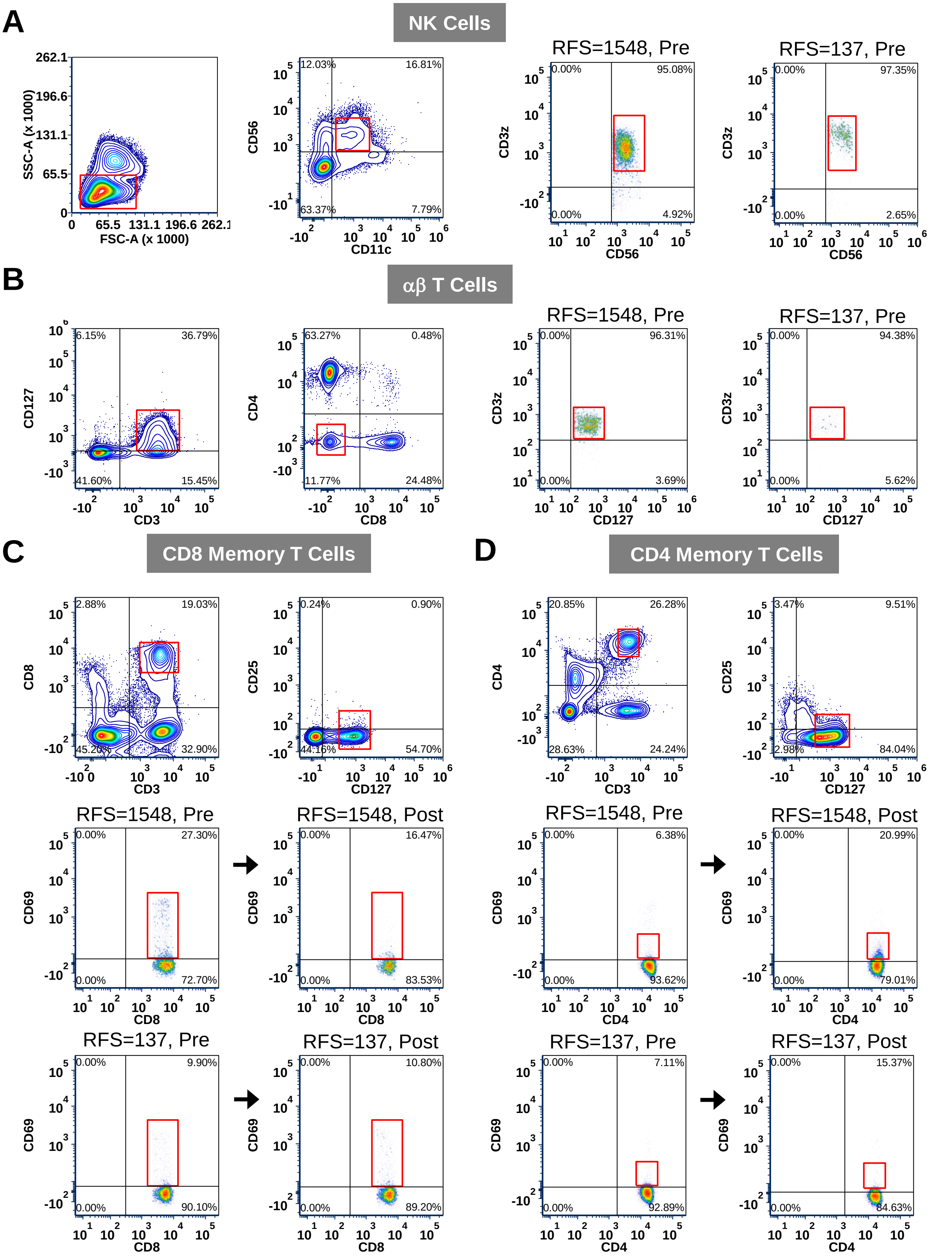

Fig. S9. Activation marker CD69 and TCR-ζ in NK, αβ T cells, CD8 and CD4 Memory T cells. (A) Numbers of NK cells (CD56^{+}CD11c^{+}CD25^{-}or$^{-/lo}$) with TCR-ζ expression increase pre-treatment in PBMCs collected from a patient with long RFS. (B) A similar trend was noticed for αβ T cells (CD3^{+}CD127$^{+/lo}$). (C) A subset of CD8 memory T cells (CD3^{+}CD8^{+}CD127$^{+\text{ or }+/lo}$CD25$^{-/lo}$) demonstrated CD69 expression in pre-treatment PBMC samples from patients with long RFS. (D) CD69 was elevated in small population of a subset of CD4 memory T cells (CD3^{+}CD4^{+}CD127^{+}CD25$^{-/lo}$) in post treatment PBMC samples with long RFS.

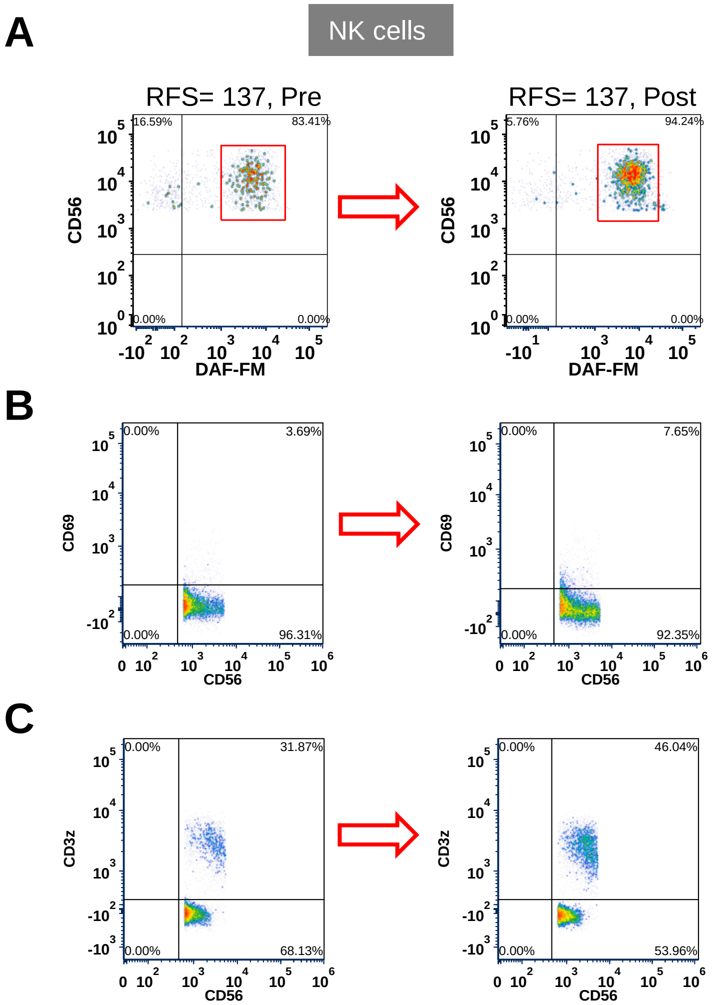

Fig. S10. Chronic stimulation of NK cells demonstrates positive staining of NO, CD69 and TCR-ζ in PBMC samples with poor RFS. (A) Natural killer cells (node 42 = $CD56^{+/lo}CD11c^{+}CD25^{-/lo}DAF\text{-}FM^{+}$) with an intermediate level of NO increased after treatment for most patients with short term RFS (B-C) CD69 and TCR-ζ also showed similar trends although CD69 demonstrated this trend in fewer cells.

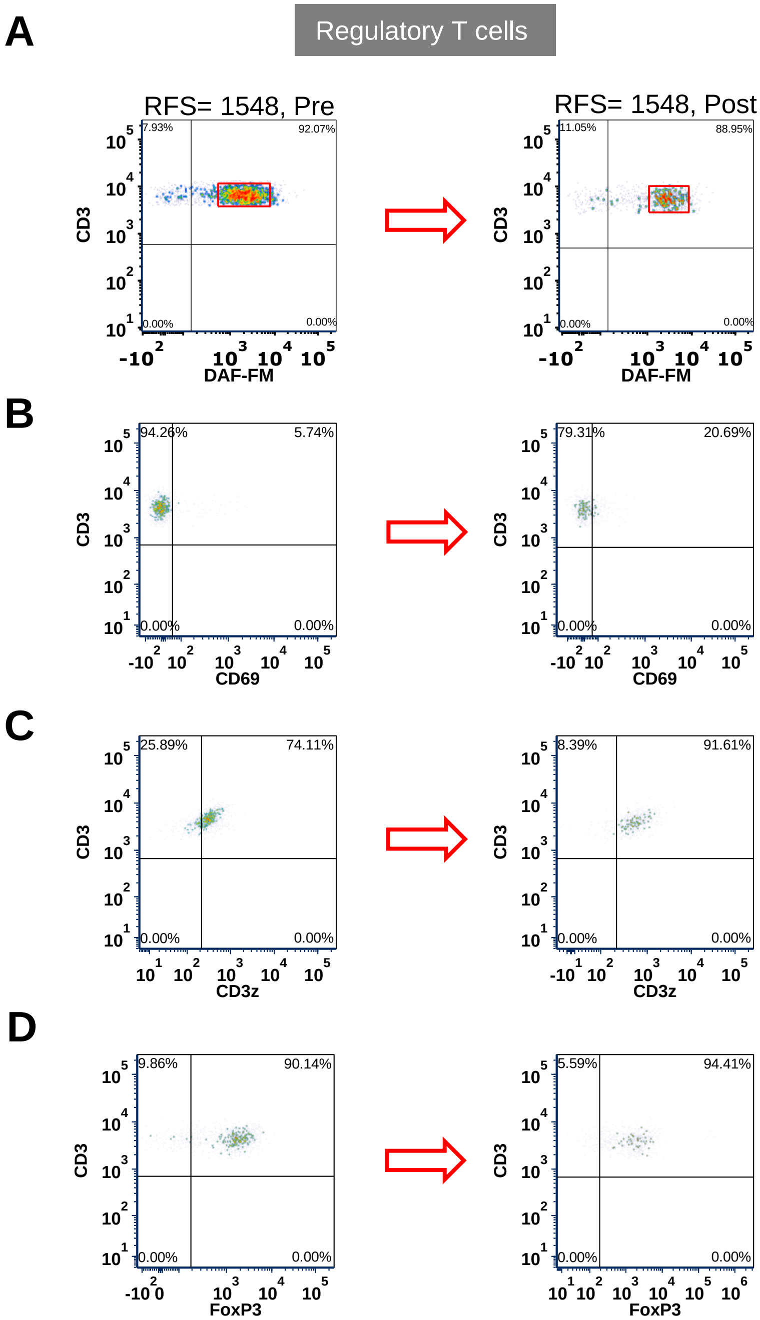

Fig. S11. Decreased NO and TCR-ζ expression in regulatory T cells in PBMC sample after treatment. (A) Numbers of regulatory T cells (node 159 = CD3$^+$CD4$^+$CD127$^{-/lo}$CD25$^+$DAF-FM$^+$) with a low-to-intermediate NO level decrease after therapy (B) CD69 was not expressed either pre/post therapy whereas (C) TCR-ζ$^+$ Tregs decreased after therapy. (D) FoxP3 expression was positive in 90% pre-treatment and 94% post-treatment in gated events.

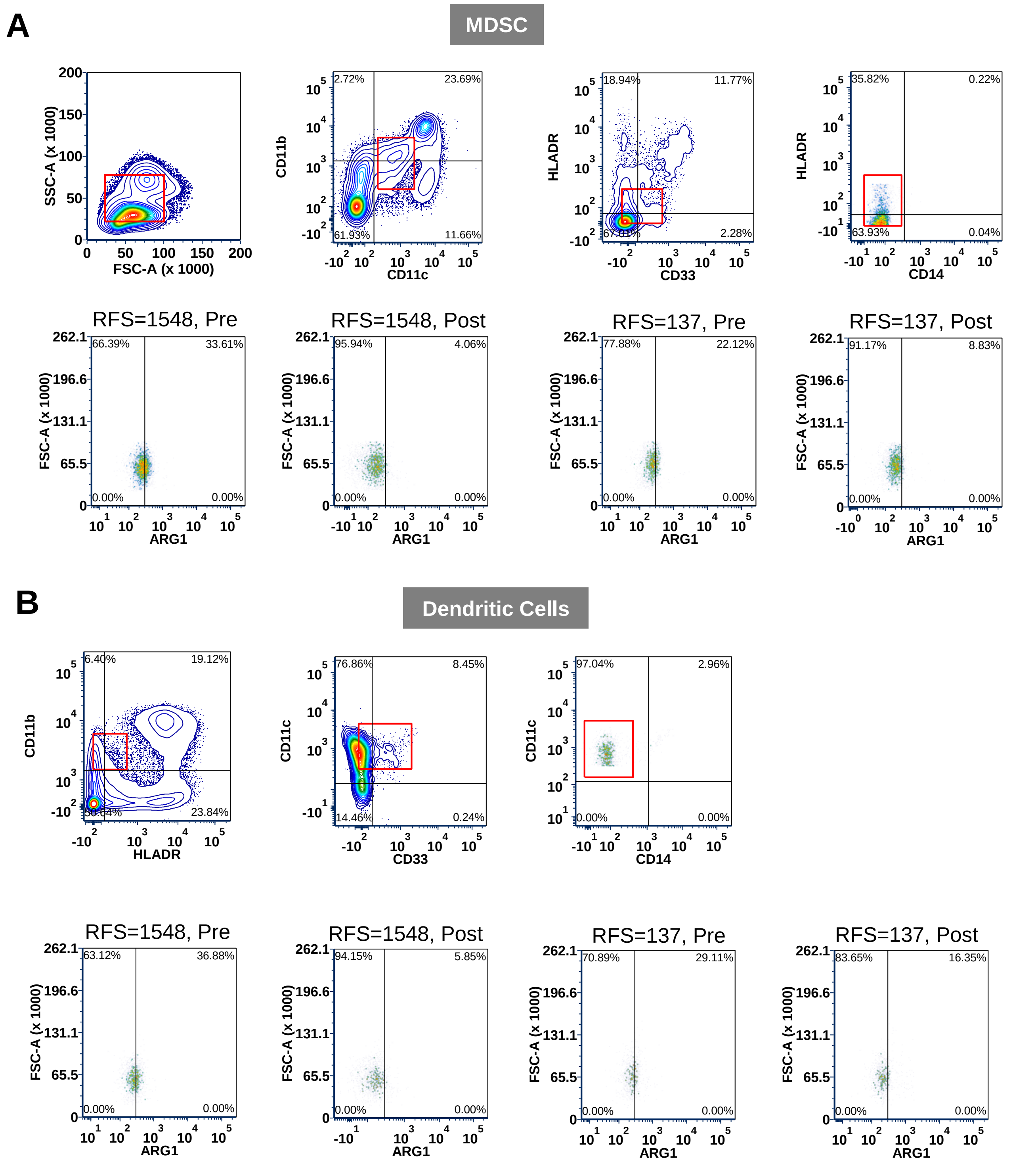

Fig. S12. Downward trend of Arginase 1 expression in MDSCs and dendritic cells is associated with long RFS. (A) MDSC-like (Node 196 FSC/SSC = HLADR$^{-/lo\ or\ +/lo}$CD33$^{-/lo\ or\ +/lo}$CD11b^{+}CD11c^{+}DAF-FM^{+}) and (B) DC (Node 108 = HLA-DR$^{+/lo}$CD33$^{-/lo\ or\ +/lo}$CD11b^{+}CD11c^{+}DAF-FM^{+}). MDSC and dendritic cells exhibited decreased levels of the immune inhibitory molecule Arginase 1 post-treatment in patients with long RFS.

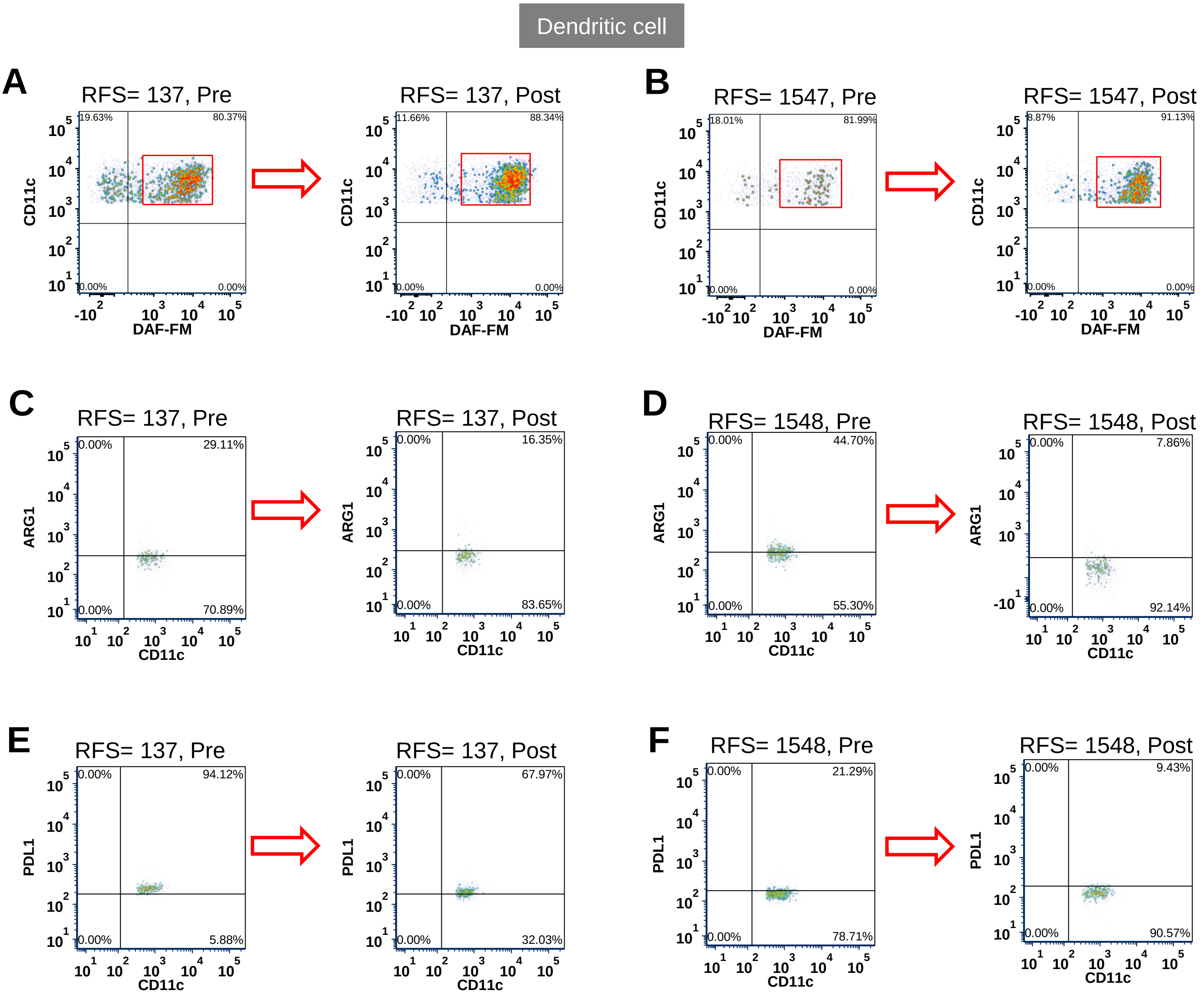

Fig. S13. PD-L1 levels are increased in dendritic cells associated with poor RFS. (A-B) Dendritic cells (node 108 = HLA-DR$^{+/lo}$CD33$^{-/lo\ or\ +/lo}$CD11b^{+}CD11c^{+}DAF-FM^{+}) with intermediate levels of NO increased overall and were not associated with RFS after treatment. There were patient subsets that did demonstrate increase RFS with a more pronounced NO increase (C-D) Arginase 1 levels minimally decrease after treatment without association with RFS. (E-F) PD-L1 has increased expression on dendritic cells prior to therapy in patients with poor RFS.

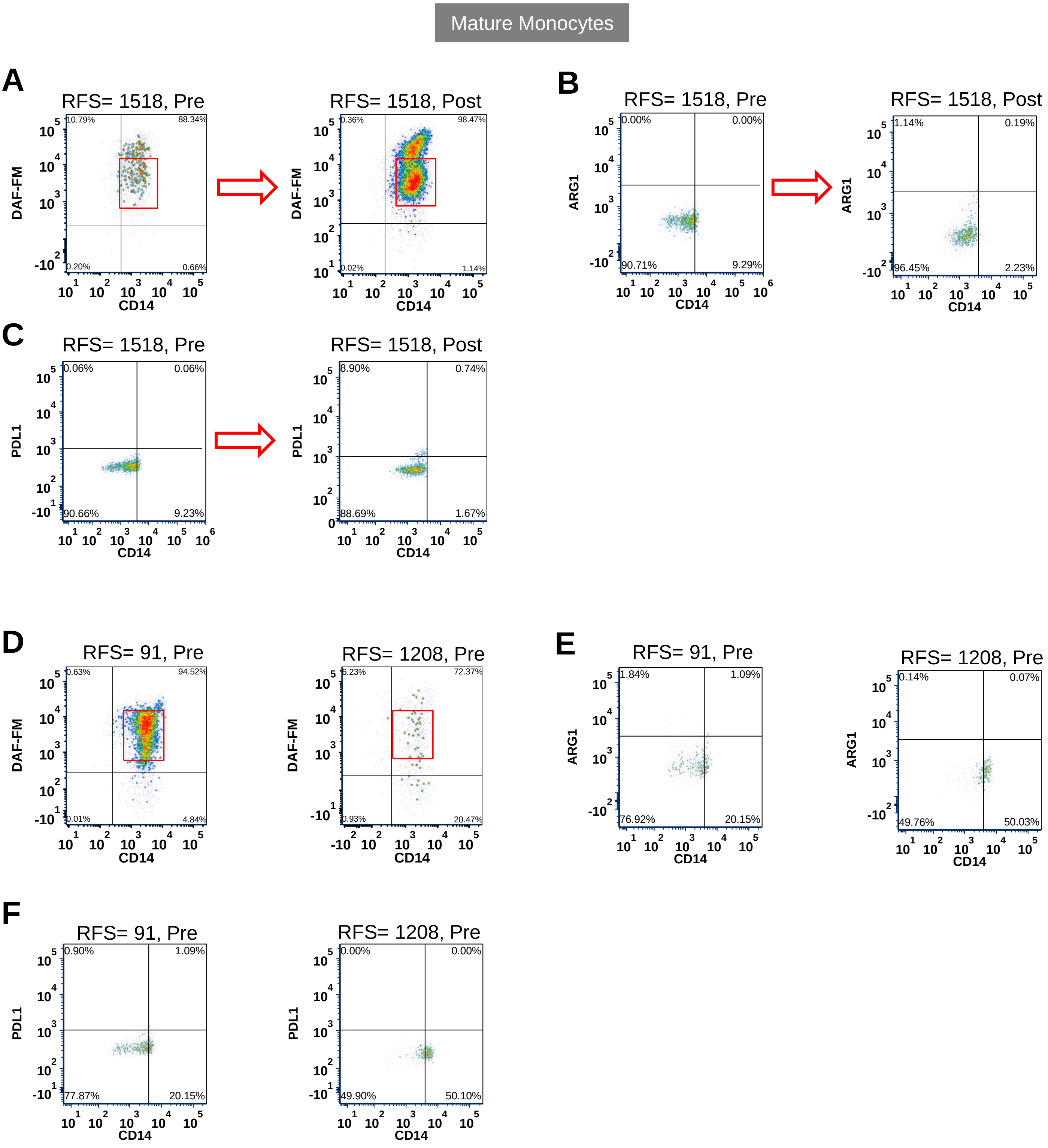

Fig. S14. Mature monocytes display high NO levels pre-treatment in PBMCs collected from patients with short RFS, but demonstrate increased expression of NO after treatment in long RFS. (A-C) Mature monocytes (node 167 = HLA-DR$^+$CD33$^+$CD11b$^+$CD11c$^+$CD14$^+$DAF-FM$^+$) with an intermediate level of NO, had an overall increase in the long-term RFS patients after treatment. Arginase 1 and PD-L1 did not show this robust trend (B-C). (D-F) Mature monocytes exhibited increased NO expression pre-treatment among patients with RFS ≤ 1 year. Again, Arginase 1 and PD-L1 did not show this trend (E-F).

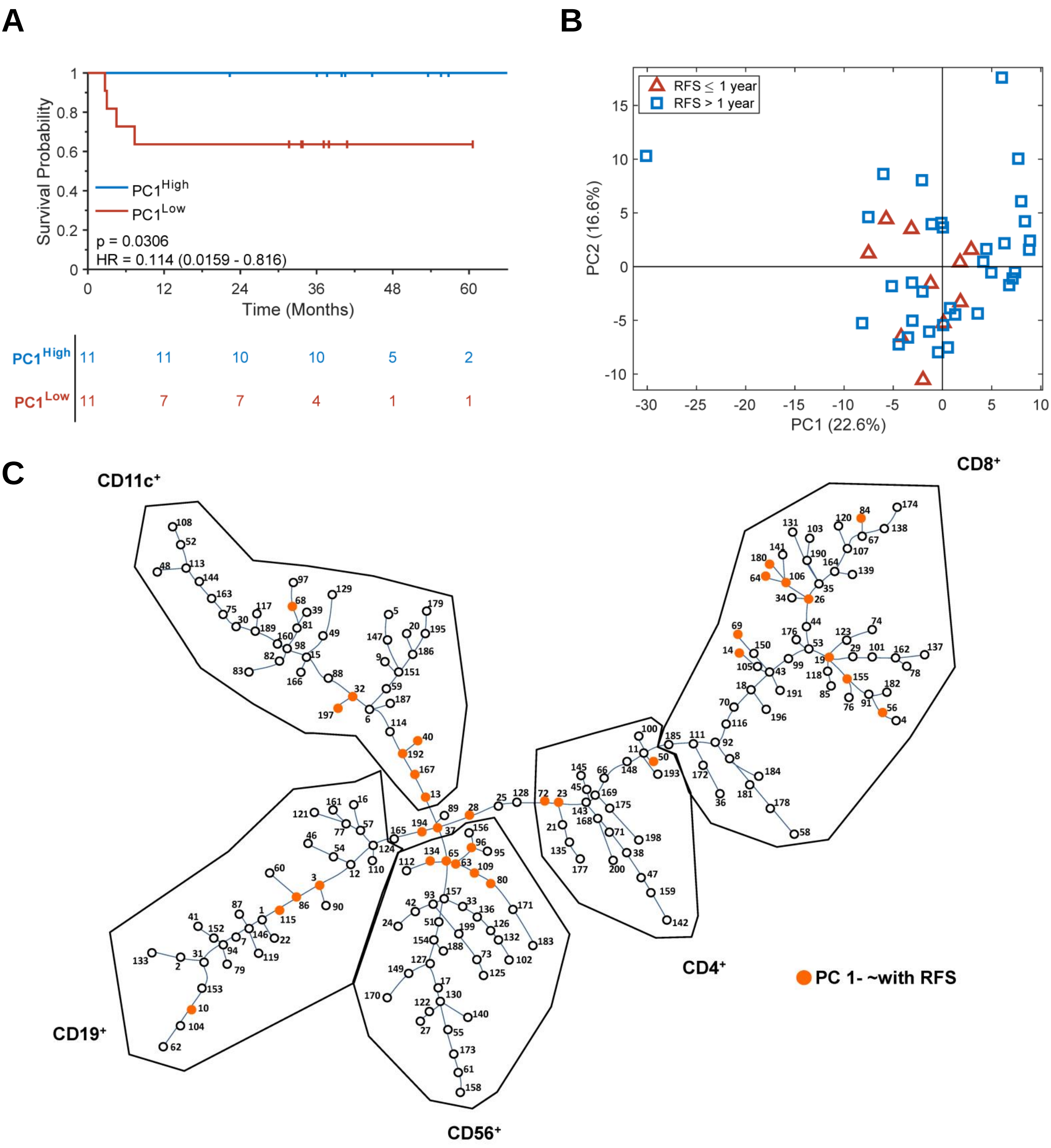

Fig. S15. Principal component analysis of clustering tree of lymphocytes isolated prior to therapy. (A) Kaplan-Meier plot demonstrates no relapses in the top quartile of principal component 1 (PC1). (B) A Biplot illustrating the distribution of contribution of PC1 and PC2 of clinical samples based on one year relapse free survival. (C) Clustering tree highlighting the nodes contributing greater than 1% towards PC1 (solid orange). And associated with RFS. We found that numbers of cells in lymphoid nodes comprising PC1 in general are associated with decreased RFS prior to therapy. These nodes that have an association with increased RFS are all T cells with increased nitric oxide levels, whereas immature B cells with NO are associated with worse outcomes. Groups of T cells associated with low NO expression are also associated with worse outcomes. The individual nodes are listed in **Table S7a**.

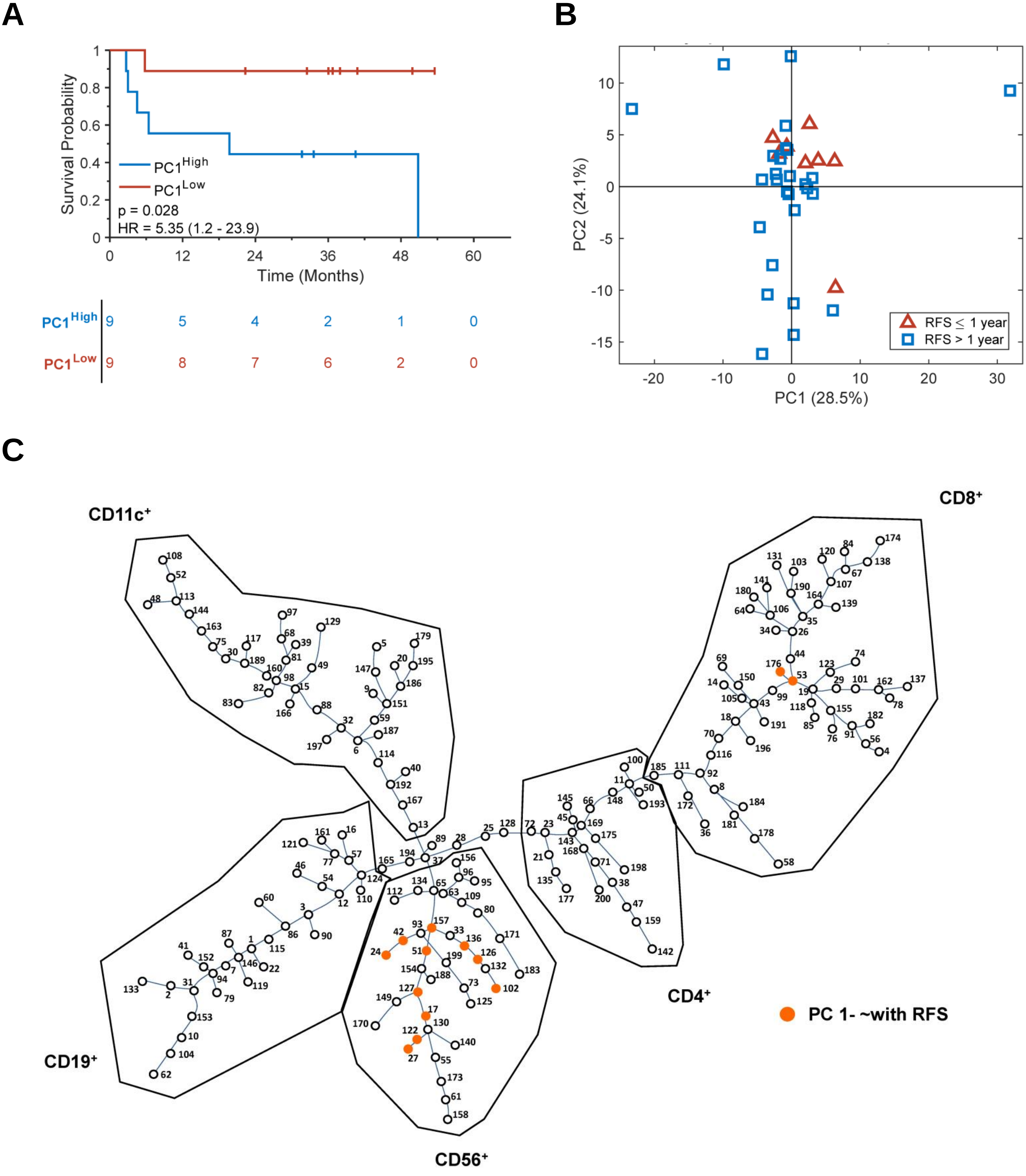

Fig. S16. Principal component analysis and clustering tree of lymphocytes (post – pre). (A) Kaplan-Meier plot illustrating the contribution of the principle component 1 on RFS (B) A Biplot illustrating the distribution of the contribution for PC1 and PC2 of clinical samples based on one year relapse free survival. (C) Clustering tree highlighting the nodes contributing to PC1 and demonstrates that the CD56 and CD8 lineage diffusely positive for NO. The Individual nodes are listed in **Table S7b**.

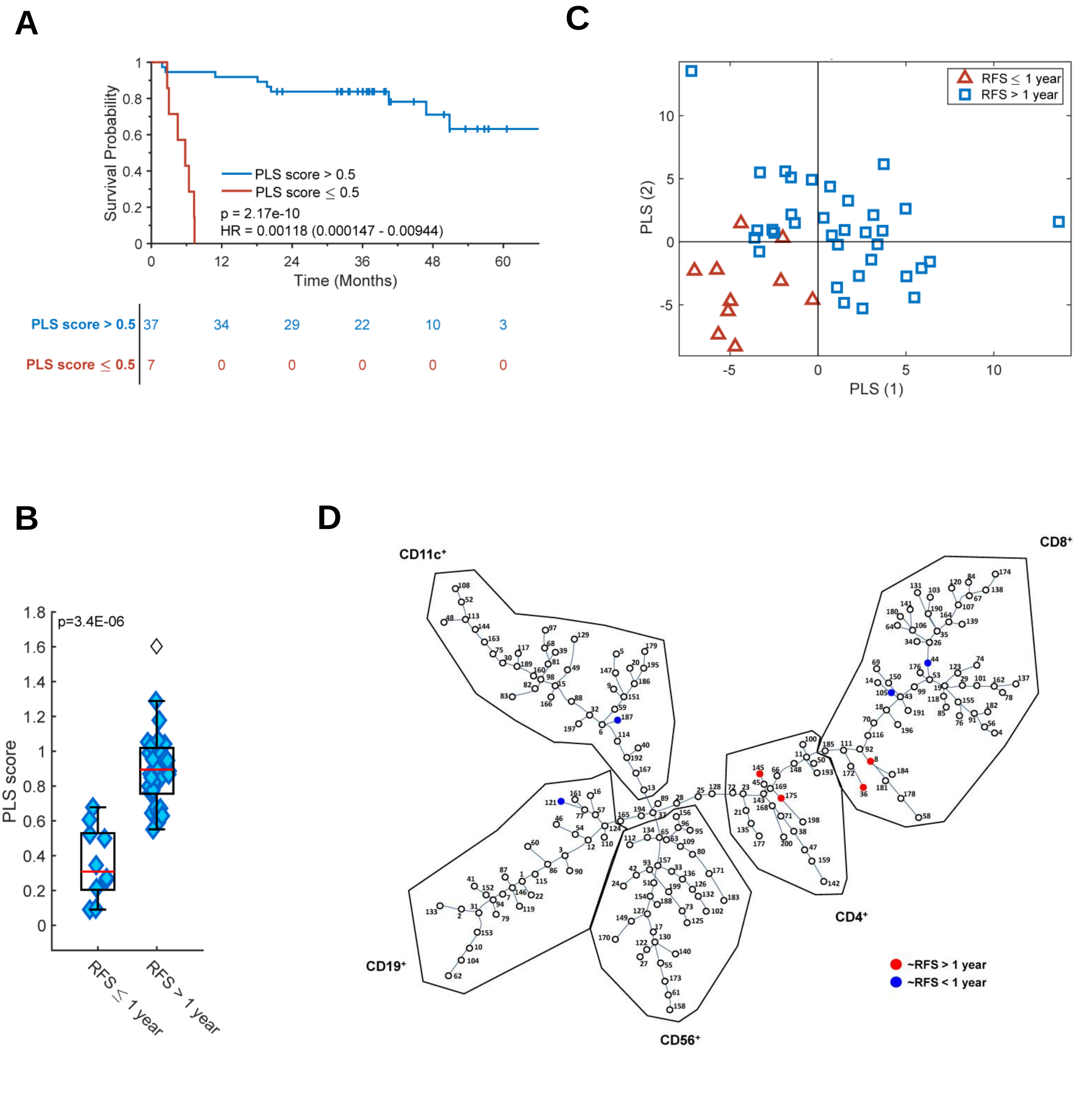

Fig. S17. PLS analysis and clustering-tree generated of lymphocytes prior to therapy. (A) Kaplan-Meier plot based on PLS score of two component analysis. (B) Histogram showing PLS scores of samples belonging to ≤ 1 year RFS and > 1 year RFS. (C) A Biplot illustrating the distribution of values for PLS1 and PLS2 of clinical samples based on one year relapse free survival. (D) Clustering tree highlighting the major nodes that were contributing towards PLS differences (solid nodes). Red nodes, denotes a positive correlation with RFS whereas, blue nodes denotes a negative correlation with RFS. The individual nodes are listed in **Table S8a**.

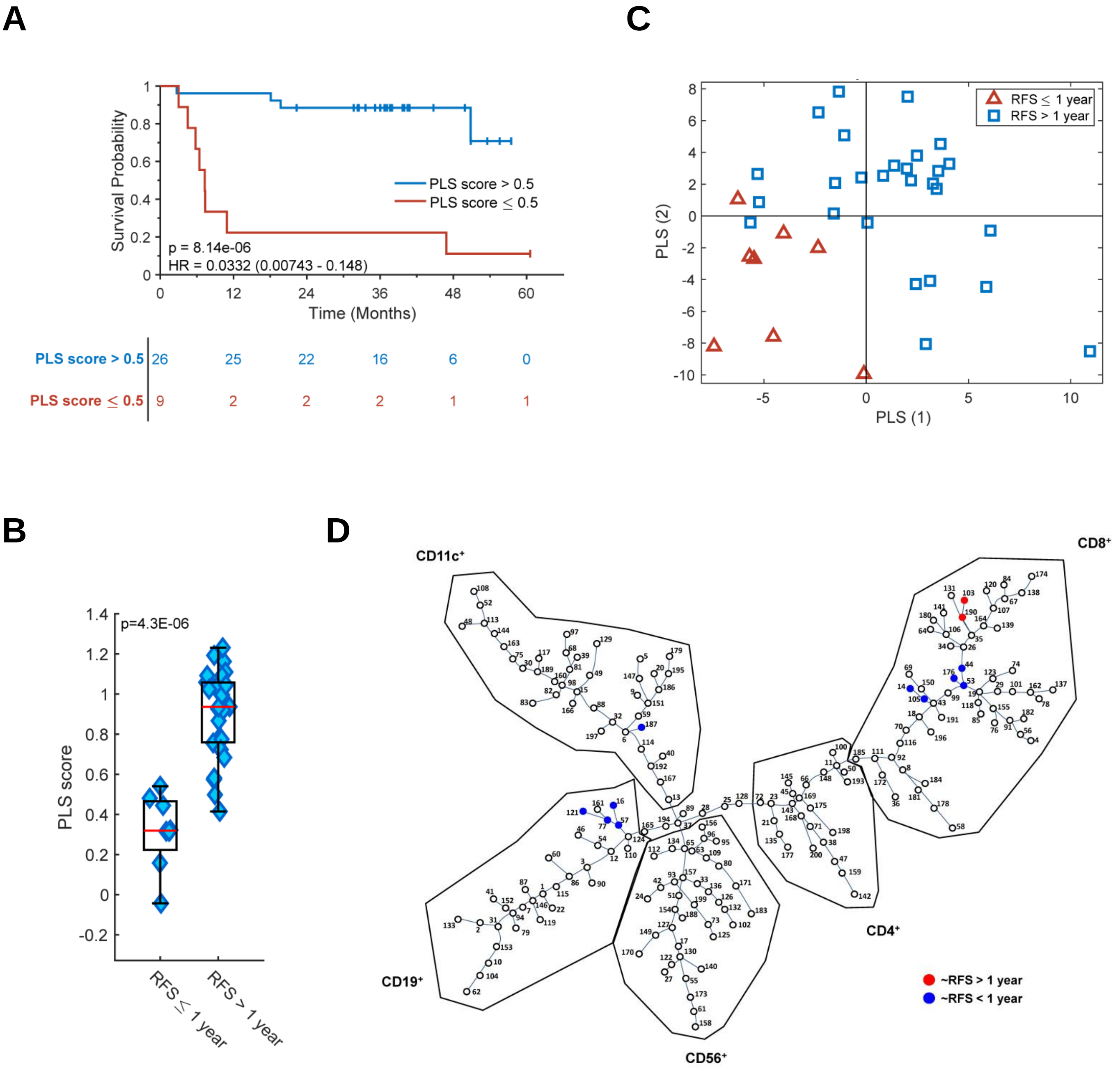

Fig. S18. PLS analysis and clustering tree generated of lymphocytes after therapy. (A) Kaplan-Meier plot based on PLS score of two component analysis. (B) Histogram showing PLS scores of samples belonging to ≤ 1 year RFS and > 1 year RFS. (C) A Biplot illustrating the distribution of values for PLS1 and PLS2 of clinical samples based on one year relapse free survival. (D) SPADE tree highlighting the major nodes that were contributing towards PLS differences (solid nodes). Red nodes, denotes a positive correlation with RFS whereas, blue nodes denotes a negative correlation with RFS. The individual nodes are listed in **Table S8b**.

Table S1a. Antibodies associated with their respective fluorochrome used to delineate lymphoid cell subsets with DAF-FM.

Specificity	Clone	Fluorochrome	Vendor
NO	n/a	DAF-FM	Invitrogen
Dead Cells	n/a	Zombie-NIR	Biolegend
CD3	SK7	BUV395	BD Biosciences
CD8	SK1	BV510	BD Biosciences
CD11c	B-ly6	PE	BD Biosciences
CD56	NCAM16.2	BV421	BD Biosciences
CD4	SK3	AF700	Biolegend
CD19	HIB19	PE-Dazzle	Biolegend
CD25	BC96	PE-Cy7	Biolegend
CD127	A019D5	APC	Biolegend

The associated isotypes for each antibody was purchased from the vendor.

Table S1b. Antibodies associated with their respective fluorochrome used to delineate myeloid cell subsets with DAF-FM.

Specificity	Clone	Fluorochrome	Vendor
NO	n/a	DAF-FM	Invitrogen
Dead Cells	n/a	Zombie-NIR	Biolegend
CD33	WM53	APC	BD Biosciences
HLADR	G46-6	PE-Cy7	BD Biosciences
CD11b	ICRF44	BV421	BD Biosciences
CD11c	B-ly6	PE	BD Biosciences
CD14	MΦP9	BUV395	BD Biosciences
CD15	W6D3	BV510	BD Biosciences

The associated isotypes for each antibody was purchased from the vendor.

Table S1c. Antibodies associated with their respective fluorochrome used to delineate lymphoid cell subsets without DAF-FM.

Specificity	Clone	Fluorochrome	Vendor
Dead Cells	n/a	Zombie-NIR	Biolegend
CD3	SK7	BUV395	BD Biosciences
CD8	SK1	BUV805	BD Biosciences
CD11c	B-ly6	BV650	BD Biosciences
CD56	NCAM16.2	BV480	BD Biosciences
CD4	SK3	BUV563	BD Biosciences
CD19	HIB19	PerCP/Cy5.5	BD Biosciences
CD25	BC96	PE-Cy7	Biolegend
CD127	A019D5	BV711	Biolegend
CD69	FN50	APC-R700	BD Biosciences
FoxP3	259D/C7	PE-CF594	BD Biosciences
IFN-γ	4S.B3	BV786	BD Biosciences
CD3z (TCR-ζ)	K25-407.69	AF647	BD Biosciences
Arginase 1	14D2C43	PE	Biolegend
CTLA4	BNI3	BV421	BD Biosciences
PD-L1	MIH1	BB515	BD Bioscience

The associated isotypes for each antibody was purchased from the vendor.

Table S1d. Antibodies associated with their respective fluorochrome used to delineate myeloid cell subsets without DAF-FM.

Specificity	Clone	Fluorochrome	Vendor
Dead Cells	n/a	Zombie-NIR	Biolegend
CD33	WM53	APC	BD Biosciences
HLA-DR	G46-6	PE-Cy7	BD Biosciences
CD11b	ICRF44	PerCP/Cy5.5	Biolegend
CD11c	B-ly6	BV650	BD Biosciences
CD14	MΦP9	BUV395	BD Biosciences
CD15	W6D3	BV510	BD Biosciences
CTLA4	BNI3	BV421	BD Biosciences
PD-L1	MIH1	BB515	BD Bioscience
Arginase 1	14D2C43	PE	Biolegend
FoxP3	259D/C7	PE-CF594	BD Biosciences
IFN-γ	4S.B3	BV786	BD Biosciences

The associated isotypes for each antibody was purchased from the vendor.

Table S2. Clinical characteristics of metastatic melanoma patients who received adjuvant ipilimumab and peptide vaccine treatment. Many patients did not progress at the end of the study and have an RFS censor value of 0.

Patient	ID pre	ID Post	dose	Gender	Stage	Study Age	RFS Censor	RFS (days)
1	10836	10897	3	F	III	32	1	195
2	10851	10888	3	F	IV	55	1	551
3	10877	10912	3	F	III	65	0	1216
4	10885	10918	3	M	III	78	0	1362
5	10889	10917	3	F	III	36	0	1692
6	10909	10935	3	F	IV	58	0	680
7	10944	10966	10	M	IV	61	0	1749
8	10945	10967	10	M	III	36	0	1233
9	10947	10971	10	M	III	59	1	600
10	10948	10965	10	F	IV	43	0	1208
11	10949	10970	10	M	III	58	0	1548
12	10959	10977	10	M	IV	43	0	1630
13	10960	10975	10	M	III	64	0	984
14	10984	11006	10	F	IV	61	0	1071
15	10985	10999	10	M	IV	21	1	91
16	10988	11000	10	F	IV	28	1	82
17	10989	11013	10	M	III	62	1	224
18	10992	11021	10	M	III	46	1	1547
19	10993	11022	10	M	IV	63	1	332
20	10997	11023	10	F	IV	53	1	222
21	10998	11024	10	F	IV	51	0	1242
22	11001	11030	10	M	IV	40	0	1844
23	11002	11031	10	M	III	50	0	1130
24	11004	11034	10	M	IV	65	0	1146
25	11005	11035	10	M	III	47	1	137
26	11007	11044	10	F	IV	51	0	1155
27	11012	11042	10	M	III	70	1	1426
28	11014	11047	10	F	III	78	0	1097
29	11016	11046	10	F	III	29	0	989
30	11017	11052	10	M	III	61	1	176
31	11019	11050	10	M	IV	64	0	1118
32	11029	11056	10	M	IV	65	0	1023
33	11036	11058	10	M	IV	39	0	964
34	11038	11067	10	M	IV	63	0	964
35	11048	11064	10	M	IV	63	0	1518
36	10844		3	F	III	41	0	2273
37	10880		3	F	IV	63	0	1728
38	10893		3	M	III	70	0	2156
39	10946		10	M	IV	42	1	1232
40	10996		10	M	III	44	1	56
41	11010		10	M	IV	70	1	621
42	11020		10	M	IV	44	1	74
43	11025		10	F	III	56	0	652
44	11026		10	M	III	66	0	1029

Table S3. Phenotypic characteristics of lymphoid immune cells (nodes) associated with treatment effects from the MPATR output.

**Table S3. Lymphoid cell phenotypes (nodes) associated with treatment effects from the MPATR analysis**				
**Node**	**Phenotype**	**Cell type**	**Cell Range**	**Statistics**
3	CD19^{+}CD25$^{-/lo}$DAF-FM$^{-/lo}$	B Cell	41 to 3817	***Cox.1,*** exp(coef) = 1.64, p = 0.049<br>***Cox.4,*** exp(coef) = 0.666, p = 0.046
60	CD19^{+}CD11c$^{-/lo}$CD25$^{-/lo\ or\ +/lo}$DAF-FM$^{-/lo}$	B Cell	3 to 869	***Cox.1,*** exp(coef) = 2.130, p = 0.011<br>***Cox.2,*** exp(coef) = 0.357, p = 0.024<br>***Cox.4,*** exp(coef) = 0.515, p = 0.004<br>***Wilc.4,*** RFS(<1yr, median = -1.024),<br>RFS(>1yr, median = 0.421), p = 0.027
86	CD19^{+}CD25$^{-/lo}$DAF-FM$^{-/lo\ or\ +/lo}$	B Cell	16 to 4809	***Cox.1,*** exp(coef) = 1.777, p = 0.034<br>***Cox.4,*** exp(coef) = 0.668, p = 0.030
90	CD19^{+}CD25$^{-/lo}$DAF-FM$^{-/lo}$	B Cell	5 to 1214	***Cox.1,*** exp(coef) = 1.983, p = 0.027
115	CD19^{+}CD11c$^{-/lo}$CD25$^{-/lo}$DAF-FM$^{-/lo}$	B Cell	9 to 1899	***Cox.4,*** exp(coef) = 0.677, p = 0.047
143	CD3^{+}CD4^{+}CD127^{+}CD25$^{-/lo}$DAF-FM^{+}	CD4 Naive or Memory T Cell	486 to 11473	***Wilc.3,*** Mean = -0.342, p = 0.002
38	CD3^{+}CD4^{+}CD127$^{+/lo}$CD25$^{-/lo}$DAF-FM^{+}	CD4 Naive or Memory T Cell	149 to 4191	***Wilc.3,*** Mean = -0.363, p = 0.022
45	CD3^{+}CD4^{+}CD127^{+}CD25$^{-/lo}$DAF-FM^{+}	CD4 Naive or Memory T Cell	358 to 20858	***Wilc.3,*** Mean = -0.359, p = 0.010
175	CD3^{+}CD4^{+}CD127^{+}CD25$^{-/lo\ or\ +/lo}$DAF-FM^{+}	CD4 Naive or Memory T Cell	3 to 6713	***Wilc.1,*** RFS(<1yr, mean = -11.003, SD =0.855),<br>RFS(>1yr, mean = -10.123, SD = 0.832), p = 0.007
25	CD3^{+}CD4$^{+/lo}$CD127$^{-/lo}$CD25$^{-/lo}$	CD4 T Cell	98 to 5518	***Wilc.3,*** Mean = -0.615, p = 0.002
66	CD3^{+}CD4^{+}CD127$^{-/lo}$CD25$^{-/lo}$DAF-FM^{+}	CD4 T Cell	95 to 2686	***Wilc.3,*** Mean = -0.652, p = 0.001
128	CD3^{+}CD4$^{-\ or\ +/lo}$CD127$^{-/lo}$CD25$^{-/lo}$DAF-FM$^{-/lo}$	CD4 T Cell	72 to 3796	***Wilc.3,*** Mean = -0.519, p = 0.006
177	CD3^{+}CD4^{+}CD127^{+}CD25^{+}CD11c$^{-/lo}$DAF-FM$^{-/lo}$	CD4 T Cell	0 to 527	***Cox.4,*** exp(coef) = 0.512, p = 0.016
36	CD3^{+}CD127$^{+/lo}$DAF-FM^{+}	CD4^{-}CD8^{-}αβT Cell	14 to 7534	***Cox.4*** , exp(coef) = 1.759, p =0.026<br>***Wilc.4*** , RFS(<1yr, median = 0.642),<br>RFS(>1yr, median = -0.053), p = 0.007
180	CD3^{+}CD127$^{+/lo}$DAF-FM$^{+/lo}$	CD4^{-}CD8^{-}αβT Cell	9 to 693	***Wilc.2,*** RFS(<1yr, mean = -10.618, SD = 0.387),<br>RFS(>1yr, mean = -11.174, SD = 0.734), p = 0.010
185	CD3^{+}CD127$^{+\ or\ +/lo}$CD25$^{-/lo}$DAF-FM^{+}	CD4^{-}CD8^{-}αβT Cell	15 to 1335	***Wilc.4,*** RFS(<1yr, median = 0.510),<br>RFS(>1yr, median = -0.084), p = 0.041
26	CD3^{+}CD8^{+}CD127^{+}DAF-FM$^{-/lo}$	CD8 Naive or Memory T Cell	34 to 1630	***Cox.1,*** exp(coef) = 3.635, p = 0.031<br>***Wilc.1,*** RFS(<1yr, mean = -7.843, SD =0.402),<br>RFS(>1yr, mean = -8.227, SD = 0.506), p = 0.038<br>***Wilc.2*** , RFS(<1yr, mean = -7.750, SD =0.611),<br>RFS(>1yr, mean = -8.216, SD = 0.549), p = 0.022
84	CD3^{+}CD8^{+}CD127^{+}DAF-FM$^{-/lo}$	CD8 Naive or Memory T Cell	40 to 2548	***Wilc.1*** , RFS(<1yr, mean = -8.208, SD =0.227),<br>RFS(>1yr, mean = -8.537, SD = 0.486), p = 0.038<br>***Wilc.2,*** RFS(<1yr, mean = -8.023, SD = 0.544),<br>RFS(>1yr, mean = -8.414, SD = 0.528), p = 0.037
150	CD3^{+}CD8^{+}CD127$^{+\ or\ +/lo}$CD25$^{-/lo}$DAF-FM^{+}	CD8 Naive or Memory T Cell	85 to 2331	***Wilc.2,*** RFS(<1yr, mean = -6.979, SD = 0.583),<br>RFS(>1yr, mean = -7.421, SD = 0.577), p = 0.037
44	CD3^{+}CD8^{+}CD127$^{+/lo}$DAF-FM^{+}	CD8 Naive or Memory T Cell	77 to 2542	***Wilc.1*** , RFS(<1yr, mean = -6.874, SD =0.464),<br>RFS(>1yr, mean = -7.274, SD = 0.533), p = 0.024

Table S3. Lymphoid cell phenotypes (nodes) associated with treatment effects from the MPATR analysis				
**Node**	**Phenotype**	**Cell type**	**Cell Range**	**Statistics**
53	CD3^{+}CD8^{+}CD127$^{+/lo}$DAF-FM^{+}	CD8 Naive or Memory T Cell	91 to 3527	***Cox.2,*** exp(coef) = 2.787, p = 0.047<br>***Wilc.2*** , RFS(<1yr, mean = -6.628, SD =0.614),<br>RFS(>1yr, mean = -7.191, SD = 0.642), p = 0.030
139	CD3^{+}CD8^{+}CD127$^{\text{-/lo or +/lo}}$DAF-FM$^{-/lo}$	CD8 Naive or Memory T Cell	40 to 1863	***Wilc.3,*** Mean = -0.274, p = 0.024
172	CD3^{+}CD8$^{+/lo}$CD127$^{+/lo}$CD25$^{-/lo}$CD11c$^{-/lo}$DAF-FM^{+}	CD8 Naive or Memory T Cell	14 to 1076	***Wilc.4,*** RFS(<1yr, median = 0.564),<br>RFS(>1yr, median = -0.206), p = 0.030
27	CD56^{+}CD8$^{+/lo}$CD11c^{+}DAF-FM^{+}	CD8^{+} NK	11 to 1113	***Wilc.3,*** Mean = 0.319, p = 0.014
122	CD56^{+}CD8^{+}CD11c^{+}CD25$^{-/lo}$DAF-FM^{+}	CD8^{+} NK	10 to 2521	***Wilc.3,*** Mean = 0.343, p = 0.011<br>***Wilc.4,*** RFS(<1yr, median = 0.271),<br>RFS(>1yr, median = -0.575), p = 0.037
126	CD56^{+}CD8^{+}CD11c^{+}DAF-FM^{+}	CD8^{+} NK	5 to 1036	***Wilc.3,*** Mean = 0.382, p = 0.012<br>***Cox.4,*** exp(coef) = 1.484, p = 0.040
132	CD56^{+}CD8$^{+/lo}$CD11c^{+}CD25$^{-/lo}$DAF-FM^{+}	CD8$^{+/lo}$ NK	9 to 940	***Wilc.3,*** Mean = 0.347, p = 0.012
173	CD56^{+}CD8$^{+/lo}$CD11c^{+}CD127$^{+/lo}$CD25$^{-/lo}$DAF-FM^{+}	CD8$^{+/lo}$ NK	1 to 575	***Wilc.3,*** Mean = 0.352, p = 0.046<br>***Cox.4,*** exp(coef) = 1.596, p = 0.029
14	CD3^{+}CD8^{+}CD127$^{-/lo}$CD25$^{\text{- or -/lo}}$DAF-FM^{+}	Effector T Cell	58 to 4400	***Wilc.3*** , Mean = -0.300, p = 0.011
35	CD3^{+}CD8^{+}CD127$^{-/lo}$CD25$^{-/lo}$CD11c$^{-/lo}$DAF-FM$^{-/lo}$	Effector T Cell	29 to 1662	***Wilc.3,*** Mean = -0.306, p = 0.029
69	CD3^{+}CD8^{+}CD127$^{-/lo}$DAF-FM^{+}	Effector T Cell	10 to 1708	***Wilc.3,*** Mean = -0.370, p = 0.012
107	CD3^{+}CD8^{+}CD127$^{-/lo}$DAF-FM$^{-/lo}$	Effector T Cell	30 to 2866	***Wilc.3,*** Mean = -0.281, p = 0.024
155	CD3^{+}CD8^{+}CD25$^{-/lo}$DAF-FM^{+}	Effector T Cell	4 to 5945	***Wilc.1,*** RFS(<1yr, mean = -8.666, SD =0.694),<br>RFS(>1yr, mean = -8.066, SD = 0.956), p = 0.035
164	CD3^{+}CD8^{+} CD25$^{-/lo}$CD11c$^{\text{- or -/lo}}$DAF-FM$^{\text{-/lo or +/lo}}$	Effector T Cell	34 to 2432	***Wilc.3,*** Mean = -0.233, p = 0.037
67	CD3^{+}CD8^{+}CD11c$^{\text{- or -/lo}}$DAF-FM$^{-/lo}$	Effector T Cell	31 to 2211	***Wilc.3,*** Mean = -0.395, p = 0.019
24	CD56^{+}CD11c$^{+/lo}$DAF-FM^{+}	NK Cell	1 to 1096	***Cox.4*** , exp(coef) = 1.610, p = 0.019<br>***Wilc.4*** , RFS(<1yr, median = 0.438),<br>RFS(>1yr, median = -0.506), p = 0.017
158	CD56^{+}CD11c^{+}CD127$^{+/lo}$CD25$^{-/lo}$DAF-FM^{+}	NK Cell	8 to 850	***Cox.4,*** exp(coef) = 1.700, p = 0.021
42	CD56$^{+/lo}$CD11c^{+}CD25$^{-/lo}$DAF-FM^{+}	NK Cell	9 to 720	***Cox.4*** , exp(coef) = 1.659, p =0.018<br>***Wilc.4*** , RFS(<1yr, median = 0.670),<br>RFS(>1yr, median = -0.444), p = 0.005
55	CD56^{+}CD8$^{-/lo}$CD11c^{+}CD127$^{+/lo}$CD25$^{-/lo}$DAF-FM^{+}	NK Cell	5 to 878	***Cox.2,*** exp(coef) = 2.618, p = 0.044<br>***Cox.4,*** exp(coef) = 1.898, p = 0.012
93	CD56$^{+/lo}$CD11c^{+}CD25$^{-/lo}$DAF-FM^{+}	NK Cell	4 to 645	***Cox.4,*** exp(coef) = 1.574, p = 0.046

Table S3. Lymphoid cell phenotypes (nodes) associated with treatment effects from the MPATR analysis				
**Node**	**Phenotype**	**Cell type**	**Cell Range**	**Statistics**
135	CD3^{+}CD4$^{+/lo}$CD56$^{+/lo\ or\ -/lo}$CD11c$^{-/lo}$CD25$^{-/lo}$DAF-FM^{-}	NK-like Cell	5 to 2468	***Wilc.3,*** Mean = -0.456, p = 0.044
174	CD3^{+}CD8^{+}CD56$^{-/lo\ or\ +/lo}$CD11c$^{-/lo}$CD127^{+}CD25$^{+/lo}$DAF-FM$^{-/lo}$	NK-like Cell	1 to 475	***Cox.1,*** exp(coef) = 3.482, p = 0.008<br>***Wilc.1,*** RFS(<1yr, mean = -9.840, SD =0.334),<br>RFS(>1yr, mean = -10.243, SD = 0.571), p = 0.009
159	CD3^{+}CD4^{+}CD127$^{-/lo}$CD25^{+}DAF-FM^{+}	T Regulatory Cell	38 to 2525	***Wilc.3,*** Mean = -0.325, p = 0.029
200	CD3^{+}CD4$^{+/lo}$CD11c$^{-/lo}$CD19^{+}CD127$^{+/lo}$CD25$^{+/lo}$DAF-FM^{+}	Artifact	2 to 156	***Wilc.3,*** Mean = -0.523, p = 0.021
13	CD11c^{+}CD25$^{-/lo}$DAF-FM$^{+/lo}$	-	26 to 5650	***Cox.4,*** exp(coef) = 0.510, p = 0.025
30	CD11c^{+}CD56$^{-/lo}$CD25$^{+/lo}$DAF-FM^{+}	-	3 to 1911	***Wilc.3,*** Mean = 0.283, p = 0.049
39	CD11c^{+}CD25$^{+/lo}$DAF-FM^{+}	-	34 to 2699	***Wilc.3,*** Mean = 0.353, p = 0.039
83	CD11c^{+}CD56$^{-/lo}$CD25$^{+/lo}$DAF-FM$^{+/lo}$	-	31 to 2390	***Wilc.3,*** Mean = 0.289, p = 0.024
98	CD11c^{+}CD4$^{-/lo}$CD56$^{-\ or\ -/lo}$CD25$^{+/lo}$DAF-FM^{+}	-	32 to 11637	***Wilc.3,*** Mean = 0.375, p = 0.021
124	CD11c$^{-/lo\ or\ +/lo}$CD25$^{-/lo\ or\ +/lo}$DAF-FM$^{-/lo}$	-	1 to 855	**Wilc.3**, Mean = 0.696, p = 0.036<br>**Wilc.4,** RFS(<1yr, median = 0.218),<br>RFS(>1yr, median = -1.041), p = 0.046
151	CD11c^{+}CD25$^{-/lo}$DAF-FM^{+}	-	22 to 3483	***Cox.4,*** exp(coef) = 0.445, p = 0.049<br>***Wilc.4,*** RFS(<1yr, median = -1.072),<br>RFS(>1yr, median = -0.042), p = 0.027
160	CD11c^{+}CD25^{+}DAF-FM^{+hi}	-	6 to 4209	***Wilc.3,*** Mean = 0.409, p = 0.015
161	CD11c$^{+/lo}$CD25$^{-/lo}$DAF-FM$^{+/lo}$	-	2 to 785	***Wilc.2,*** RFS(<1yr, mean = -9.266 SD = 0.706),<br>RFS(>1yr, mean = -9.883, SD = 0.790), p = 0.041
166	CD11c^{+}CD56$^{-/lo}$CD25$^{+/lo}$DAF-FM^{+}	-	20 to 5854	***Wilc.3,*** Mean = 0.350, p = 0.027
167	CD11c^{+}CD25$^{-/lo\ or\ +/lo}$DAF-FM$^{+/lo}$	-	37 to 3753	***Cox.4,*** exp(coef) = 0.397, p = 0.040
183	CD11c^{+}CD56$^{-/lo}$CD25^{+}CD3$^{-/lo}$CD4$^{-\ or\ -/lo}$DAF-FM$^{-\ or\ -/lo}$	-	0 to 633	***Wilc.3,*** Mean = 0.374, p = 0.037,<br>***Cox.4,*** exp(coef) = 0.388, p = 0.005
189	CD11c^{+}CD25$^{+/lo}$CD56$^{-/lo}$DAF-FM^{+}	-	0 to 3822	***Wilc.3,*** Mean = 0.373, p = 0.023
197	CD11c^{+}CD56$^{-/lo}$CD4$^{-/lo}$CD3$^{-/lo}$CD25$^{+/lo}$DAF-FM$^{+/lo}$	-	0 to 690	***Wilc.3,*** Mean = 0.357, p = 0.012<br>***Cox.4,*** exp(coef) = 0.368, p = 0.018
199	CD11c$^{-/lo\ or\ +/lo}$CD4$^{-/lo\ or\ +/lo}$CD25$^{-/lo}$DAF-FM^{+}	-	1 to 256	***Wilc.3,*** Mean = 0.281, p = 0.042

Table S4. Phenotypic characteristics lymphoid immune cells (nodes) with the addition of scatter properties FSC and SSC associated with treatment effects from the MPATR output.

Table S4. Lymphoid FSC/SSC cell phenotypes (nodes) associated with treatment effects from the MPATR analysis				
**Node**	**Phenotype**	**Cell type**	**Cell Range**	**Statistics**
20	$CD19^{+}CD11c^{-/lo}CD25^{-/lo \text{ or } +/lo}DAF\text{-}FM^{-/lo \text{ or } +/lo}$	B Cell	5 to 1256	***Cox.1,*** exp(coef) = 2.265, p = 0.018<br>***Cox.4,*** exp(coef) = 0.497, p = 0.036
29	$CD19^{+}CD11c^{-/lo}CD25^{-/lo \text{ or } +/lo}DAF\text{-}FM^{-/lo \text{ or } +/lo}$	B Cell	23 to 3592	***Cox.1,*** exp(coef) = 1.851, p = 0.025<br>***Cox.4,*** exp(coef) = 0.640, p = 0.023
132	$CD19^{+}CD11c^{+/lo}CD25^{+/lo}CD56^{- \text{ or } -/lo}DAF\text{-}FM^{+}$	B Cell	0 to 722	***Cox.1,*** exp(coef) = 1.977, p = 0.040
197	$CD19^{+}CD11c^{+}CD25^{+/lo}DAF\text{-}FM^{+/lo}$	B Cell	5 to 344	***Cox.1,*** exp(coef) = 2.195, p = 0.034
56	$CD19^{+}CD11c^{- \text{ or } -/lo}CD25^{-/lo \text{ or } +/lo}DAF\text{-}FM^{-/lo \text{ or } +/lo}$	B Cell	26 to 1495	***Cox.4,*** exp(coef) = 0.638, p = 0.048
153	$CD19^{+}CD11c^{+}CD25^{+/lo}DAF\text{-}FM^{+/lo}$	B Cell	3 to 318	***Cox.1,*** exp(coef) = 2.013, p = 0.045<br>***Cox.4,*** exp(coef) = 0.527, p = 0.017
138	$CD19^{+}CD25^{-/lo}DAF\text{-}FM^{-/lo}$	B Cell	13 to 3392	***Cox.1,*** exp(coef) = 1.677, p = 0.046
91	$CD3^{+}CD4^{+}CD127^{+}CD25^{- \text{ or } -/lo}DAF\text{-}FM^{+}$	CD4 Naive or Memory T Cell	118 to 10702	***Wilc.3,*** Mean = -0.285, p = 0.025
61	$CD3^{+}CD4^{+}CD127^{+ \text{ or } +/lo}CD25^{-/lo \text{ or } +/lo}DAF\text{-}FM^{+}$	CD4 Naive or Memory T Cell	10 to 6148	***Wilc.1,*** RFS(<1yr, mean = -9.901, SD = 0.439),<br>RFS(>1yr, mean = -9.364, SD = 0.694), p = 0.0096<br>***Wilc.3,*** Mean = -0.686, p = 0.0001
65	$CD3^{+}CD4^{+}CD127^{+/lo}CD25^{-\text{or } -/lo}DAF\text{-}FM^{+}$	CD4 Naive or Memory T Cell	263 to 6638	***Wilc.3,*** Mean = -0.298, p = 0.007
159	$CD3^{+}CD4^{+}CD127^{+}CD25^{- \text{ or } -/lo}DAF\text{-}FM^{+}$	CD4 Naive or Memory T Cell	7 to 8257	***Wilc.1,*** RFS(<1yr, mean = -9.387, SD =0.776),<br>RFS(>1yr, mean = -8.501, SD = 0.860), p = 0.011<br>***Cox.4,*** exp(coef) = 2.383, p = 0.006<br>***Wilc.4,*** RFS(<1yr, median = 1.201),<br>RFS(>1yr, median = 0.025), p = 0.024
73	$CD3^{+}CD4^{+}CD127^{+}CD25^{- \text{ or } -/lo}DAF\text{-}FM^{+}$	CD4 Naive or MemoryT Cell	88 to 13986	***Wilc.3,*** Mean = -0.393, p = 0.020
144	$CD3^{+}CD4^{+/lo}CD127^{- \text{ or } -/lo}CD25^{-/lo \text{ or } +/lo}DAF\text{-}FM^{-/lo}$	CD4 T Cell	49 to 2224	***Wilc.3,*** Mean = -0.531, p = 0.003
150	$CD3^{+}CD4^{+}CD127^{-/lo \text{ or } +/lo}CD25^{+/lo}DAF\text{-}FM^{+}$	CD4 T Cell	112 to 2198	***Wilc.3,*** Mean = -0.311, p = 0.024
154	$CD3^{+}CD4^{+}CD127^{- \text{ or } -/lo}CD25^{- \text{ or } -/lo}DAF\text{-}FM^{+}$	CD4 T Cell	73 to 3426	***Wilc.3,*** Mean = -0.671, p = 0.001
181	$CD3^{+}CD4^{+}CD127^{-/lo}CD25^{-/lo}DAF\text{-}FM^{-/lo}$	CD4 T Cell	128 to 4674	***Wilc.3,*** Mean = -0.612, p = 0.001
54	$CD3^{+}CD8^{-/lo}CD127^{+}CD25^{- \text{ or } -/lo}DAF\text{-}FM^{+}$	$CD4^{-}CD8^{-}$αβT Cell	16 to 961	***Wilc.4,*** RFS(<1yr, median = 0.518),<br>RFS(>1yr, median = -0.153), p = 0.030
166	$CD3^{+}CD127^{+}DAF\text{-}FM^{+}$	$CD4^{-}CD8^{-}$αβT Cell	28 to 4434	***Wilc.4,*** RFS(<1yr, median = 0.454),<br>RFS(>1yr, median = -0.213), p = 0.046
80	$CD3^{+}CD8^{+}CD127^{+}DAF\text{-}FM^{-/lo}$	CD8 Naive or Memory T Cell	70 to 4194	***Cox.1,*** exp(coef) = 3.022, p = 0.032<br>***Wilc.1,*** RFS(<1yr, mean = -7.334, SD = 0.404),<br>RFS(>1yr, mean = -7.732, SD = 0.541), p = 0.031<br>***Wilc.2,*** RFS(<1yr, mean = -7.210, SD = 0.595),<br>RFS(>1yr, mean = -7.632, SD = 0.577), p = 0.034
176	$CD3^{+}CD8^{+}CD127^{+}CD11c^{- \text{ or } -/lo}DAF\text{-}FM^{+/lo}$	CD8 Naive or Memory T Cell	30 to 1656	***Wilc.3,*** Mean = -0.133, p = 0.036

Table S4. Lymphoid FSC/SSC cell phenotypes (nodes) associated with treatment effects from the MPATR analysis				
**Node**	**Phenotype**	**Cell type**	**Cell Range**	**Statistics**
49	CD3^{+}CD8^{+}CD56$^{-/lo}$CD127^{+}CD25$^{-\text{ or }-/lo}$DAF-FM^{+}	NK-like Cell	125 to 3817	***Wilc.2,*** RFS(<1yr, mean = -6.239, SD = 0.585),<br>RFS(>1yr, mean = -6.735, SD = 0.553), p = 0.046
74	CD3^{+}CD8^{+}CD127^{+}CD25$^{-\text{ or }-/lo}$DAF-FM$^{-/lo}$	CD8 Naive or Memory T Cell	22 to 1067	***Cox.1,*** exp(coef) = 4.137, p = 0.026<br>***Wilc.1,*** RFS(<1yr, mean = -8.151, SD = 0.308),<br>RFS(>1yr, mean = -8.529, SD = 0.472), p = 0.019<br>***Wilc.2,*** RFS(<1yr, mean = -8.051, SD = 0.613),<br>RFS(>1yr, mean = -8.492, SD = 0.555), p = 0.030
121	CD3^{+}CD8^{+}CD127^{+}CD25$^{-\text{ or }-/lo}$DAF-FM^{+}	CD8 Naive or Memory T Cell	38 to 2066	***Wilc.2*** **,** RFS(<1yr, mean = -6.965, SD = 0.528),<br>RFS(>1yr, mean = -7.591, SD = 0.680), p = 0.022<br>***Cox.4,*** exp(coef) = 2.205, p = 0.041
41	CD3^{+}CD8^{+}CD11c$^{-/lo\text{ or }+/lo}$CD127^{+}CD25$^{-/lo}$DAF-FM^{+}	CD8 Naive or Memory T Cell	107 to 3631	***Cox.2,*** exp(coef) = 3.715, p = 0.046<br>***Wilc.2,*** RFS(<1yr, mean = -6.135, SD = 0.554),<br>RFS(>1yr, mean = -6.606, SD = 0.482), p = 0.022
196	CD3^{+}CD8^{+}CD127$^{-/lo\text{ or }+/lo}$CD25$^{-/lo\text{ or }+/lo}$DAF-FM^{+}	CD8 T Cell	8 to 914	***Wilc.3,*** Mean = -0.592, p = 0.0002
42	CD56^{+}CD8^{+}CD11c^{+}DAF-FM$^{-/lo}$	CD8^{+} NK	4 to 3670	***Wilc.3,*** Mean = 0.404, p = 0.0130
111	CD56^{+}CD8^{+}CD11c^{+}CD127$^{+/lo}$CD25$^{-\text{ or }-/lo}$DAF-FM^{+}	CD8^{+} NK	0 to 284	***Wilc.4,*** RFS(<1yr, median = 1.151),<br>RFS(>1yr, median = -0.460), p = 0.041
117	CD56^{+}CD8^{+}CD11c^{+}DAF-FM^{+}	CD8^{+} NK	0 to 1029	***Cox.1,*** exp(coef) = 0.600, p = 0.042<br>***Wilc.3,*** Mean = 0.403, p = 0.022<br>***Cox.4,*** exp(coef) = 1.568, p = 0.011<br>***Wilc.4,*** RFS(<1yr, median = 0.581),<br>RFS(>1yr, median = -0.572), p = 0.012
122	CD56^{+}CD8^{+}CD11c$^{-/lo}$CD25$^{-\text{ or }-/lo}$DAF-FM^{+}	CD8^{+} NK	19 to 2622	***Cox.4,*** exp(coef) = 1.543, p = 0.031<br>***Wilc.4,*** RFS(<1yr, median = 0.553),<br>RFS(>1yr, median = -0.249), p = 0.041
129	CD56^{+}CD8^{+}CD11c^{+}CD25$^{-\text{ or }-/lo}$DAF-FM^{+}	CD8^{+} NK	16 to 1593	***Wilc.3,*** Mean = 0.339, p = 0.012
142	CD56^{+}CD8^{+}CD11c^{+}DAF-FM^{+}	CD8^{+} NK	4 to 1505	***Wilc.3,*** Mean = 0.306, p = 0.027
17	CD56^{+}CD8$^{+/lo}$CD11c^{+}CD25$^{-/lo}$DAF-FM^{+}	CD8$^{+/lo}$ NK	31 to 2860	***Wilc.3,*** Mean = 0.233, p = 0.042<br>***Cox.4,*** exp(coef) = 1.528, p = 0.032
118	CD56^{+}CD8$^{+/lo}$CD11c^{+} DAF-FM$^{-/lo}$	CD8$^{+/lo}$ NK	7 to 3856	***Wilc.3,*** Mean = 0.289, p = 0.048
23	CD3^{+}CD8^{+}DAF-FM$^{-/lo}$	Effector T Cell	59 to 2405	***Wilc.3,*** Mean = -0.343, p = 0.010
47	CD3^{+}CD8^{+}CD127$^{-\text{ or }-/lo}$CD25$^{-\text{ or }-/lo}$DAF-FM$^{-/lo}$	Effector T Cell	36 to 2730	***Wilc.3,*** Mean = -0.337, p = 0.033
133	CD3^{+}CD8^{+}CD127$^{-\text{ or }-/lo}$DAF-FM$^{-/lo}$	Effector T Cell	29 to 2135	***Wilc.3,*** Mean = -0.339, p = 0.030
188	CD3^{+}CD8^{+}CD127$^{-\text{ or }-/lo}$CD25$^{-\text{ or }-/lo}$DAF-FM$^{-/lo}$	Effector T Cell	15 to 1536	***Wilc.3,*** Mean = -0.269, p = 0.023
36	CD56^{+}CD8$^{-\text{ or }-/lo}$CD11c^{+} CD127$^{+/lo}$CD25$^{-\text{ or }-/lo}$DAF-FM^{+}	NK Cell	10 to 1239	***Wilc.3,*** Mean = 0.242, p = 0.040
94	CD56^{+}CD8$^{-/lo\text{ or }+/lo}$CD11c^{+}DAF-FM^{+}	NK Cell	11 to 1218	***Cox.4,*** exp(coef) = 1.511, p = 0.043
128	CD56^{+}CD8$^{-\text{ or }-/lo}$CD11c^{+} CD127$^{-/lo}$CD25$^{-/lo}$DAF-FM$^{-/lo\text{ or }+/lo}$	NK Cell	0 to 436	***Wilc.3,*** Mean = 0.404, p = 0.014

Table S4. Lymphoid FSC/SSC cell phenotypes (nodes) associated with treatment effects from the MPATR analysis

Node	Phenotype	Cell type	Cell Range	Statistics
148	CD56^{+}CD11c^{+}CD25$^{-\text{ or }-/lo}$DAF-FM^{+}	NK Cell	10 to 990	***Cox.4,*** exp(coef) = 1.678, p = 0.0496<br>***Wilc.4,*** RFS(<1yr, median = 0.143),<br>RFS(>1yr, median = -0.332), p = 0.019
8	CD3^{+}CD56$^{+/lo}$CD11c$^{-/lo}$CD25$^{-/lo\text{ or }+/lo}$DAF-FM$^{-/lo}$	NK-like Cell	1 to 1311	***Cox.4,*** exp(coef) = 0.584, p = 0.047
43	CD3^{+}CD8^{+}CD25$^{-/lo}$CD11c$^{-/lo\text{ or }+/lo}$CD56$^{-/lo}$CD127^{+}DAF-FM^{+}	NK-like Cell	0 to 248	***Wilc.1,*** RFS(<1yr, mean = -10.565, SD = 0.430),<br>RFS(>1yr, mean = -10.989, SD = 0.549), p = 0.038
2	CD3^{+}CD4$^{+/lo}$CD25$^{+/lo}$CD127$^{-/lo}$DAF-FM$^{-/lo}$	T Regulatory Cell	28 to 3082	***Cox.1,*** exp(coef) = 2.146, p = 0.039<br>***Wilc.3*** **,** Mean = -0.419, p = 0.017<br>***Cox.4,*** exp(coef) = 0.547, p = 0.033
160	CD3^{+}CD19^{+}CD4$^{+/lo}$CD11c$^{-/lo}$CD127^{+}DAF-FM^{+}	Artifact	2 to 136	***Wilc.3,*** Mean = -0.455, p = 0.025
4	CD11c$^{+/lo}$CD25$^{+/lo}$DAF-FM^{+}	-	1 to 302	***Wilc.2*** **,** RFS(<1yr, mean = -11.227, SD = 0.982),<br>RFS(>1yr, mean = -12.143, SD = 1.063), p = 0.034<br>***Wilc.3,*** Mean = 0.452, p = 0.042<br>***Cox.4,*** exp(coef) = 1.661, p = 0.032<br>***Wilc.4,*** RFS(<1yr, median = 0.372),<br>RFS(>1yr, median = -0.607), p = 0.005
18	CD3$^{-/lo}$CD4$^{-/lo}$CD11c^{+}CD25$^{+/lo}$CD56$^{-/lo}$DAF-FM$^{-/lo}$	-	4 to 1518	***Cox.1,*** exp(coef) = 1.936, p = 0.041<br>***Wilc.1,*** RFS(<1yr, mean = -9.112, SD = 0.779),<br>RFS(>1yr, mean = -9.634, SD = 0.723), p = 0.044<br>***Wilc.3*** **,** Mean = 0.400, p = 0.012<br>***Cox.4,*** exp(coef) = 0.405, p = 0.008<br>***Wilc.4,*** RFS(<1yr, median = -1.091),<br>RFS(>1yr, median = -0.338), p = 0.037
21	CD11c^{+}CD25$^{-/lo\text{ or }+/lo}$DAF-FM$^{+/lo}$	-	87 to 10155	***Cox.4,*** exp(coef) = 0.468, p = 0.035<br>***Wilc.4,*** RFS(<1yr, median = -0.878),<br>RFS(>1yr, median = -0.070), p = 0.0457
24	CD11c^{+}CD56$^{-/lo}$CD25$^{+/lo}$DAF-FM$^{-/lo\text{ or }+/lo}$	-	3 to 1893	***Wilc.3,*** Mean = 0.367, p = 0.028
27	CD11c$^{-/lo}$CD25$^{-\text{ or }-/lo}$DAF-FM^{+}	-	17 to 388	***Wilc.3,*** Mean = 0.250, p = 0.025
28	CD11c^{+}CD25^{+}CD56$^{-\text{ or }-/lo}$DAF-FM^{+}	-	10 to 10882	***Wilc.3,*** Mean = 0.346, p = 0.049
31	CD11c$^{-/lo\text{ or }+/lo}$CD25$^{-/lo}$DAF-FM$^{-/lo\text{ or }+/lo}$	-	3 to 1185	***Wilc.4*** **,** RFS(<1yr, median = 0.540),<br>RFS(>1yr, median = -0.491), p = 0.048
50	CD11c^{+}CD56$^{-\text{ or }-/lo}$CD8$^{-\text{ or }-/lo}$CD25$^{+/lo}$DAF-FM^{+}	-	0 to 9911	***Wilc.3,*** Mean = 0.437, p = 0.046
51	CD11c^{+}CD25$^{+/lo}$CD4$^{-\text{ or }-/lo}$DAF-FM^{+}	-	10 to 3509	***Wilc.3,*** Mean = 0.411, p = 0.014
55	CD11c^{+}CD25^{+}CD4$^{-\text{ or }-/lo}$DAF-FM^{+}	-	8 to 3024	***Wilc.3,*** Mean = 0.422, p = 0.011
58	CD11c^{+}CD25^{+}CD56$^{-\text{ or }-/lo}$CD4$^{-\text{ or }-/lo}$DAF-FM$^{+/lo}$	-	21 to 1379	***Wilc.3,*** Mean = 0.253, p = 0.034
62	CD11c^{+}CD25$^{+/lo}$CD4$^{-\text{ or }-/lo}$DAF-FM^{+}	-	0 to 1618	***Wilc.1,*** RFS(<1yr, mean = -12.077, SD = 0.638),<br>RFS(>1yr, mean = -11.538, SD = 0.781), p = 0.033
103	CD11c$^{-/lo}$CD25$^{-\text{ or }-/lo}$DAF-FM$^{+/lo}$	-	2 to 2042	***Wilc.3,*** Mean = 0.708, p = 0.036
156	CD11c^{+}CD25$^{+/lo}$CD56$^{-\text{ or }-/lo}$CD8$^{-\text{ or }-/lo}$DAF-FM^{+}	-	16 to 5428	***Wilc.3,*** Mean = 0.328, p = 0.044

Table S4. Lymphoid FSC/SSC cell phenotypes (nodes) associated with treatment effects from the MPATR analysis				
**Node**	**Phenotype**	**Cell type**	**Cell Range**	**Statistics**
170	$CD11c^{+}CD25^{+/lo}DAF\text{-}FM^{-/lo}$	-	13 to 2930	***Cox.4,*** exp(coef) = 0.468, p = 0.015
182	$CD11c^{+}CD25^{-/lo}DAF\text{-}FM^{-/lo\ or\ +/lo}$	-	2 to 761	***Wilc.3,*** *Mean =* 0.334, p = 0.031
191	$CD11c^{+}CD25^{-/lo}DAF\text{-}FM^{+/lo}$	-	2 to 2451	***Cox.4,*** exp(coef) = 0.309, p = 0.015<br>***Wilc.4,*** RFS(<1yr, median = -0.925),<br>RFS(>1yr, median = -0.033), p = 0.013

Table S5. Phenotypic characteristics of myeloid immune cells (nodes) associated with treatment effects from the MPATR output.

Table S5. Myeloid cell phenotypes (nodes) associated with treatment effects from the MPATR analysis

Node	Phenotype	Cell Type	Cell Range	Statistics
47	HLA-DR^{+}CD33$^{-/lo \text{ or } +/lo}$CD11b^{+}CD11c^{+}CD14$^{-/lo}$DAF-FM$^{-/lo}$	Dendritic Cell	1 to 1709	***Cox.1*** , exp(coef) = 1.882, p = 0.037<br>***Cox.4*** , exp(coef) = 0.599, p = 0.036
49	HLA-DR^{+}CD33^{+}CD11b^{+}CD11c^{+}DAF-FM^{+}	Dendritic Cell	0 to 640	***Wilc.3,*** Mean = 0.510, p = 0.004
108	HLA-DR$^{+/lo}$CD33$^{-/lo \text{ or } +/lo}$CD11b^{+}CD11c^{+}DAF-FM^{+}	Dendritic Cell	0 to 1211	***Wilc.3,*** Mean = 0.370, p = 0.022<br>***Wilc.4,*** RFS(<1yr, median = 0.201),<br>RFS(>1yr, median = -0.654), p = 0.046
125	HLA-DR^{+}CD33$^{+/lo}$ CD11b$^{+/lo}$CD11c^{+}DAF-FM$^{+/lo}$	Dendritic Cell	17 to 2449	***Cox.4,*** exp(coef) = 0.445, p = 0.021<br>***Wilc.4,*** RFS(<1yr, median = -1.244),<br>RFS(>1yr, median = -0.235), p = 0.024
129	HLA-DR$^{+/lo}$CD33$^{+/lo}$ CD11b^{+}CD11c^{+}DAF-FM^{+}	Dendritic Cell	0 to 878	***Cox.4,*** exp(coef) = 1.656, p = 0.029<br>***Wilc.4,*** RFS(<1yr, median = 0.604),<br>RFS(>1yr, median = -0.861), p = 0.012
150	HLA-DR^{+}CD33$^{-/lo \text{ or } +/lo}$ CD11b^{+}CD11c^{+}CD14$^{-/lo \text{ or } +/lo}$DAF-FM^{+}	Dendritic Cell	14 to 598	***Cox.4,*** exp(coef) =0.388, p = 0.045
153	HLA-DR$^{+/lo}$CD33$^{-/lo \text{ or } +/lo}$CD11b^{+}CD11c^{+} DAF-FM$^{-/lo \text{ or } +/lo}$	Dendritic Cell	1 to1463	***Cox.4,*** exp(coef) =0.452, p = 0.038
178	HLA-DR^{+}CD33^{+}CD11b^{+}CD11c^{+}CD14$^{-/lo}$DAF-FM$^{+/lo}$	Dendritic Cell	1 to 2985	***Cox.4,*** exp(coef) =0.360, p = 0.016
14	HLA-DR^{+}CD33$^{-/lo \text{ or } +/lo}$CD11b^{+}CD11c^{+} CD14$^{-/lo}$DAF-FM^{+}	Monocytic	3 to 1065	***Cox.1*** **,**exp(coef) = 3.592, p = 0.013<br>**Wilc.1,** RFS(<1yr, mean = -9.772, SD = 0.615),<br>RFS(>1yr, mean = -10.328, SD = 0.501), p = 0.014
18	HLA-DR^{+}CD33^{+}CD11b^{+}CD11c^{+} CD14^{+}DAF-FM^{+}	Monocytic	12 to 3609	***Cox.4*** , exp(coef) = 0.610, p = 0.023
32	HLA-DR^{+}CD33^{+}CD11b^{+}CD11c^{+} CD14^{+}DAF-FM$^{+/lo}$	Monocytic	1 to 2449	***Cox.4*** , exp(coef) = 0.492, p = 0.022
66	HLA-DR^{+}CD33^{+}CD11b^{+}CD11c^{+}CD14^{+}DAF-FM^{+}	Monocytic	24 to 4261	***Wilc.3,*** Mean = 0.440, p = 0.025
78	HLA-DR^{+}CD33^{+}CD11b^{+}CD11c^{+}CD14$^{+/lo}$DAF-FM$^{+/lo}$	Monocytic	0 to 2619	***Wilc.1*** , RFS(<1yr, mean = -9.348, SD =0.851),<br>RFS(>1yr, mean = -10.140, SD = 1.179), p = 0.033<br>***Cox.4*** , exp(coef) = 0.643, p = 0.029<br>***Wilc.4*** , RFS(<1yr, median = -1.289),<br>RFS(>1yr, median = -0.243), p = 0.037
134	HLA-DR^{+}CD33^{+}CD11b^{+}CD11c^{+}CD14^{+}DAF-FM$^{+ \text{ or } +/lo}$	Monocytic	2 to 3005	***Wilc.3,*** Mean = 0.456, p = 0.012
137	HLA-DR^{+}CD33^{+}CD11b^{+}CD11c^{+}CD14^{+}DAF-FM$^{+/lo}$	Monocytic	50 to 1595	***Cox.1,*** exp(coef) = 1.873, p = 0.039<br>***Wilc.1,*** RFS(<1yr, mean = -9.877, SD =0.992),<br>RFS(>1yr, mean = -10.522, SD = 0.778), p = 0.038<br>***Cox.4,*** exp(coef) =0.492, p = 0.009<br>***Wilc.4,*** RFS(<1yr, median = -0.618),<br>RFS(>1yr, median = -0.011), p = 0.041
167	HLA-DR^{+}CD33^{+}CD11b^{+}CD11c^{+}CD14^{+}DAF-FM^{+}	Monocytic	19 to 3956	***Wilc.1,*** RFS(<1yr, mean = -8.394, SD =1.168),<br>RFS(>1yr, mean = -9.340, SD = 1.125), p = 0.028<br>***Cox.4,*** exp(coef) = 0.488, p = 0.021
155	HLA-DR^{+}CD33$^{+/lo}$CD11b$^{-/lo}$CD11c^{+}DAF-FM$^{+/lo}$	Plasmacytoid DC (CD11b$^{-/lo}$)	4 to 786	***Cox.4,*** exp(coef) =0.450 p = 0.021<br>**Wilc.4**, RFS(<1yr, median = -0.787),<br>RFS(>1yr, median = -0.050), p = 0.019
185	HLA-DR^{+}CD33^{+}CD11c^{+}DAF-FM^{+}	Plasmacytoid DC (CD11b^{-})	1 to 759	***Wilc.1,*** RFS(<1yr, mean = -12.297, SD =0.856),<br>RFS(>1yr, mean = -12.879, SD = 0.821), p = 0.047
1	HLA-DR^{+}DAF-FM^{+}	-	6 to 2076	***Wilc.3,*** Mean = -0.487, p = 0.018
10	CD11b^{+}CD11c^{+}HLA-DR$^{-/lo}$DAF-FM$^{-/lo}$	-	0 to 881	***Wilc.3,*** Mean = 0.315 , p = 0.042

Table S5. Myeloid cell phenotypes (nodes) associated with treatment effects from the MPATR analysis				
**Node**	**Phenotype**	**Cell Type**	**Cell Range**	**Test**
11	HLA-DR$^{-/\mathrm{lo}\ \mathrm{or}\ -}$DAF-FM^{+}	-	113 to 18203	***Cox.4,*** exp(coef) = 2.168, p = 0.016
13	CD11b$^{+/\mathrm{lo}}$HLA-DR$^{+/\mathrm{lo}}$DAF-FM^{+}	-	45 to 1623	***Wilc.3,*** Mean = -0.465, p = 0.003
24	CD11b$^{-\ \mathrm{or}\ -/\mathrm{lo}}$HLA-DR$^{-\ \mathrm{or}\ -/\mathrm{lo}}$DAF-FM$^{-\ \mathrm{or}\ -/\mathrm{lo}}$	-	115 to 11180	***Cox.1*** , exp(coef) = 2.220, p = 0.013
27	HLA-DR$^{-/\mathrm{lo}\ \mathrm{or}\ +/\mathrm{lo}}$DAF-FM^{+}	-	121 to 3260	***Wilc.3,*** Mean = -0.374, p = 0.034
35	CD11b^{+}CD11c^{+}HLA-DR$^{-\ \mathrm{or}\ -/\mathrm{lo}}$DAF-FM^{+}	-	40 to 4978	***Wilc.3*** , Mean = 0.295, p = 0.034<br>***Cox.4*** , exp(coef) =1.484, p = 0.036<br>***Wilc.4*** , RFS(<1yr, median = -0.045),<br>RFS(>1yr, median = -0.607), p = 0.030
36	CD11b^{+}CD11c^{+}HLA-DR$^{-/\mathrm{lo}\ \mathrm{or}\ +/\mathrm{lo}}$DAF-FM^{+}	-	1 to 5539	***Wilc.3,*** Mean = 0.476, p = 0.020
37	CD11b$^{-\ \mathrm{or}\ -/\mathrm{lo}}$CD11c$^{-\ \mathrm{or}\ -/\mathrm{lo}}$DAF-FM^{+}	-	53 to 22078	***Cox.4,*** exp(coef) = 2.232, p = 0.017
40	CD11b$^{-/\mathrm{lo}}$CD11c$^{-/\mathrm{lo}}$HLA-DR^{+}DAF-FM$^{-/\mathrm{lo}}$	-	22 to 1960	***Cox.1,*** exp(coef) = 2.468, p = 0.011<br>***Cox.4*** , exp(coef) = 0.502, p = 0.138
41	CD11b^{+}CD11c$^{+/\mathrm{lo}}$HLA-DR$^{+/\mathrm{lo}}$DAF-FM^{+}	-	26 to 2742	***Wilc.2*** , RFS(<1yr, mean = -7.845, SD =0.456),<br>RFS(>1yr, mean = -7.385, SD = 0.513), p = 0.019
43	DAF-FM$^{-/\mathrm{lo}}$	-	97 to 5628	***Cox.1 ,***exp(coef) = 2.538, p = 0.015
45	DAF-FM^{+}	-	109 to 29376	***Cox.4,*** exp(coef) =2.391, p = 0.006
46	CD11b$^{-/\mathrm{lo}}$HLA-DR$^{+/\mathrm{lo}}$DAF-FM^{+}	-	110 to 5738	***Wilc.3,*** Mean = -0.527, p = 0.003
48	HLA-DR$^{+\ \mathrm{or}\ +/\mathrm{lo}}$DAF-FM^{+}	-	19 to 2039	***Wilc.3,*** Mean = -0.508, p = 0.011
52	CD11b^{+}CD11c$^{-/\mathrm{lo}\ \mathrm{or}\ +/\mathrm{lo}}$CD33$^{-/\mathrm{lo}}$HLA-DR^{+}DAF-FM$^{-/\mathrm{lo}\ \mathrm{or}\ +/\mathrm{lo}}$	-	6 to 4031	***Cox.2*** , exp(coef) = 0.204, p = 0.022<br>***Wilc.2*** , RFS(<1yr, mean = -8.951, SD =0.669),<br>RFS(>1yr, mean = -8.416, SD = 0.634), p = 0.017
59	CD11c$^{-\ \mathrm{or}\ -/\mathrm{lo}}$HLA-DR$^{-\ \mathrm{or}\ -/\mathrm{lo}}$DAF-FM$^{-/\mathrm{lo}}$	-	179 to 12508	***Cox.1 ,***exp(coef) = 2.769, p = 0.014
61	CD11c$^{-\ \mathrm{or}\ -/\mathrm{lo}}$HLA-DR$^{-/\mathrm{lo}\ \mathrm{or}\ +/\mathrm{lo}}$DAF-FM$^{+/\mathrm{lo}}$	-	22 to 1774	***Wilc.3,*** Mean = -0.442, p = 0.004
67	CD11b^{+}CD11c^{+}HLA-DR$^{-/\mathrm{lo}}$DAF-FM^{+}	-	25 to 7372	***Cox.4*** , exp(coef) = 1.549, p = 0.020
68	DAF-FM$^{-\ \mathrm{or}\ -/\mathrm{lo}}$	-	117 to 7657	***Cox.1 ,***exp(coef) = 2.281, p = 0.026
70	CD11b^{+}HLA-DR$^{+\ \mathrm{or}\ +/\mathrm{lo}}$DAF-FM$^{+\ \mathrm{or}\ +/\mathrm{lo}}$	-	4 to 2338	***Wilc.2*** , RFS(<1yr, mean = -9.629, SD =0.921),<br>RFS(>1yr, mean = -8.917, SD = 0.540), p = 0.0373
75	CD11b$^{-\ \mathrm{or}\ -/\mathrm{lo}}$HLA-DR^{+}DAF-FM$^{-\ \mathrm{or}\ -/\mathrm{lo}}$	-	49 to 5177	***Wilc.3,*** Mean = -0.501, p = 0.008

Table S5. Myeloid cell phenotypes (nodes) associated with treatment effects from the MPATR analysis				
**Node**	**Phenotype**	**Cell Type**	**Cell Range**	**Test**
76	DAF-FM^{+}	-	434 to 11915	***Cox.4,*** exp(coef) = 1.915, p = 0.037
80	CD11b$^{-/lo\ or\ +/lo}$HLA-DR^{+}DAF-FM$^{+/lo}$	-	8 to 1476	***Wilc.3,*** Mean = -0.345, p = 0.044
85	HLA-DR^{+}DAF-FM$^{+/lo}$	-	23 to 908	***Cox.4,*** exp(coef) = 0.543, p = 0.029
86	CD11b^{+}CD11c^{+}CD33$^{-/lo}$HLA-DR$^{+/lo}$DAF-FM$^{-/lo}$	-	0 to 1375	***Cox.4,*** exp(coef) = 0.425, p = 0.027
89	CD11b$^{-/lo}$HLA-DR^{+}DAF-FM$^{-/lo}$	-	14 to 2018	***Cox.1,*** exp(coef) = 1.918, p = 0.037<br>***Cox.4,*** exp(coef) = 0.636, p = 0.042
90	CD11b^{+}CD11c^{+}DAF-FM^{+}	-	0 to 2635	***Wilc.3,*** Mean = 0.379, p = 0.049<br>***Cox.4,*** exp(coef) = 1.571, p = 0.034
95	HLA-DR$^{-/lo\ or\ +/lo}$DAF-FM$^{-/lo}$	-	52 to 3216	***Cox.1,*** exp(coef) = 2.029, p = 0.028
96	HLA-DR^{+}DAF-FM$^{-/lo}$	-	51 to 3763	***Cox.4,*** exp(coef) = 0.593, p = 0.022
105	CD11b$^{-/lo\ or\ +/lo}$CD11c$^{-/lo}$HLA-DR^{+}DAF-FM$^{-/lo}$	-	3 to 2259	***Cox.4,*** exp(coef) = 0.643, p = 0.033
112	DAF-FM^{+}	-	11 to 9443	***Cox.4,*** exp(coef) = 2.213, p = 0.034
114	CD11b^{+}CD11c^{+}DAF-FM$^{-/lo}$	-	4 to 3092	***Wilc.3,*** Mean = 0.316, p = 0.017
120	HLA-DR$^{-\ or\ -/lo}$DAF-FM$^{-/lo}$	-	103 to 6237	***Cox.1*** ,exp(coef) = 2.208, p = 0.013
121	CD11c$^{-/lo}$HLA-DR$^{-/lo}$DAF-FM^{+}	-	98 to 2445	***Cox.1,*** exp(coef) = 2.694, p = 0.041
122	CD11b$^{+\ or\ +/lo}$DAF-FM^{+}	-	1 to 4039	***Cox.4,*** exp(coef) = 1.944, p = 0.029
123	CD11b$^{-/lo}$CD11c$^{-/lo}$HLA-DR^{+}DAF-FM^{+}	-	85 to 2701	***Wilc.3,*** Mean = -0.334, p = 0.030<br>***Cox.4,*** exp(coef) = 0.572, p = 0.038
133	CD11c$^{-/lo}$HLA-DR^{+}DAF-FM$^{-/lo\ or\ +/lo}$	-	2 to 693	***Cox.4,*** exp(coef) = 0.610, p = 0.028
142	CD11b^{+}CD11c^{+}HLA-DR$^{-/lo}$DAF-FM$^{+/lo}$	-	0 to 778	***Wilc.3,*** Mean = 0.282, p = 0.024
160	CD11b^{+}CD11c^{+}CD33$^{-/lo}$HLA-DR^{+}DAF-FM$^{-/lo\ or\ +/lo}$	-	7 to 2355	***Wilc.2,*** RFS(<1yr, mean = -9.984, SD =0.359), RFS(>1yr, mean = -9.335, SD = 0.831), p = 0.022<br>***Cox.4,*** exp(coef) =0.548 p = 0.034
162	HLA-DR$^{+/lo}$DAF-FM$^{+/hi}$	-	1 to 1250	***Wilc.3,*** Mean = -0.461, p = 0.007
175	CD11b$^{-/lo}$HLA-DR^{+}DAF-FM^{+hi}	-	3 to 1817	***Wilc.3,*** Mean = -0.404, p = 0.049

Table S5. Myeloid cell phenotypes (nodes) associated with treatment effects from the MPATR analysis				
**Node**	**Phenotype**	**Cell Type**	**Cell Range**	**Test**
177	CD11c$^{-/lo}$HLA-DR^{+}DAF-FM$^{-/lo\text{ or }+/lo}$	-	26 to 2852	***Cox.4,*** exp(coef) = 0.540, p = 0.013
179	CD11b$^{-/lo\text{ or }+/lo}$CD11c$^{-/lo\text{ or }+/lo}$CD33$^{-/lo}$HLA-DR$^{-/lo\text{ or }+/lo}$DAF-FM$^{-/lo}$	-	17 to 1863	***Cox.1,*** exp(coef) = 2.539, p = 0.036<br>***Cox.4,*** exp(coef) = 0.515, p = 0.045
186	HLA-DR^{+}DAF-FM^{+}	-	46 to 1226	***Wilc.3,*** Mean = -0.291, p = 0.040
189	CD11b$^{-/lo\text{ or }+/lo}$CD11c$^{+/lo}$HLA-DR^{+}DAF-FM$^{+\text{ or }+/lo}$	-	3 to 2555	***Cox.1,*** exp(coef) = 2.779 , p = 0.016<br>***Cox.4,*** exp(coef) = 0.256, p = 0.005
190	CD11b^{+}HLA-DR^{+}DAF-FM$^{-/lo\text{ or }+/lo}$	-	5 to 455	***Cox.2,*** exp(coef) = 0.347, p = 0.028<br>***Wilc.2*** , RFS(<1yr, mean = -11.425, SD =0.612),<br>RFS(>1yr, mean = -10.904, SD = 0.638), p = 0.046<br>***Cox.4,*** exp(coef) = 0.527, p = 0.020
193	HLA-DR^{+}DAF-FM^{+}	-	12 to 2591	***Wilc.3,*** Mean = -0.446, p = 0.018
195	CD11c^{+}CD11b$^{+/lo}$HLA-DR^{+}DAF-FM$^{-/lo}$	-	3 to 1693	***Cox.1,*** exp(coef) = 1.821, p = 0.034<br>***Cox.4,*** exp(coef) = 0.489, p = 0.004
198	CD11c^{+}CD33$^{-/lo}$HLA-DR^{+}DAF-FM$^{-/lo}$	-	0 to 380	***Cox.4,*** exp(coef) = 0.562, p = 0.033
111	CD11b^{+}CD11c$^{+/lo}$CD33$^{-/lo\text{ or }+/lo}$HLA-DR$^{+\text{ or }+/lo}$DAF-FM$^{-/lo}$	-	1 to 1145	***Cox.4,*** exp(coef) = 0.420, p = 0.023
64	CD11b^{+}CD11c^{+}CD33$^{-/lo}$HLA-DR^{+}DAF-FM$^{-/lo\text{ or }+/lo}$	-	0 to 2035	***Cox.4*** , exp(coef) = 0.439, p = 0.017

Table S6. Phenotypic characteristics myeloid immune cells (nodes) with the addition of scatter properties FSC and SSC associated with treatment effects from the MPATR output.

Table S6. Myeloid FSC/SSC cell phenotypes (nodes) associated with treatment effects from the MPATR analysis				
**Node**	**Phenotype**	**Cell Type**	**Cell Range**	**Test**
3	HLA-DR^{+}CD33$^{+/lo}$CD11b^{+}CD11c^{+}CD14$^{-/lo}$DAF-FM^{+}	Dendritic Cell	1 to 1010	***Wilc.1*** , RFS(<1yr, mean = -10.328, SD = 0.823),<br>RFS(>1yr, mean = -10.874, SD = 0.581), p = 0.038
20	HLA-DR^{+}CD33^{+}CD11b$^{+/lo}$CD11c^{+}CD14$^{-\ or\ -/lo}$DAF-FM$^{+/lo}$	Dendritic Cell	1 to 1844	**Cox.4**, exp(coef) = 0.378, p = 0.018
38	HLA-DR^{+hi}CD33^{+} CD11b^{+}CD11c^{+}DAF-FM^{+}	Dendritic Cell	0 to 441	***Wilc.3*** , Mean = 0.625, p = 0.001
69	HLA-DR^{+}CD33$^{+/lo}$ CD11b^{+}CD11c^{+hi}DAF-FM^{+}	Dendritic Cell	9 to 1955	***Cox.4,*** exp(coef) = 0.429, p = 0.044
90	HLA-DR^{+}CD33^{+}CD11b$^{-/lo\ or\ +/lo}$CD11c^{+}DAF-FM^{+}	Dendritic Cell	0 to 295	***Wilc.3*** , Mean =0.363, p = 0.021
94	HLA-DR^{+}CD33$^{+/lo}$CD11b^{+}CD11c^{+}CD14$^{-\ or\ -/lo}$DAF-FM$^{-/lo}$	Dendritic Cell	1 to 868	***Cox.4,*** exp(coef) = 0.501, p = 0.039
143	HLA-DR^{+}CD33$^{+/lo}$CD11b^{+}CD11c^{+}CD14$^{-/lo}$DAF-FM^{+}	Dendritic Cell	1 to 2103	***Cox.4,*** exp(coef) = 0.371, p = 0.028
168	HLA-DR^{+}CD33^{+}CD11b^{+}CD11c^{+}DAF-FM^{+}	Dendritic Cell	0 to 596	***Wilc.3*** , Mean = 0.490, p = 0.029
196	HLA-DR$^{-/lo\ or\ +/lo}$CD33$^{-/lo\ or\ +/lo}$CD11b^{+}CD11c^{+} DAF-FM^{+}	MDSC-like	4 to 746	***Wilc.3,*** Mean = 0.498, p = 0.004<br>***Wilc.4,*** RFS(<1yr, median = 0.369),<br>RFS(>1yr, median = -0.785), p = 0.012
14	HLA-DR^{+}CD33^{+}CD11b^{+}CD11c^{+}CD14^{+}DAF-FM$^{+/lo}$	Monocytic	3 to 1575	***Cox.4*** , exp(coef) = 0.538, p = 0.035
16	HLA-DR^{+}CD33^{+}CD11b^{+}CD11c^{+}CD14^{+}DAF-FM$^{-/lo\ or\ +/lo}$	Monocytic	0 to 1796	***Cox.4*** , exp(coef) = 0.548, p = 0.017
17	HLA-DR^{+}CD33^{+}CD11b^{+}CD11c^{+}CD14$^{+/lo}$DAF-FM$^{+/lo}$	Monocytic	1 to 1338	***Cox.1*** , exp(coef) = 1.976, p = 0.025<br>***Wilc.1*** , RFS(<1yr, mean = -9.553, SD =0.788),<br>RFS(>1yr, mean = -10.354, SD = 0.757), p = 0.067<br>***Cox.4,*** exp(coef) = 0.329, p = 0.002<br>***Wilc.4,*** RFS(<1yr, median = -0.830),<br>RFS(>1yr, median = -0.138), p = 0.005
43	HLA-DR^{+}CD33^{+}CD11b^{+}CD11c^{+}CD14^{+}DAF-FM^{+}	Monocytic	2 to 3498	***Wilc.1,*** RFS(<1yr, mean = -8.155, SD =0.850),<br>RFS(>1yr, mean = -8.916, SD = 0.773), p = 0.026<br>***Cox.4*** , exp(coef) = 0.488 , p = 0.019<br>***Wilc.4,*** RFS(<1yr, median = -0.755),<br>RFS(>1yr, median = -0.131), p = 0.037
44	HLA-DR^{+}CD33^{+}CD11b^{+}CD11c^{+}CD14^{+}DAF-FM^{+}	Monocytic	3 to 2741	***Wilc.3,*** Mean = 0.684, p = 0.005
65	HLA-DR^{+}CD33^{+}CD11b^{+}CD11c^{+}CD14$^{+/lo}$CD15$^{-/lo}$DAF-FM^{+}	Monocytic	40 to 8258	***Wilc.3*** , Mean = 0.349, p = 0.027
102	HLA-DR^{+}CD33^{+}CD11b^{+}CD11c^{+}CD14^{+}DAF-FM$^{+/lo}$	Monocytic	0 to 3800	***Cox.4,*** exp(coef) = 0.462, p = 0.002<br>***Wilc.4,*** RFS(<1yr, median = -0.975),<br>RFS(>1yr, median = -0.087), p = 0.013
106	HLA-DR^{+}CD33^{+}CD11b^{+}CD11c^{+}CD14^{+}DAF-FM^{+}	Monocytic	3 to 8020	***Cox.4,*** exp(coef) = 0.620, p = 0.040
111	HLA-DR^{+}CD33^{+}CD11b^{+}CD11c^{+}CD14^{+}CD15$^{-/lo}$DAF-FM^{+}	Monocytic	11 to 10220	***Wilc.3*** , Mean = 0.484, p = 0.013
185	HLA-DR^{+}CD33^{+}CD11b^{+}CD11c^{+}CD14^{+}DAF-FM$^{-/lo\ or\ +/lo}$	Monocytic	0 to 1169	***Wilc.3*** , Mean = 0.522, p = 0.042
189	HLA-DR^{+}CD33^{+}CD11b^{+}CD11c^{+}CD14$^{-/lo\ or\ +/lo}$DAF-FM^{+}	Monocytic	0 to 902	***Wilc.3*** , Mean = 0.441, p = 0.024

Table S6. Myeloid FSC/SSC cell phenotypes (nodes) associated with treatment effects from the MPATR analysis				
**Node**	**Phenotype**	**Cell Type**	**Cell Range**	**Test**
198	HLA-DR^{+}CD33^{+}CD11b^{+}CD11c^{+}CD14^{+}CD15$^{+/lo}$DAF-FM^{+}	Monocytic	0 to 21415	***Wilc.3*** , Mean = 0.527, p = 0.037
5	DAF-FM^{+}	-	326 to 14255	***Cox.4*** , exp(coef) = 2.090, p = 0.168
7	CD11b^{+}CD11c^{+}HLA-DR$^{-/lo\text{ or }+/lo}$DAF-FM^{+}	-	0 to 6811	***Cox.4,*** exp(coef) = 1.540, p = 0.042<br>***Wilc.4,*** RFS(<1yr, median = 0.982),<br>RFS(>1yr, median = -0.531), p = 0.009
10	CD11b$^{+/lo}$CD11c$^{+/lo}$HLA-DR^{+}DAF-FM$^{-/lo\text{ or }+/lo}$	-	3 to 5397	***Cox.1*** , exp(coef) = 1.900, p = 0.046<br>***Cox.4*** , exp(coef) = 0.577, p = 0.041
15	CD11b$^{-\text{ or }-/lo}$HLA-DR^{+}DAF-FM$^{-/lo\text{ or }+/lo}$	-	28 to 2345	***Cox.4*** , exp(coef) = 0.621, p = 0.038
21	HLA-DR^{+}DAF-FM^{+}	-	56 to 3732	***Wilc.3,*** Mean = -0.469, p = 0.018
22	CD11b^{+}CD11c^{+}HLA-DR$^{-\text{ or }-/lo}$DAF-FM$^{-/lo\text{ or }+/lo}$	-	4 to 2809	***Wilc.3,*** Mean = 0.287, p = 0.018
27	HLA-DR^{+}DAF$^{-/lo}$	-	77 to 4138	***Wilc.3,*** Mean = -0.455, p = 0.031
28	HLA-DR$^{-\text{ or }-/lo}$DAF-FM$^{-/lo}$	-	134 to 11719	**Cox.1**, exp(coef) = 2.177 , p = 0.014
29	HLA-DR$^{-/lo}$DAF-FM^{+}	-	53 to 20362	**Cox.4**, exp(coef) = 2.183 , p = 0.013
30	CD11b$^{-/lo\text{ or }+/lo}$HLA-DR$^{+/lo}$DAF-FM^{+}	-	87 to 2932	***Wilc.3,*** Mean = -0.395, p = 0.001
32	HLA-DR^{+}DAF-FM$^{-/lo}$	-	67 to 2112	**Cox.4**, exp(coef) = 0.566 , p = 0.028
37	CD11b^{+}CD11c^{+}HLA-DR$^{-/lo}$DAF-FM^{+}	-	0 to 5884	**Cox.4**, exp(coef) = 1.602, p = 0.029
46	CD11b^{+}CD11c^{+}HLA-DR$^{-/lo}$DAF-FM^{+}	-	0 to 2048	***Cox.2*** , exp(coef) = 2.457, p = 0.017<br>***Wilc.2,*** RFS(<1yr, mean = -11.104, SD = 0.723),<br>RFS(>1yr, mean = -12.118, SD = 1.021), p = 0.012<br>***Wilc.3,*** Mean = 0.401, p = 0.0495<br>***Cox.4*** , exp(coef) = 1.924 , p = 0.002<br>***Wilc.4*** , RFS(<1yr, median = 0.792),<br>RFS(>1yr, median = -0.674), p = 0.019
52	HLA-DR^{+}DAF-FM^{+}	-	58 to 3485	***Wilc.3*** , Mean = -0.511, p = 0.010
62	CD11b$^{-/lo}$HLA-DR^{+}DAF-FM$^{+/lo}$	-	59 to 995	***Cox.1,*** exp(coef) = 2.660, p = 0.020<br>***Wilc.3,*** Mean = -0.348, p = 0.008<br>***Cox.4,*** exp(coef) = 0.438, p = 0.004
68	CD11b^{+}CD11c^{+}HLA-DR$^{-/lo\text{ or }+/lo}$DAF-FM^{+}	-	44 to 7154	***Wilc.3*** , Mean = 0.330, p = 0.011
78	CD11b$^{+/lo}$CD11c$^{-/lo}$HLA-DR^{+}DAF-FM$^{-/lo}$	-	4 to 1691	***Cox.2,*** exp(coef) = 0.264, p = 0.019
82	CD11b$^{-/lo\text{ or }+/lo}$CD11c$^{-/lo}$HLA-DR^{+}DAF-FM^{+}	-	1 to 2770	***Wilc.1,*** RFS(<1yr, mean = -10.691, SD =0.939),<br>RFS(>1yr, mean = -10.251, SD = 0.724), p = 0.047<br>***Wilc.3,*** Mean = -0.421, p = 0.037
84	CD11b$^{-/lo}$CD11c$^{-/lo\text{ or }+/lo}$HLA-DR^{+}DAF-FM$^{-/lo}$	-	40 to 5758	***Cox.4,*** exp(coef) = 0.551, p = 0.017

Table S6. Myeloid FSC/SSC cell phenotypes (nodes) associated with treatment effects from the MPATR analysis				
**Node**	**Phenotype**	**Cell Type**	**Cell Range**	**Test**
89	$CD11b^{+}CD11c^{-/lo}HLA\text{-}DR^{+}DAF\text{-}FM^{+}$	-	39 to 10134	***Wilc.3***, Mean = -0.440, p = 0.0475
91	$CD11b^{-/lo}CD11c^{-/lo}HLA\text{-}DR^{-/lo}DAF\text{-}FM^{+}$	-	76 to 3274	***Wilc.3***, Mean = -0.206, p = 0.045
98	$CD11b^{+/lo}CD11c^{+}CD33^{-\ or\ -/lo}HLA\text{-}DR^{+}DAF\text{-}FM^{-/lo\ or\ +/lo}$	-	8 to 1986	***Cox.4,*** exp(coef) = 0.588, p = 0.026
101	$CD11b^{-\ or\ -/lo}CD11c^{-/lo\ or\ +/lo}HLA\text{-}DR^{+hi}DAF\text{-}FM^{-/lo\ or\ +/lo}$	-	1 to 738	***Cox.4,*** exp(coef) = 0.628, p = 0.036
105	$HLA\text{-}DR^{-/lo}DAF\text{-}FM^{-/lo}$	-	235 to 15831	***Cox.1,*** exp(coef) = 2.356, p = 0.029
107	$CD11b^{+}CD11c^{+}HLA\text{-}DR^{-/lo}DAF\text{-}FM^{+/hi}$	-	5 to 4212	***Cox.2,*** exp(coef) = 2.429, p = 0.031<br>***Cox.4***, exp(coef) = 1.555, p = 0.022
108	$HLA\text{-}DR^{+/lo}DAF\text{-}FM^{+}$	-	79 to 3660	***Wilc.3***, Mean = -0.384, p = 0.005
113	$DAF\text{-}FM^{-/lo}$	-	17 to 4241	***Cox.1,*** exp(coef) = 1.679, p = 0.049
120	$CD11c^{-/lo\ or\ +/lo}HLA\text{-}DR^{+/lo}DAF\text{-}FM^{+/lo}$	-	47 to 3332	***Cox.1,*** exp(coef) = 2.325, p = 0.029<br>***Wilc.3,*** Mean = -0.330, p = 0.033<br>***Cox.4***, exp(coef) = 0.569, p = 0.038
128	$CD11c^{-/lo\ or\ +/lo}HLA\text{-}DR^{-/lo\ or\ +/lo}DAF\text{-}FM^{-/lo}$	-	91 to 6339	***Cox.1,*** exp(coef) = 2.037, p = 0.023
133	$CD11b^{+}CD11c^{+}CD33^{-/lo}HLA\text{-}DR^{+}DAF\text{-}FM^{+}$	-	1 to 1021	***Wilc.3***, Mean = 0.288, p = 0.037
140	$CD11b^{+}CD11c^{+}CD33^{-\ or\ -/lo}HLA\text{-}DR^{+}DAF\text{-}FM^{-/lo}$	-	1 to 2787	***Cox.2,*** exp(coef) = 0.277, p = 0.011<br>***Wilc.2,*** RFS(<1yr, mean = -9.948, SD = 0.484),<br>RFS(>1yr, mean = -9.195, SD = 0.738), p = 0.009<br>***Cox.4,*** exp(coef) = 0.453, p = 0.009<br>***Wilc.4,*** RFS(<1yr, median = -0.883),<br>RFS(>1yr, median = 0.082), p = 0.015
147	$DAF\text{-}FM^{-/lo}$	-	82 to 5684	***Cox.1,*** exp(coef) = 2.361, p = 0.015
151	$CD11c^{+}CD33^{-/lo}HLA\text{-}DR^{+}DAF\text{-}FM^{+}$	-	0 to 797	***Cox.4,*** exp(coef) = 0.466, p = 0.022
155	$CD11b^{-/lo}CD11c^{-/lo}HLA\text{-}DR^{-/lo}DAF\text{-}FM^{+}$	-	129 to 12829	***Cox.4,*** exp(coef) = 2.264, p = 0.020
165	$HLA\text{-}DR^{+}DAF^{-/lo}$	-	27 to 2745	***Wilc.3***, Mean = -0.458, p = 0.014
174	$CD11b^{+}CD11c^{+}DAF\text{-}FM^{+}$	-	20 to 3000	***Cox.4,*** exp(coef) = 1.508, p = 0.025<br>***Wilc.4,*** RFS(<1yr, median = 0.462),<br>RFS(>1yr, median = -0.525), p = 0.046
23	$CD11b^{+/lo}CD11c^{+/lo}CD33^{-/lo}HLA\text{-}DR^{+}DAF\text{-}FM^{+}$	-	24 to 2083	**Cox.4**, exp(coef) = 0.400, p = 0.035
26	$CD11b^{+}CD11c^{+}CD33^{+/lo}CD14^{-/lo}HLA\text{-}DR^{+}DAF\text{-}FM^{-/lo}$	-	3 to 1438	**Cox.4**, exp(coef) = 0.468 , p = 0.019
48	$CD11b^{+/lo}CD11c^{+hi}CD33^{+/lo}CD14^{-\ or\ -/lo}HLA\text{-}DR^{+}DAF\text{-}FM^{+/lo}$	-	9 to 1884	***Cox.4,*** exp(coef) = 0.538, p = 0.043<br>***Wilc.4***, RFS(<1yr, median = -1.245),<br>RFS(>1yr, median = -0.072), p = 0.008

Table S6. Myeloid FSC/SSC cell phenotypes (nodes) associated with treatment effects from the MPATR analysis				
**Node**	**Phenotype**	**Cell Type**	**Cell Range**	**Test**
57	$CD11b^{+/lo}CD11c^{+/lo}CD33^{-\text{ or }-/lo}CD14^{-\text{ or }-/lo}HLA\text{-}DR^{+}DAF\text{-}FM^{-\text{ or }-/lo}$	-	4 to 3834	***Cox.2,*** exp(coef) = 0.478, p = 0.045<br>***Cox.4*** , exp(coef) = 0.543, p = 0.013
71	$CD11b^{+}CD11c^{+}CD33^{-/lo\text{ or }+/lo}HLA\text{-}DR^{+}CD14^{-/lo}DAF\text{-}FM^{-/lo}$	-	0 to 2002	***Cox.1,*** exp(coef) = 1.936, p = 0.034<br>***Cox.4*** , exp(coef) = 0.553, p = 0.019

Table S7a. Representing nodes of PCA analysis of lymphoid population prior to treatment:

Node	Phenotype	Cell Type	λ, (PC1)	% in Component	Statistics
64	CD3^{+}CD8$^{-/lo}$CD56$^{-\text{ or }-/lo}$CD127^{+}CD11c$^{-\text{ or }-/lo}$CD25$^{-/lo}$DAF-FM$^{+/lo}$	Early Effector T cell	-0.12857	1.65302449	NS
13	CD11c^{+}CD25$^{-/lo}$DAF-FM$^{+/lo}$	Antigen presentation cell	-0.121073	1.465867133	***Cox.4,*** exp(coef) = 0.510, p = 0.025, p.adjust = 0.503
84	CD3^{+}CD8^{+}CD127^{+}DAF-FM$^{-/lo}$	CD8 Naïve memory T cell	-0.120714	1.45718698	***Wilc.1*** , RFS(<1yr, mean = -8.208, SD =0.227), RFS(>1yr, mean = -8.537, SD = 0.486), p = 0.038, p.adjust = 1.000, qvalue = 1.000; ***Wilc.2,*** RFS(<1yr, mean = -8.023, SD = 0.544), RFS(>1yr, mean = -8.414, SD = 0.528), p = 0.037, qvalue = 0.832;
167	CD11c^{+}CD25$^{-/lo\text{ or }+/lo}$DAF-FM$^{+/lo}$	Antigen presentation cell	-0.117463	1.379755637	***Cox.4,*** exp(coef) = 0.397, p = 0.040, p.adjust =0.522
192	CD11c^{+}CD25$^{+/lo}$DAF-FM$^{+/lo}$	Antigen presentation cell	-0.116881	1.366116816	NS
80	CD8$^{+/lo}$CD56^{+}CD11c$^{+/lo}$CD25$^{-/lo}$DAF-FM$^{+/lo\text{ or }-/lo}$	NK Cell	-0.116032	1.346342502	NS
37	CD11c$^{+/lo\text{ or }-/lo}$CD56$^{-/lo}$CD25$^{-/lo}$DAF-FM$^{+/lo}$	Antigen presentation cell	-0.116005	1.345716003	NS
86	CD19^{+}CD25$^{-/lo}$DAF-FM$^{-/lo\text{ or }+/lo}$	B cell	-0.115658	1.337677296	***Cox.1,*** exp(coef) = 1.777, p = 0.034, p.adjust = 0.949; ***Cox.4,*** exp(coef) = 0.668, p = 0.030, p.adjust = 0.503;
134	CD8$^{-/lo}$CD56$^{+/lo}$CD11c$^{-/lo}$CD25$^{-/lo}$DAF-FM$^{-/lo}$	NK Cell	-0.115638	1.337214704	NS
23	CD3^{+}CD4^{+}CD127^{+}CD25$^{-/lo}$DAF-FM$^{+/lo}$	Effector memory T cell	-0.113195	1.281310803	NS
106	CD3^{+}CD8$^{-/lo}$CD127^{+}CD11c^{-}CD25^{-}DAF-FM$^{-/lo}$	Early Effector T cell	-0.113148	1.28024699	NS
115	CD19^{+}CD11c$^{-/lo}$CD25$^{-/lo}$DAF-FM$^{-/lo}$	B cell	-0.111091	1.234121028	***Cox.4,*** exp(coef) = 0.677, p = 0.047, p.adjust = 0.522
69	CD3^{+}CD8^{+}CD127$^{-/lo}$DAF-FM^{+}	Effector T Cell	0.110768	1.226954982	***Wilc.3,*** Mean = -0.370 , p = 0.012, p.adjust = 0.225
3	CD19^{+}CD25$^{-/lo}$DAF-FM$^{-/lo}$	B cell	-0.107885	1.163917323	***Cox.1,*** exp(coef) = 1.64, p = 0.049, p.adjust = 0.949; ***Cox.4,*** exp(coef) = 0.666, p = 0.046, p.adjust = 0.522
96	CD11c^{+}CD56^{+}DAF-FM$^{-/lo}$	NK cell	-0.107822	1.162558368	NS
63	CD11c^{+}CD56^{+}CD25$^{-/lo}$DAF-FM$^{-/lo}$	NK cell	-0.104649	1.09514132	NS
68	CD11c^{+}CD8$^{+/lo\text{ or }-/lo}$CD4$^{-/lo}$CD25$^{+/lo}$DAF-FM^{+}	Early T cell	0.104117	1.084034969	NS
109	CD11c^{+}CD8$^{+/lo}$CD56^{+}CD25$^{-/lo}$DAF-FM$^{-/lo}$	NK Cell	-0.104015	1.081912023	NS
32	CD11c^{+}CD4$^{-/lo}$CD25$^{+/lo}$DAF-FM^{+}	Antigen presentation cell	-0.103938	1.080310784	NS
28	CD3^{+}CD127$^{-\text{ or }-/lo}$DAF-FM$^{-/lo}$	αβ T cell	-0.103613	1.073565377	NS
56	CD3^{+}CD8^{+}CD127^{+}CD25$^{-/lo}$DAF-FM^{+}	Effector T cell	0.103241	1.065870408	NS
141	CD3^{+}CD8$^{+/lo}$CD56$^{+/lo}$CD127^{+}CD11c$^{-/lo}$CD25$^{-/lo}$DAF-FM$^{-/lo}$	NK cell	-0.102978	1.060446848	NS
26	CD3^{+}CD8^{+}CD127^{+}DAF-FM$^{-/lo}$	Naïve CD8 or memory T cell	-0.102875	1.058326563	***Cox.1,*** exp(coef) = 3.635, p = 0.031, p.adjust = 0.949; ***Wilc.1,*** RFS(<1yr, mean = -7.843, SD =0.402), RFS(>1yr, mean = -8.227, SD = 0.506), p = 0.038, p.adjust = 1.000, qvalue = 1.000; ***Wilc.2*** , RFS(<1yr, mean = -7.750, SD =0.611), RFS(>1yr, mean = -8.216, SD = 0.549), p = 0.022, qvalue = 0.832;
50	CD3^{+}DAF-FM$^{-/lo}$	αβ T cell	-0.102758	1.055920656	NS
197	CD11c^{+}CD56$^{-\text{ or }-/lo}$CD4$^{-/lo}$CD25$^{+/lo}$DAF-FM$^{+/lo}$	Antigen presentation cell	-0.102358	1.047716016	***Wilc.3,*** Mean = 0.357, p = 0.012, p.adjust = 0.225; ***Cox.4,*** exp(coef) = 0.368, p = 0.018, p.adjust =0.503
114	CD11c^{+}CD25$^{+/lo}$DAF-FM$^{+/low}$	Antigen presentation cell	-0.102015	1.040706023	NS
19	CD3^{+}CD8^{+}CD127$^{-/lo}$CD11c$^{-/lo}$DAF-FM^{+}	Effector memory CD8 T cell	0.101956	1.039502594	NS
180	CD3^{+}CD127$^{+/lo}$DAF-FM$^{+/lo}$	αβT Cell	-0.101863	1.037607077	***Wilc.2,*** RFS(<1yr, mean = -10.618, SD = 0.387), RFS(>1yr, mean = -11.174, SD = 0.734), p = 0.010, qvalue = 0.832;
40	CD11c^{+}CD25$^{-/lo}$DAF-FM$^{+/lo}$	Antigen presentation cell	-0.101681	1.033902576	NS
72	CD3^{+}CD4^{+}CD127^{+}CD25$^{-/lo}$DAF-FM$^{-/lo}$	Effector memory T cell	-0.101509	1.030407708	NS
10	CD11c^{+}CD19^{+}CD25$^{+/lo}$DAF-FM$^{+/lo}$	B cell	-0.101437	1.028946497	NS
155	CD3^{+}CD8^{+}CD25$^{-/lo}$DAF-FM^{+}	Effector T Cell	0.100911	1.018302992	***Wilc.1,*** RFS(<1yr, mean = -8.666, SD =0.694), RFS(>1yr, mean = -8.066, SD = 0.956), p = 0.035, p.adjust = 1.000, qvalue = 1.000
65	CD11c$^{-/lo}$CD56^{+}DAF-FM$^{-/lo}$	NK cell	-0.100804	1.016144642	NS
194	CD11c$^{+/lo}$CD25$^{-/lo}$DAF-FM^{lo}	Antigen presentation cell	-0.100227	1.004545153	NS

Table S7b. Representing nodes of PCA analysis of lymphoid population (post-pre):

Node	Phenotype	Cell Type	λ, (PC1)	% in Component	Statistics
126	CD8^{+}CD56^{+}CD11c^{+}DAF-FM^{+}	CD8^{+} NK cell	0.112988	1.276628814	***Wilc.3,*** Mean = 0.382, p = 0.012, p.adjust = 0.225; ***Cox.4,*** exp(coef) = 1.484, p = 0.040, p.adjust = 0.522
24	CD11c$^{+/lo}$CD56^{+}DAF-FM^{+}	NK cell	0.112028	1.255027278	***Cox.4*** , exp(coef) = 1.610, p = 0.019, p.adjust = 0.503 ***Wilc.4*** , RFS(<1yr, median = 0.438), RFS(>1yr, median = -0.506), p = 0.017, p.adjust = 0.686, qvalue = 0.686
122	CD8^{+}CD56^{+}CD11c^{+}CD25$^{-/lo}$DAF-FM^{+}	CD8^{+} NK cell	0.111521	1.243693344	***Wilc.3,*** Mean = 0.343, p = 0.011, p.adjust = 0.225 ***Wilc.4,*** RFS(<1yr, median = 0.271), RFS(>1yr, median = -0.575), p = 0.037, p.adjust = 0.686, qvalue = 0.686
127	CD11c$^{+/lo}$CD56^{+}CD8$^{-/lo}$CD25$^{-/lo}$DAF-FM^{+}	NK cell	0.110914	1.23019154	NS
102	CD8$^{+/lo}$CD11c^{+}CD56^{+}CD25$^{-/lo}$DAF-FM^{+}	NK cell	0.109008	1.188274406	NS
42	CD11c^{+}CD56$^{+/lo}$CD25$^{-/lo}$DAF-FM^{+}	NK cell	0.108139	1.169404332	***Cox.4*** , exp(coef) = 1.659, p =0.018, p.adjust = 0.503 ***Wilc.4*** , RFS(<1yr, median = 0.670), RFS(>1yr, median = -0.444), p = 0.005, p.adjust = 0.686, qvalue = 0.686
130	CD11c^{+}CD8$^{-/lo}$CD56^{+}CD25$^{-/lo}$DAF-FM^{+}	NK cell	0.1057	1.117249	NS
136	CD11c^{+}CD8$^{-/lo}$CD56^{+}CD25$^{-/lo}$DAF-FM^{+}	NK cell	0.105565	1.114396923	NS
132	CD8$^{+/lo}$CD11c^{+}CD56^{+}CD25$^{-/lo}$DAF-FM^{+}	CD8^{+} NK cell	0.104518	1.092401232	***Wilc.3,*** Mean = 0.347, p = 0.012, p.adjust = 0.225
157	CD11c$^{-/lo}$CD8$^{-/lo}$CD56^{+}CD25$^{-/lo}$DAF-FM^{+}	NK cell	0.104422	1.090395408	NS
17	CD11c^{+}CD8$^{-/lo}$CD56^{+}CD25$^{-/lo}$DAF-FM^{+}	NK cell	0.103156	1.064116034	NS
176	CD3^{+}CD8^{+}CD127^{+}CD25$^{-/lo}$DAF-FM^{+}	Effector CD8 T cell	0.103095	1.062857903	NS
51	CD8^{+}CD56^{+}CD11c$^{+/lo}$CD25$^{-/lo}$DAF-FM^{+}	NK cell	0.102657	1.053845965	NS
27	CD8$^{+/lo}$CD11c^{+}CD56^{+}DAF-FM^{+}	CD8^{+} NK cell	0.101684	1.033963586	***Wilc.3,*** Mean = 0.319 , p = 0.014, p.adjust = 0.228
53	CD3^{+}CD8^{+}CD127$^{+/lo}$DAF-FM^{+}	CD8 Naive or Memory T Cell	0.100388	1.007775054	***Cox.2,*** exp(coef) = 2.787, p = 0.047, p.adjust = 0.989; ***Wilc.2*** , RFS(<1yr, mean = -6.628, SD =0.614), RFS(>1yr, mean = -7.191, SD = 0.642), p = 0.030, qvalue = 0.832

Table S7c. Representing nodes of PCA analysis of myeloid population prior to treatment:

Node	Phenotype	Cell Type	λ, (PC1)	% in Component	Statistics
44	CD11c^{+}CD11b^{+}CD33$^{-/lo}$HLA-DR$^{+/lo}$DAF-FM$^{-/lo}$	Dendritic Cell	0.121667	1.480285889	NS
61	CD11c$^{\text{- or -/lo}}$HLA-DR$^{\text{-/lo or +/lo}}$DAF-FM$^{+/lo}$	-	0.119103	1.418552461	***Wilc.3,*** Mean = -0.442, p = 0.004, p.adjust = 0.213
23	CD11b$^{+/lo}$CD11c$^{-/lo}$CD33$^{-/lo}$HLA-DR$^{+/lo}$DAF-FM$^{+/lo}$	-	0.115254	1.328348452	NS
178	CD33^{+}CD11b^{+}CD11c^{+}CD14$^{-/lo}$HLA-DR^{+}DAF-FM$^{+/lo}$	Dendritic Cell	0.114943	1.321189325	***Cox.4,*** exp(coef) =0.360 p = 0.016, p.adjust = 0.216
183	CD11b$^{+/lo}$CD11c$^{+/lo}$CD15$^{\text{- or -/lo}}$CD33$^{-/lo}$HLA-DR^{+}DAF-FM$^{+/lo}$	Dendritic Cell	0.114827	1.318523993	NS
98	HLA-DR^{+}DAF-FM$^{+/lo}$	-	0.114525	1.311597563	NS
199	CD11b^{+}CD11c^{+}CD33$^{+/lo}$CD14$^{+/lo}$HLA-DR^{+}DAF-FM$^{-/lo}$	Monocyte derived dendritic cell	0.114277	1.305923273	NS
111	CD11b^{+}CD11c$^{+/lo}$CD33$^{\text{-/lo or +/lo}}$HLA-DR$^{\text{+ or +/lo}}$DAF-FM$^{-/lo}$	Dendritic Cell	0.113995	1.299486003	***Cox.4,*** exp(coef) = 0.420 ,p = 0.023 , p.adjust = 0.216
85	HLA-DR^{+}DAF-FM$^{+/lo}$	-	0.113271	1.283031944	***Cox.4,*** exp(coef) = 0.543, p = 0.029, p.adjust = 0.216
137	HLA-DR^{+}CD33^{+}CD11b^{+}CD11c^{+}CD14^{+}DAF-FM$^{+/lo}$	Monocyte derived dendritic cell vs Monocytic	0.112484	1.265265026	***Cox.1,*** exp(coef) = 1.873 , p = 0.039, p.adjust = 0.552;<br>***Wilc.1,*** RFS(<1yr, mean = -9.877, SD =0.992), RFS(>1yr, mean = -10.522, SD = 0.778), p = 0.038, p.adjust = 0.986, qvalue = 0.986;<br>***Cox.4,*** exp(coef) =0.492, p = 0.009, p.adjust = 0.216<br>***Wilc.4,*** RFS(<1yr, median = -0.618), RFS(>1yr, median = -0.011), p = 0.041, p.adjust = 0.869, qvalue = 0.862
105	CD11b$^{\text{-/lo or +/lo}}$CD11c$^{-/lo}$HLA-DR^{+}DAF-FM$^{-/lo}$	-	0.11192	1.25260864	***Cox.4,*** exp(coef) = 0.643, p = 0.033, p.adjust = 0.216
179	CD11b$^{\text{-/lo or +/lo}}$CD11c$^{\text{-/lo or +/lo}}$CD33$^{-/lo}$HLA-DR$^{\text{-/lo or +/lo}}$DAF-FM$^{-/lo}$	-	0.111773	1.249320353	***Cox.1,*** exp(coef) = 2.539 , p = 0.036 , p.adjust = 0.55;<br>***Cox.4,*** exp(coef) = 0.515 ,p = 0.045 , p.adjust = 0.239
32	CD33^{+}CD11b^{+}CD11c^{+}CD14^{+}HLA-DR^{+}DAF-FM$^{+/lo}$	Monocytic	0.111466	1.242466916	***Cox.4*** , exp(coef) = 0.492, p = 0.022, p.adjust = 0.216
5	CD11b^{+}CD11c^{+}CD33$^{+/lo}$HLA-DR^{+}DAF-FM$^{+/lo}$	Dendritic Cell	0.11092	1.23032464	NS
116	HLA-DR^{+}DAF-FM$^{-/lo}$	-	0.110547	1.222063921	NS
95	HLA-DR$^{\text{-/lo or +/lo}}$DAF-FM$^{-/lo}$	-	0.109515	1.199353523	***Cox.1,*** exp(coef) = 2.029, p = 0.028 , p.adjust = 0.552
12	CD11b^{+}CD11c^{+}CD33^{+}HLA-DR^{+}DAF-FM^{+}	Dendritic Cell	0.109478	1.198543248	NS
195	CD11c^{+}CD11b$^{+/lo}$HLA-DR^{+}DAF-FM$^{-/lo}$	Dendritic Cell	0.109477	1.198521353	***Cox.1,*** exp(coef) = 1.821, p = 0.034 p.adjust = 0.552;<br>***Cox.4,*** exp(coef) = 0.489, p = 0.004, p.adjust = 0.216
164	DAF-FM$^{-/lo}$	-	0.108955	1.187119203	NS
64	CD11b^{+}CD11c^{+}CD33$^{-/lo}$HLA-DR^{+}DAF-FM$^{\text{-/lo or +/lo}}$	Dendritic Cell	0.10849	1.17700801	***Cox.4*** , exp(coef) = 0.439 p = 0.017, p.adjust = 0.216
3	CD11b^{+}CD11c^{+}HLA-DR$^{+/lo}$DAF-FM$^{-/lo}$	Dendritic Cell	0.107908	1.164413646	NS
96	HLA-DR^{+}DAF-FM$^{-/lo}$	-	0.107873	1.163658413	***Cox.4,*** exp(coef) = 0.593, p = 0.022, p.adjust = 0.216
43	DAF-FM$^{-/lo}$	-	0.107706	1.160058244	***Cox.1*** ,exp(coef) = 2.538, p = 0.015, p.adjust = 0.446
190	CD11b^{+}HLA-DR^{+}DAF-FM$^{\text{-/lo or +/lo}}$	-	0.107361	1.152638432	***Cox.2,*** exp(coef) = 0.347, p = 0.028, p.adjust = 0.940;<br>***Wilc.2*** , RFS(<1yr, mean = -11.425, SD =0.612), RFS(>1yr, mean = -10.904, SD = 0.638), p = 0.046, p.adjust = 0.999, qvalue = 0.999<br>***Cox.4,*** exp(coef) = 0.527, p = 0.020 , p.adjust = 0.216
153	CD33$^{\text{-/lo or +/lo}}$CD11b^{+}CD11c^{+}HLA-DR$^{+/lo}$DAF-FM$^{\text{-/lo or +/lo}}$	Dendritic Cell	0.107337	1.152123157	***Cox.4,*** exp(coef) =0.452, p = 0.038, p.adjust = 0.216
47	CD33$^{\text{-/lo or +/lo}}$CD11b^{+}CD11c^{+}CD14-/loHLA-DR^{+}DAF-FM$^{-/lo}$	Dendritic Cell	0.107156	1.148240834	***Cox.1*** , exp(coef) = 1.882 , p = 0.037 , p.adjust = 0.552;<br>***Cox.4*** , exp(coef) = 0.599 ,p = 0.036 , p.adjust = 0.216
62	HLADR$^{+/lo}$DAF-FM$^{-/lo}$	-	0.106869	1.142098316	NS
114	CD11b^{+}CD11c^{+}DAF-FM$^{-/lo}$	Antigen Presentation Cell	0.105949	1.12251906	***Wilc.3,*** Mean = 0.316, p = 0.017, p.adjust = 0.332
177	CD11c$^{-/lo}$HLA-DR^{+}DAF-FM$^{\text{-/lo or +/lo}}$	-	0.104785	1.097989623	***Cox.4,*** exp(coef) =0.540 p = 0.013, p.adjust = 0.216
59	CD11$^{\text{c- or -/lo}}$HLA-DR$^{\text{- or -/lo}}$DAF-FM-/lo	-	0.10412	1.08409744	***Cox.1*** ,exp(coef) = 2.769, p = 0.014, p.adjust = 0.446
188	CD11b$^{+/lo}$CD11c$^{+/lo}$CD33$^{-/lo}$HLA-DR$^{+/lo}$DAF-FM$^{-/lo}$	Dendritic Cell	0.103986	1.08130882	NS
89	CD11b$^{-/lo}$HLA-DR^{+}DAF-FM$^{-/lo}$	-	0.10368	1.07495424	***Cox.1,*** exp(coef) = 1.918, p = 0.037 , p.adjust = 0.552;<br>***Cox.4,*** exp(coef) = 0.636 ,p = 0.042 , p.adjust = 0.233
18	CD11b^{+}CD11c^{+}CD33^{+}CD14^{+}HLA-DR^{+}DAF-FM^{+}	Monocytic	0.103656	1.074456634	***Cox.4*** , exp(coef) = 0.610, p = 0.023, p.adjust = 0.216
120	HLA-DR$^{\text{- or -/lo}}$DAF-FM$^{-/lo}$	-	0.103652	1.07437371	***Cox.1*** ,exp(coef) = 2.208, p = 0.013, p.adjust = 0.446
86	CD11b^{+}CD11c^{+}CD33$^{-/lo}$HLA-DR$^{+/lo}$DAF-FM$^{-/lo}$	Antigen Presentation Cell	0.10362	1.07371044	***Cox.4,*** exp(coef) = 0.425, p = 0.027, p.adjust = 0.216
40	CD11b$^{-/lo}$CD11c$^{-/lo}$HLA-DR^{+}DAF-FM$^{-/lo}$	-	0.10355	1.07226025	***Cox.1,*** exp(coef) = 2.468, p = 0.011, p.adjust = 0.446<br>***Cox.4*** , exp(coef) = 0.502, p = 0.138, p.adjust = 0.216
10	CD11b^{+}CD11c^{+}HLA-DR$^{-/lo}$DAF-FM$^{-/lo}$	Early Antigen Presentation Cell	0.10319	1.06481761	***Wilc.3,*** Mean = 0.315 , p = 0.042, p.adjust = 0.401
24	CD11b$^{\text{- or -/lo}}$HLA-DR$^{\text{- or -/lo}}$DAF-FM$^{\text{- or -/lo}}$	-	0.103015	1.061209023	***Cox.1*** ,exp(coef) = 2.220, p = 0.013, p.adjust = 0.446
78	CD11b$^{+/lo}$CD11c^{+}CD33^{+}CD14$^{+/lo}$HLA-DR^{+}DAF-FM$^{+/lo}$	Monocytic	0.102856	1.057935674	***Wilc.1*** , RFS(<1yr, mean = -9.348, SD =0.851), RFS(>1yr, mean = -10.140, SD = 1.179), p = 0.033, p.adjust = 0.986, qvalue = 0.986<br>***Cox.4*** , exp(coef) = 0.643, p = 0.029, p.adjust = 0.216;<br>***Wilc.4*** , RFS(<1yr, median = -1.289), RFS(>1yr, median = -0.243), p = 0.037, p.adjust = 0.869, qvalue = 0.862
160	CD11b$^{+/lo}$CD11c^{+}CD33$^{-/lo}$HLA-DR^{+}DAF-FM$^{\text{-/lo or +/lo}}$	-	0.102155	1.043564403	***Wilc.2,*** RFS(<1yr, mean = -9.984, SD =0.359), RFS(>1yr, mean = -9.335, SD = 0.831), p = 0.022, p.adjust = 0.999, qvalue = 0.999;<br>***Cox.4,*** exp(coef) =0.548 p = 0.034, p.adjust = 0.216
155	CD33$^{+/lo}$CD11b$^{-/lo}$CD11c^{+}HLA-DR^{+}DAF-FM$^{+/lo}$	Plasmacytoid DC (CD11b-/lo)	0.101804	1.036405442	***Cox.4,*** exp(coef) =0.450 p = 0.021, p.adjust = 0.216<br>**Wilc.4**, RFS(<1yr, median = -0.787), RFS(>1yr, median = -0.050), p = 0.019, p.adjust = 0.869, qvalue = 0.862
34	CD11b^{+}CD11c$^{+/lo}$CD33$^{\text{-/lo or +/lo}}$HLA-DR$^{-/lo}$DAF-FM$^{-/lo}$	MDSC	0.101436	1.02892621	NS
189	CD11b$^{\text{-/lo or +/lo}}$CD11c$^{\text{-/lo or +/lo}}$HLA-DR^{+}DAF-FM$^{\text{+ or +/lo}}$	-	0.100894	1.017959924	***Cox.1,*** exp(coef) = 2.779 , p = 0.016 , p.adjust = 0.446;<br>***Cox.4,*** exp(coef) = 0.256, p = 0.005 , p.adjust = 0.216
172	CD11b$^{+/lo}$HLA-DR$^{+/lo}$DAF-FM$^{-/lo}$	-	0.100434	1.008698836	NS

Table S7d. Representing nodes of PCA analysis of myeloid population (post-pre):

Node	Phenotype	Cell Type	λ, (PC1)	% in Component	Statistics
23	CD11b$^{+/lo}$CD11c$^{-/lo}$HLA-DR$^{+/lo}$DAF-FM$^{-/lo}$	-	-0.108298	1.17284568	NS
44	CD11c$^{+/lo}$CD11b$^{+/lo}$CD33$^{-/lo}$HLA-DR$^{+/lo}$DAF-FM$^{-/lo}$	-	-0.107762	1.161264864	NS
183	CD11b$^{+/lo}$CD11c$^{+/lo}$CD15$^{- \text{ or } -/lo}$CD33$^{-/lo}$HLADR$^{+/lo}$DAF-FM$^{-/lo}$	Dendritic Cell	-0.107233	1.149891629	NS
113	CD11b^{+}C11c$^{-/lo}$HLA-DR$^{-/lo}$DAF-FM$^{-/lo}$	-	-0.106954	1.143915812	NS
179	CD11b$^{-/lo \text{ or } +/lo}$CD11c$^{-/lo \text{ or } +/lo}$CD33$^{-/lo}$HLA-DR$^{-/lo \text{ or } +/lo}$DAF-FM$^{-/lo}$	MDSC	0.111773	1.249320353	***Cox.1,*** exp(coef) = 2.539 , p = 0.036 , p.adjust = 0.55;<br>***Cox.4,*** exp(coef) = 0.515 ,p = 0.045 , p.adjust = 0.239
59	CD11c$^{- \text{ or } -/lo}$HLA-DR$^{- \text{ or } -/lo}$DAF-FM$^{-/lo}$	-	0.10412	1.08409744	***Cox.1*** ,exp(coef) = 2.769, p = 0.014, p.adjust = 0.446
24	CD11b$^{- \text{ or } -/lo}$HLA-DR$^{- \text{ or } -/lo}$DAF-FM$^{- \text{ or } -/lo}$	-	0.103015	1.061209023	***Cox.1*** ,exp(coef) = 2.220, p = 0.013, p.adjust = 0.446
67	CD11b^{+}CD11c^{+}HLA-DR$^{-/lo}$DAF-FM^{+}	Dendritic Cell	0.105253	1.107819401	***Cox.4*** , exp(coef) = 1.549, p = 0.020, p.adjust = 0.216
43	DAF-FM$^{-/lo}$	-	-0.105049	1.10352924	***Cox.1*** ,exp(coef) = 2.538, p = 0.015, p.adjust = 0.446
111	CD11b^{+}CD11c$^{+/lo}$CD33$^{-/lo \text{ or } +/lo}$HLA-DR$^{+ \text{ or } +/lo}$DAF-FM$^{-/lo}$	Dendritic Cell	0.113995	1.299486003	***Cox.4,*** exp(coef) = 0.420 ,p = 0.023 , p.adjust = 0.216
64	CD11b^{+}CD11c^{+}CD33$^{-/lo}$HLA-DR^{+}DAF-FM$^{-/lo \text{ or } +/lo}$	Dendritic Cell	0.10849	1.17700801	***Cox.4*** , exp(coef) = 0.439 p = 0.017, p.adjust = 0.216
199	CD11b^{+}CD11c^{+}CD33$^{+/lo}$CD14$^{+/lo}$HLA-DR^{+}DAF-FM$^{-/lo}$	Dendritic Cell	0.114277	1.305923273	NS
45	DAF-FM^{+}	-	0.104049	1.08261944	***Cox.4,*** exp(coef) =2.391, p = 0.006, p.adjust = 0.216
195	CD11c^{+}CD11b$^{+/lo}$HLA-DR^{+}DAF-FM$^{-/lo}$	Dendritic Cell	0.109477	1.198521353	***Cox.1,*** exp(coef) = 1.821, p = 0.034 p.adjust = 0.552;<br>***Cox.4,*** exp(coef) = 0.489, p = 0.004, p.adjust = 0.216
172	CD11b$^{+/lo}$HLA-DR$^{+/lo}$DAF-FM$^{-/lo}$	-	0.100434	1.008698836	NS
34	CD11b^{+}CD11c$^{+/lo}$CD33$^{-/lo \text{ or } +/lo}$HLA-DR$^{-/lo}$DAF-FM$^{-/lo}$	MDSC	0.101436	1.02892621	NS
160	CD11b$^{+/lo}$CD11c^{+}CD33$^{-/lo}$HLA-DR^{+}DAF-FM$^{-/lo \text{ or } +/lo}$	Dendritic Cell	0.102155	1.043564403	***Wilc.2,*** RFS(<1yr, mean = -9.984, SD =0.359), RFS(>1yr, mean = -9.335, SD = 0.831), p = 0.022, p.adjust = 0.999, qvalue = 0.999;<br>***Cox.4,*** exp(coef) =0.548 p = 0.034, p.adjust = 0.216
5	CD11b^{+}CD11c^{+}CD33$^{+/lo}$HLA-DR^{+}DAF-FM$^{-/lo}$	Dendritic Cell	0.11092	1.23032464	NS
95	HLA-DR$^{-/lo \text{ or } +/lo}$DAF-FM$^{-/lo}$	-	0.109515	1.199353523	***Cox.1,*** exp(coef) = 2.029, p = 0.028 , p.adjust = 0.552
86	CD11b^{+}CD11c^{+}CD33$^{-/lo}$HLA-DR$^{+/lo}$DAF-FM$^{-/lo}$	Dendritic Cell	0.10362	1.07371044	***Cox.4,*** exp(coef) = 0.425, p = 0.027, p.adjust = 0.216
50	CD11b$^{+/lo}$CD11c$^{-/lo}$HALDR$^{-/lo}$DFA-FM$^{+/lo}$	-	-0.100639	1.012820832	NS
32	CD33^{+}CD11b^{+}CD11c^{+}CD14^{+}HLA-DR^{+}DAF-FM$^{+/lo}$	Monocytic	0.111466	1.242466916	***Cox.4*** , exp(coef) = 0.492, p = 0.022, p.adjust = 0.216
188	CD11b$^{+/lo}$CD11c$^{+/lo}$CD33$^{-/lo}$HLA-DR$^{+/lo}$DAF-FM$^{-/lo}$	Dendritic Cell	0.103986	1.08130882	NS

Table S8a. Representing nodes of PLS analysis of lymphoid population prior to treatment:

Node	Phenotype	Cell Type	W_1
44	$CD3^{+}CD8^{+}CD127^{+/lo}DAF\text{-}FM^{+}$	Effector CD8+ T cell	-0.180858
121	$CD11c^{+}CD56^{-/lo}CD25^{+/lo}DAF\text{-}FM^{+/lo}$	Antigen Presentation cell	-0.0863652
8	$CD3^{+}CD56^{+/lo}CD127^{+/lo}CD11c^{-\text{ or }-/lo}CD25^{-/lo}DAF\text{-}FM^{+}$	NK-like cell	0.169409
175	$CD3^{+}CD4^{+}CD127^{+}CD25^{-/lo\text{ or }+/lo}DAF\text{-}FM^{+}$	Effector memory T cell	0.161053
145	$CD3^{+}CD4^{+}CD127^{+}CD25^{-/lo}DAF\text{-}FM^{+}$	CD4+ Effector memory T cell	0.153607
105	$CD3^{+}CD8^{+}CD127^{+/lo}CD25^{-/lo}DAF\text{-}FM^{+}$	Effector CD8 T cell	-0.152812
36	$CD3^{+}CD127^{+/lo}DAF\text{-}FM^{+}$	alphabeta transitioning	0.151402
187	$CD127^{-/lo}CD4^{-/lo}CD11c^{+/lo}CD25^{+/lo}DAF\text{-}FM^{+}$	Antigen Presentation cell	-0.148933

Table S8b. Representing nodes of PLS analysis of lymphoid population post treatment:

Node	Phenotype	Cell Type	W_1
44	CD3^{+}CD8^{+}CD127$^{+/lo}$DAF-FM^{+}	Effector CD8 T cell	-0.194458
103	CD3^{+}CD8^{+}CD56$^{+/lo}$CD11c$^{+/lo}$CD25$^{-/lo}$DAF-FM$^{+/lo}$	NK-like cell	0.180444
105	CD3^{+}CD8^{+}CD127$^{+/lo}$CD25$^{-/lo}$DAF-FM^{+}	Effector CD8 T cell	-0.175536
190	CD3^{+}CD56+CD8^{+}CD11c$^{-/lo}$DAF-FM$^{-/lo}$	NK-like cell	0.171347
77	CD11c$^{+/lo}$CD25$^{-/lo}$DAF-FM$^{+/lo}$	Antigen presentation cell	-0.160882
187	CD127$^{-/lo}$CD4$^{-/lo}$CD11c$^{+/lo}$CD25$^{+/lo}$DAF-FM+	Antigen presentation cell	-0.15396
16	CD11c$^{+/lo}$CD25+/loDAF-FM^{+}	Antigen presentation cell	-0.153146
121	CD11c^{+}CD56$^{-/lo}$CD25$^{+/lo}$DAF-FM$^{+/lo}$	Antigen presentation cell	-0.151428
176	CD3^{+}CD8^{+}CD127^{+}CD11c$^{-/lo}$CD25$^{+/lo}$DAF-FM^{+}	Effector CD8 T cell	-0.151061
53	CD3^{+}CD8^{+}CD127$^{+/lo}$DAF-FM^{+}	Effector CD8 T cell	-0.147241
14	CD3^{+}CD8^{+}CD127$^{-/lo}$CD25$^{-\text{ or }-/lo}$DAF-FM^{+}	Effector CD8 T cell	-0.143333
57	CD11c$^{+/lo}$CD127^{-}CD25$^{+/lo\text{ or }-/lo}$DAF-FM$^{+/lo}$	Antigen presentation cell	-0.142374

Table S8c. Representing nodes of PLS analysis of myeloid population prior to treatment:

Node	Phenotype	Cell Type	W_1
122	$CD11b^{+\ or\ +/lo}DAF\text{-}FM^{+}$	-	0.157069
166	$CD11b^{+}HLA\text{-}DR^{-/lo}DAF\text{-}FM^{+}$	-	0.144365

Table S8d. Representing nodes of PLS analysis of myeloid population post treatment:

Node	Phenotype	Cell Type	W_1
130	$CD11b^{+/lo}CD33^{-\ or\ -/lo}CD11c^{+/lo}HLA\text{-}DR^{+/lo}DAF\text{-}FM^{+}$	Antigen Presentation cell	-0.203375
36	$CD11b^{+}CD11c^{+}HLA\text{-}DR^{-/lo\ or\ +/lo}DAF\text{-}FM^{+}$	MDSC	0.159404
122	$CD11b^{+\ or\ +/lo}DAF\text{-}FM^{+}$	CD11b+ cells	0.15678
25	$CD11b^{+}CD11c^{-/lo}HLA\text{-}DR^{+/lo}DAF\text{-}FM^{+}$	CD11b+ cells	0.154601
42	$CD11b^{+}CD11c^{+/lo}HLA\text{-}DR^{-/lo}DAF\text{-}FM^{+}$	Antigen Presentation cell	0.150295
193	$HLA\text{-}DR^{+}DAF\text{-}FM^{+}$	MHCII positive cell	0.148774
200	$DAF\text{-}FM^{-/lo}$	-	-0.146963
163	$CD11b^{+/lo}CD11c^{-\ or\ -/lo}HLA\text{-}DR^{+}DAF\text{-}FM^{+}$	CD11b+ cells	0.144359

Table S9. FoxP3^{+} cells among CD3^{+}CD4^{+}CD25^{+}CD127$^{-/lo}$ population (Node 159):

Sample	RFS	% FoxP3^{+} Cells	Total events
15 post	91	97.12	1920
15 pre	91	90.97	1512
25 post	137	90.47	1319
25 pre	137	92.39	1214
30 post	176	95.51	1361
30 pre	176	85.51	1103
32 post	1023	89.95	1540
32 pre	1023	99.04	310
26 post	1155	86.12	1775
26 pre	1155	86.47	1343
35 post	1518	93.96	746
35 pre	1518	91.1	1793
11 post	1548	94.41	846
11 pre	1548	90.14	1448
